# New C_15_ Acetogenins from Two Species of *Laurencia* from the Aegean Sea

**DOI:** 10.3390/molecules27061866

**Published:** 2022-03-13

**Authors:** Maria Harizani, Dafni-Ioanna Diakaki, Stamatios Perdikaris, Vassilios Roussis, Efstathia Ioannou

**Affiliations:** Section of Pharmacognosy and Chemistry of Natural Products, Department of Pharmacy, School of Health Sciences, National and Kapodistrian University of Athens, Panepistimiopolis Zografou, 15771 Athens, Greece; mariachariz@pharm.uoa.gr (M.H.); dafnid@pharm.uoa.gr (D.-I.D.); takiperd@hotmail.com (S.P.); roussis@pharm.uoa.gr (V.R.)

**Keywords:** *Laurencia microcladia*, *Laurencia obtusa*, red algae, C_15_ acetogenins, isolation, structure elucidation, Aegean Sea, antibacterial activity

## Abstract

The chemical diversity of the approximately 1,200 natural products isolated from red algae of the genus *Laurencia*, in combination with the wide range of their biological activities, have placed species of *Laurencia* in the spotlight of marine chemists’ attention for over 60 years. The chemical investigation of the organic (CH_2_Cl_2_/MeOH) extracts of *Laurencia microcladia* and *Laurencia obtusa*, both collected off the coasts of Tinos island in the Aegean Sea, resulted in the isolation of 32 secondary metabolites, including 23 C_15_ acetogenins (**1**–**23**), 7 sesquiterpenes (**24**–**30**) and 2 diterpenes (**31** and **32**). Among them, six new C_15_ acetogenins, namely 10-acetyl-sagonenyne (**2**), *cis*-sagonenyne (**3**), *trans*-thuwalenyne C (**4**), tinosallene A (**11**), tinosallene B (**12**) and obtusallene XI (**17**), were identified and their structures were elucidated by extensive analysis of their spectroscopic data. Compounds **1**–**3**, **5**–**11**, **13** and **15**–**32** were evaluated for their antibacterial activity against *Staphylococcus aureus* and *Escherichia coli*.

## 1. Introduction

Red algae of the genus *Laurencia* can be found in temperate to tropical shores around the world, inhabiting littoral to sublittoral zones and spreading to depths down to 65 m. The plasticity of their distinction markers has led to debates and frequent taxonomic revisions of the genus, which currently includes 137 accepted species [1]. Species of the genus *Laurencia* have been extensively investigated for over 60 years, resulting in the isolation of more than 1,200 natural products, mainly sesquiterpenes, diterpenes, triterpenes and C_15_ acetogenins [2,3]. Nevertheless, they are still considered a fascinating source of new natural products, usually halogenated, that exhibit a variety of biological activities, including cytotoxic [4,5], antimicrobial [6], antiviral [7], antiparasitic [8], anti-inflammatory [9] and antifouling [10].

**Figure 1:**
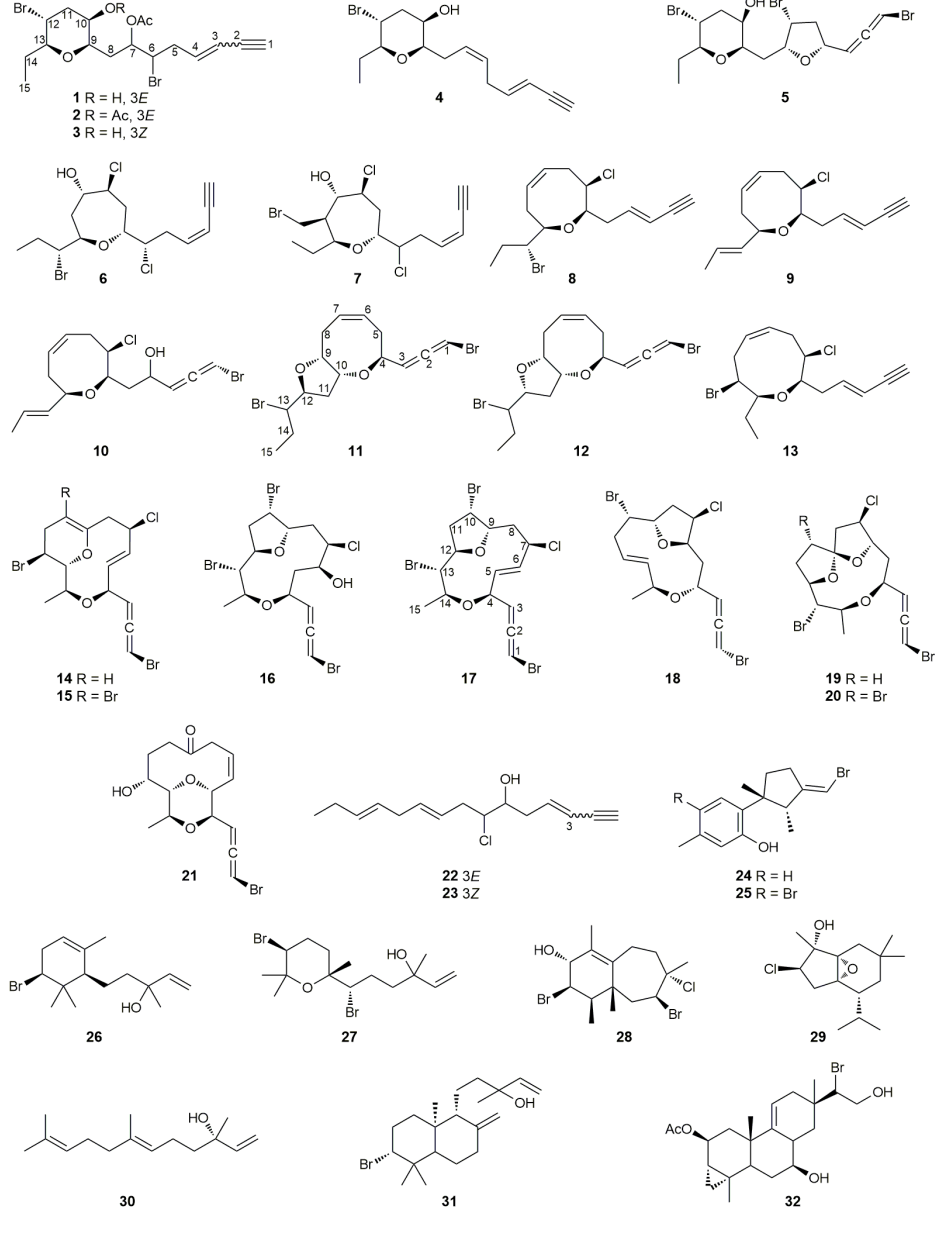
Chemical structures of compounds **1**–**32** isolated from the red algae *Laurencia microcladia* (**1**–**7**, **11**–**12**, **24**–**27**, and **29**–**32**) and *Laurencia obtusa* (**7**–**10**, **13**–**25**, and **28**).

In the framework of our ongoing research on new bioactive natural products from the genus *Laurencia*, we investigated the chemical profiles of *Laurencia microcladia* Kützing and *Laurencia obtusa* (Hudson) Lamouroux, both collected off the coasts of Tinos Island in the Aegean Sea. Herein, we report the isolation and structure elucidation of 32 compounds (**1**–**32**) (Figure 1), among which six (**2**, **3**, **4**, **11**, **12** and **17**) are new natural products. In addition, we report the evaluation of the antibacterial activities of compounds **1**–**3**, **5**–**11**, **13** and **15**–**32** against *Staphylococcus aureus* and *Escherichia coli*.

## 2. Results and Discussion

A series of chromatographic separations of the organic extract of *L. microcladia* resulted in the isolation of 17 compounds (**1**–**7**, **11**–**12**, **24**–**27** and **29**–**32**), including nine C_15_ acetogenins (**1**–**7** and **11**–**12**), among which five are new natural products (**2**, **3**, **4**, **11** and **12**), six sesquiterpenes (**24**–**27**, **29** and **30**) and two diterpenes (**31** and **32**). In a similar manner, the organic extract of *L. obtusa* was subjected to repetitive chromatographic fractionations to yield 18 metabolites (**7**–**10**, **13**–**25** and **28**), including fifteen C_15_ acetogenins (**7**–**10** and **13**–**23**), among which one is a new natural product (**17**), and three sesquiterpenes (**24**, **25** and **28**). The previously reported metabolites were identified as sagonenyne (**1**) [11], the bicyclic C_15_ acetogenin **5** [12], rogioloxepane C (**6**) [13], rogiolenyne B (**7**) [14,15], *trans*-pinnatifidenyne (**8**) [16,17], (3*E*)-laurenyne (**9**) [18,19], marilzallene B (**10**) [20], (3*E*,6*R*,7*R*)-obtusenyne (**13**) [21], obtusallene I (**14**) [22,23], 10-bromo-obtusallene I (**15**) [24], obtusallene VII (**16**) [25,26], obtusallene IV (**18**) [27,28], obtusallene VI (**19**) [25,26], obtusallene V (**20**) [25,26], chondrioallene (**21**) [20], the linear acetogenins **22** and **23** [17], *iso*-laurenisol (**24**) [29], bromolaurenisol (**25**) [30], *α*-snyderol (**26**) [31], obtusenol (**27**) [32,33], perforenol (**28**) [34], the brasilane derivative **29** [35], *trans*-nerolidol (**30**) [36,37], isopinnatol B (**31**) [38] and deoxyparguerol (**32**) [39] by comparison of their spectroscopic and physical characteristics with those reported in the literature. Compounds **22** and **23** have been previously reported only as semisynthetic products and are isolated for the first time from a natural source.

**Figure 2:**
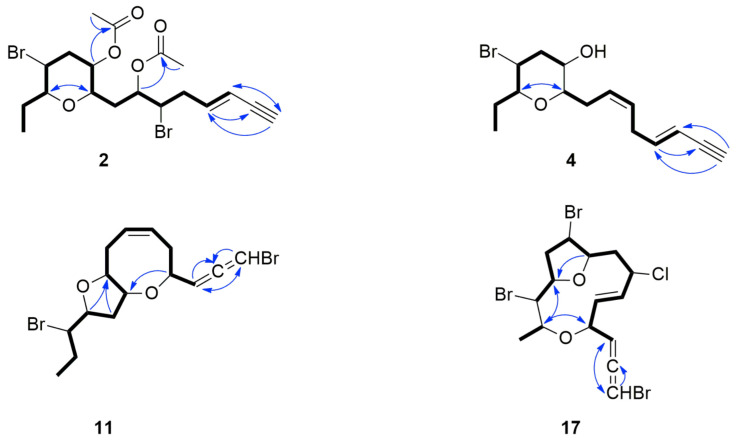
COSY (bold bonds) and important HMBC (arrows) correlations observed for compounds **2**, **4**, **11** and **17**.

**Figure 3:**
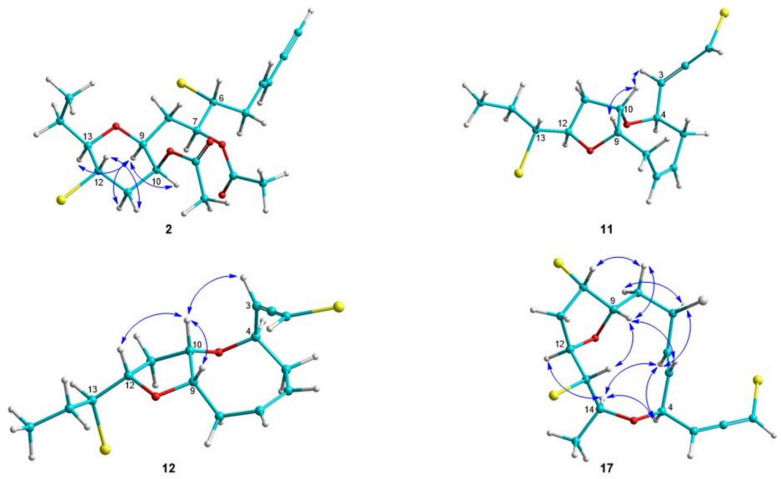
Key NOE correlations observed for compounds **2**, **11**, **12** and **17**.

Compound **2** was obtained as a colorless oil. Based on HR-APCIMS and NMR data, its molecular formula was calculated as C_19_H_26_Br_2_O_5_. The presence of three quaternary, nine methine, four methylene and three methyl carbon atoms was deduced by the HSQC and HMBC spectra (Table 1 and Table 2). In addition, the chemical shift of the quaternary carbon at *δ*_C_ 81.5, along with the resonances of three tertiary carbons at *δ*_C_ 77.3, 112.2 and 141.2, were indicative of a terminal enyne moiety. Furthermore, the presence of two acetoxy groups was evident. The remaining signals in the ^1^H NMR spectrum of **2** were reminiscent of C_15_ acetogenins, frequently isolated from species of *Laurencia*. Indeed, analysis of the HSQC, HMBC and COSY spectra revealed that **2** featured a tetrahydropyran ring resulting from the formation of an ether bridge between C-9 and C-13, with the two acetoxy groups positioned at C-7 and C-10 (Figure 2). The geometry of the Δ^3^ double bond was determined as *E* on the basis of the coupling constant value (*J* = 15.8 Hz) measured between the olefinic methines H-3 and H-4, as well as the chemical shift of the acetylenic proton H-1 (*δ*_H_ 2.83). The assignment of the relative configuration of the asymmetric centers of **2** relied on the measured coupling constants and observed NOE cross-peaks (Figure 3). Specifically, the NOE interactions of H-9/H-13, H-9/H-11α and H-11β/H-12, along with the coupling constant of H-12/H-13 (*J* = 10.0 Hz), established the axial orientation of H-9, H-11α, H-12 and H-13 and the relative configurations at C-9, C-12 and C-13. The equatorial orientation of H-10 and the relative configuration at C-10 were deduced based on the small coupling constants of H-10, which appeared as a broad singlet peak, and the NOE enhancement of H-9/H-10. Thus, the relative configuration of the stereogenic centers of **2** was established as 9R*,10R*,12R*,13S*, and as such compound **2** was identified as 10-acetyl-sagonenyne.

Compound **3**, which exhibited a pseudomolecular ion peak at *m*/*z* 451.0111 with isotopic ion peaks at *m*/*z* 453.0092 and 455.0070 (HR-APCIMS) corresponding to C_17_H_25_Br_2_O_4_, was isolated as a colorless oil. The NMR spectroscopic features (Table 1 and Table 2), pointing to a C_15_ acetogenin bearing an acetoxy group, closely resembled those of metabolite **1**. Analysis of the 2D NMR spectra of **3** suggested that it was the geometric isomer of **1**. Indeed, the chemical shift of the acetylenic proton H-1 (*δ*_H_ 3.12) and the coupling constant value measured between the olefinic methines H-3 and H-4 (*J* = 10.9 Hz) assigned the geometry of the double bond of the terminal -enyne system of **3** as *Z*, while the remaining NOE enhancements and coupling constants observed were in accordance with those of compound **1**. Therefore, compound **3** was identified as *cis*-sagonenyne.

Compound **4** was obtained as a colorless oil. Its molecular formula was deduced as C_15_H_21_Br_2_O on the basis of its HR-APCIMS and NMR data. Comparison of its ^1^H and ^13^C NMR data (Table 1 and Table 2) with those of thuwalenyne C [40] revealed that the main difference was the geometry of the Δ^3^ double bond, which was determined as *E*, as suggested by the coupling constant measured between H-3 and H-4 (*J* = 16.0 Hz) and the chemical shift of the acetylenic proton H-1 (*δ*_H_ 2.79). The geometry of the Δ^6^ double bond was assigned as *Z* on the basis of the resonance of the doubly allylic methylene carbon C-5 at *δ*_C_ 30.7 [41]. Even though compound **4** proved unstable before the acquisition of NOESY spectrum, the high similarity observed in the chemical shifts and coupling constants of H-9 (3.44 ddd (6.8, 6.8, 0.8) vs. 3.45 ddd (7.3, 7.3, 0.6)), H-10 (3.67 brs vs. 3.68 brs), H-12 (3.98 ddd (12.5, 10.3, 4.7) vs. 3.98 ddd (12.3, 9.9, 4.5)) and H-13 (3.33 ddd (10.3, 9.0, 2.3) vs. 3.33 ddd (9.9, 9.9, 2.1)) of **4** in comparison to those of thuwalenyne C [40] unambiguously suggested the same relative configuration. Therefore, compound **4** was identified as *trans*-thuwalenyne C.

Tinosallene A (**11**), isolated as a yellow oil, displayed the molecular formula C_15_H_20_Br_2_O_2_, as indicated by the HR-APCIMS and NMR data. The HSQC and HMBC spectra indicated the presence of 15 carbons, including one methyl, four methylenes, nine methines and one non-protonated carbon (Table 1 and Table 2). The signals of a bromoallene moiety (*δ*_H/C_ 6.09/74.5, 202.9 and 5.39/100.2) and two olefinic methines (*δ*_H/C_ 5.82/129.9 and 5.85/128.0), as well as of five deshielded carbons bearing halogen or oxygen atoms (*δ*_C_ 63.2, 75.4, 75.4, 80.8 and 85.4) were evident. Since the allene moiety and the isolated double bond accounted for three of the five degrees of unsaturation, the molecular structure of **11** was determined as bicyclic. The NMR spectroscopic features of **11** (Table 1 and Table 2) resembled those of thuwalallene E [40]. Specifically, the COSY correlations identified an extended spin system spanning from C-3 to C-15, while the HMBC correlations from H-4 to C-10 and from H-12 to C-9 identified the oxocane and tetrahydrofuran rings, respectively (Figure 2). The NOE cross-peaks of H-3/H-10 and H-9/H-10, in conjunction with the simultaneous absence of NOE correlations between H-4 and H-9, as well as between H-10 and H-12, determined the relative configurations at C-4, C-9, C-10 and C-12 as 4R*,9S*,10S*,12R* (Figure 3). The absolute configuration of the bromoallene functionality was established as 2*S* on the basis of the positive value of its measured optical rotation, according to the empirical rule proposed by Lowe about the absolute configuration of chiral allenes [42,43].

Tinosallene B (**12**), obtained as a yellow oil, had the same molecular formula (C_15_H_20_Br_2_O_2_) as **11**, as established from its HR-APCIMS and NMR data. In addition, the ^1^H NMR data of **12** shared a high degree of similarity to those of compound **11**, with the exception of H-9, H-11 and H-12 (Table 1 and Table 2). Analysis of the 2D NMR spectra of **12**, which proved to be unstable, indicated the same planar structure as that of tinosallene A (**11**). However, the correlations of H-3/H-10, H-9/H-10 and H-10/H-12 observed in the NOESY spectrum of **12** established the relative configurations at C-4, C-9, C-10 and C-12 as 4R*,9S*,10S*,12S* (Figure 3), thereby identifying **12** as the epimer of **11** at C-12. Moreover, the positive value of the optical rotation measured for compound **12** was indicative of the 2*S* configuration of the bromoallene moiety.

Obtusallene XI (**17**), obtained as a white solid, displayed the molecular formula C_15_H_18_Br_3_ClO_2_, as indicated by its HR-APCIMS measurements. The presence of one non-protonated, eleven methine, two methylene and one methyl carbon atoms was confirmed by the HSQC and HMBC experiments (Table 1 and Table 2). Further interpretation of its NMR data revealed a bromoallene moiety (*δ*_H/C_ 6.05/74.2, 202.4, 5.52/100.9), a 1,2-disubstituted double bond (*δ*_H/C_ 5.88/128.7 and 5.91/134.9) and seven deshielded methines (*δ*_H/C_ 3.64/75.0, 4.03/63.8, 4.09/77.3, 4.27/75.2, 4.44/53.7, 4.52/78.8 and 4.86/57.6). Taking into account the bromoallene functionality and the isolated double bond as three of the five degrees of unsaturation, metabolite **17** was determined as bicyclic. The COSY cross-peaks readily identified two spin systems spanning from C-3 to C-13 and from C-14 to C-15. The HMBC correlations of H-1 with both C-2 and C-3, as well as of both H-4 and H-12 with C-14, completed the C_15_ acetogenin chain and established the 4,14-epoxy system. Furthermore, the HMBC correlation of H-9 with C-12 suggested the formation of a second ether bridge between C-9 and C-12. The NOE interactions of H-4/H-6, H-4/H-14, H-6/H-7, H-7/H-8α and H-12/H-14 confirmed that H-4, H-6, H-7, H-8α, H-12 and H-14 were on the same side of the ring plane and orientated downwards, while the NOE cross-peaks of H-5/H-13, H-8β/H-9, H-8β/H-10 and H-9/H-13 suggested that H-5, H-8β, H-9, H-10 and H-13 were on the opposite side of the ring plane and oriented upwards (Figure 3). The geometry of the Δ^5^ double bond was assigned as *E* due to the measured coupling constant in C_6_D_6_ (*J* = 14.7 Hz). On this basis, the relative configurations of the chiral centers of **17** were determined as 4S*,7R*,9S*,10S*,12R*,13S*,14S*, while the strong negative value of the optical rotation measured indicated the absolute configuration of the bromoallene moiety as 2*R*.

Compounds **1**–**3**, **5**–**11**, **13** and **15**–**32** were evaluated for their antibacterial activity against a Gram-positive (*S. aureus* strain ATCC-25923) and a Gram-negative (*E. coli* strain NCTC-10418) bacterial strain (Table S1). Among them, *iso*-laurenisol (**24**) and bromolaurenisol (**25**) were active against *S. aureus*, with minimum inhibition concentrations (MICs) of 16 and 4 μg/mL, respectively, while rogiolenyne B (**7**) was mildly active against the same strain, with a MIC value of 128 μg/mL. In contrast, all of the compounds tested were proven inactive against *E. coli*.

## 3. Materials and Methods

### 3.1. General Experimental Procedures

Optical rotations were measured on a Krüss model P3000 polarimeter (A. KRÜSS Optronic GmbH, Hamburg, Germany) with a 0.5 dm cell. UV spectra were obtained on a Perkin Elmer Lambda 40 spectrophotometer (PerkinElmer Ltd., Buckinghamshire, UK). IR spectra were obtained on a Bruker Alpha II spectrometer (Bruker Optik GmbH, Ettlingen, Germany). The 1D and 2D NMR spectra were recorded on Bruker AC 200, DRX 400, Avance III 600 and Avance NEO 700 (Bruker BioSpin GmbH, Rheinstetten, Germany) spectrometers, using standard Bruker pulse sequences at room temperature. Chemical shifts are given on a *δ* (ppm) scale using TMS as the internal standard. High-resolution APCI mass spectra were measured on a Thermo Scientific LTQ Orbitrap Velos mass spectrometer (Thermo Fisher Scientific, Bremen, Germany). Low-resolution EI mass spectra were measured on a Hewlett-Packard 5973 mass spectrometer (Agilent Technologies, Santa Clara, CA, USA). Low-resolution CI mass spectra were measured on a Thermo Electron Corporation DSQ mass spectrometer (Thermo Electron Corporation, Austin, TX, USA) using a direct-exposure probe with CH_4_ as the reagent gas. Normal- and reversed-phase column chromatography separations were performed with Kieselgel Si 60 (Merck, Darmstadt, Germany) and Kieselgel RP-18 (Merck, Darmstadt, Germany), respectively. HPLC separations were conducted on a Pharmacia LKB 2248 liquid chromatograph (Pharmacia LKB Biotechnology, Uppsala, Sweden) equipped with an RI-102 Shodex refractive index detector (ECOM spol. s r.o., Prague, Czech Republic), using the following columns: (i) Econosphere Silica 10u (250 × 10 mm, Grace, Columbia, MD, USA) or (ii) Chiralcel OD 10 μm (250 × 10 mm, Daicel Chemical Industries Ltd., Osaka, Japan). TLC was performed with Kieselgel 60 F_254_ aluminum-backed plates (Merck, Darmstadt, Germany) and spots were visualized after spraying with 15% (*v*/*v*) H_2_SO_4_ in MeOH reagent and heating at 100 °C for 1 min.

### 3.2. Biological Material

Specimens of *Laurencia microcladia* Kützing and *Laurencia obtusa* (Hudson) Lamouroux were collected from the coasts of Agia Kyriaki and Agios Sostis, respectively, at Tinos Island in the Aegean Sea, at a depth of 0.5–2 m in September 2011. Voucher specimens of the algae have been deposited at the Herbarium of the Section of Pharmacognosy and Chemistry of Natural Products, Department of Pharmacy, National and Kapodistrian University of Athens (ATPH/MP0290 and ATPH/MP0291, respectively).

### 3.3. Extraction and Isolation

Fresh algal specimens of *L. microcladia* were exhaustively extracted with mixtures of CH_2_Cl_2_ and MeOH (initially with MeOH 100%, followed by CH_2_Cl_2_/MeOH 1:1 and finally with CH_2_Cl_2_ 100%) at room temperature. After evaporation of the solvent of the combined extracts in vacuo, the organic extract (13.2 g) was fractionated by vacuum column chromatography on silica gel, using cHex with increasing amounts of EtOAc followed by EtOAc with increasing amounts of MeOH as the mobile phase to afford 12 fractions (Lmt1–Lmt12). Fractions Lmt4 and Lmt5 (15–30% EtOAc in cHex, 1.67 g) were combined and further separated by gravity column chromatography on silica gel, using mixtures of cHex and EtOAc of increasing polarity as eluent to yield 23 fractions (Lmt4a-Lmt4w), among which **1** (54.5 mg), **27** (717.0 mg) and **30** (88.4 mg) were isolated in pure form. Fractions Lmt4f and Lmt4g (5–6% EtOAc in cHex, 230.3 mg) were combined and fractionated by gravity column chromatography on silica gel, using mixtures of cHex and EtOAc of increasing polarity as eluent to afford 18 fractions (Lmt4f1-Lmt4f18), among which **24** (100.6 mg) was isolated in pure form. Fraction Lmt4f10 (1% EtOAc in cHex, 21.1 mg) was subjected to normal-phase HPLC, eluting with cHex/EtOAc (98:2) and subsequently *n*-hexane/EtOAc (98:2), to yield **12** (1.3 mg). Fraction Lmt4f15 (5% EtOAc in cHex, 26.9 mg) was submitted to normal-phase HPLC, using cHex/EtOAc (90:10) as the eluent, and subsequently to chiral HPLC, eluting with *n*-hexane/i-PrOH (95:5), to afford **11** (4.0 mg) and **24** (3.8 mg). In a similar manner, fractions Lmt4h (6% EtOAc in cHex, 16.9 mg), Lmt4l (7% EtOAc in cHex, 121.2 mg) and Lmt4p (12% EtOAc in cHex, 24.9 mg) were separately purified by normal-phase HPLC, using cHex/EtOAc (93:7) as the eluent, and subsequently chiral HPLC, using *n*-hexane/i-PrOH (95:5) as the eluent, to yield **2** (5.1 mg), **4** (1.1 mg), **7** (0.9 mg), **11** (3.0 mg), **24** (4.9 mg), **25** (6.5 mg), **26** (11.8 mg), **27** (99.6 mg), **29** (0.6 mg), **30** (3.8 mg) and **31** (3.0 mg). Fraction Lmt4t (15% EtOAc in cHex, 22.0 mg) was subjected to normal-phase HPLC, eluting with cHex/EtOAc (80:20), and subsequently chiral HPLC, using *n*-hexane/i-PrOH (90:10) as the eluent, to afford **5** (6.2 mg) and **6** (2.2 mg). Fractions Lmt4v (25–50% EtOAc in cHex, 20.7 mg) and Lmt6 (30–35% EtOAc in cHex, 119.8 mg) were combined and further separated by gravity column chromatography on silica gel, using mixtures of cHex and EtOAc of increasing polarity as the mobile phase to yield 13 fractions (Lmt6a-Lmt6m), among which **1** (41.3 mg) and **27** (5.4 mg) were isolated in pure form. Fraction Lmt6i (18% EtOAc in cHex, 10.0 mg) was submitted to normal-phase HPLC, eluting with cHex/EtOAc (85:15), and subsequently chiral HPLC, using *n*-hexane/i-PrOH (90:10) as the eluent, to afford **1** (1.2 mg), **3** (1.0 mg) and **6** (0.4 mg). Fraction Lmt6k (20–30% EtOAc in cHex, 18.1 mg) was purified by normal-phase HPLC, eluting with cHex/EtOAc (75:25), to yield **1** (2.3 mg). Fractions Lmt9 and Lmt10 (50–100% EtOAc in cHex, 320.0 mg) were combined and further fractionated by gravity column chromatography on silica gel, using cHex with increasing amounts of EtOAc followed by EtOAc with increasing amounts of MeOH as the mobile phase to afford 13 fractions (Lmt9a-Lmt9m). Fraction Lmt9i (60% EtOAc in cHex, 25.9 mg) was purified by normal-phase HPLC, using cHex/EtOAc (40:60) as the eluent, to yield **32** (1.2 mg).

Specimens of the fresh alga *L. obtusa* were exhaustively extracted with mixtures of CH_2_Cl_2_ and MeOH (initially with MeOH 100%, followed by CH_2_Cl_2_/MeOH 1:1 and finally with CH_2_Cl_2_ 100%) at room temperature. Evaporation of the solvent of the combined extracts in vacuo afforded a residue (6.6 g) that was subjected to vacuum column chromatography on silica gel, using cHex with increasing amounts of EtOAc followed by EtOAc with increasing amounts of MeOH as the mobile phase to afford 13 fractions (Lot1–Lot13). Fractions Lot4 (20% EtOAc in cHex, 257.0 mg) and Lot5a (5–7% EtOAc in cHex, 56.6 mg) were combined and further separated by gravity column chromatography on silica gel, using mixtures of cHex and EtOAc of increasing polarity as eluent to yield 10 fractions (Lot4a-Lot4j). Fraction Lot4c (3% EtOAc in cHex, 91.2 mg) was fractionated by gravity column chromatography on silica gel, using mixtures of cHex and EtOAc of increasing polarity as eluent to afford 6 fractions (Lot4c1-Lot4c6). Fractions Lot4c2 (2% EtOAc in cHex, 52.0 mg), Lot4c3 (2% EtOAc in cHex, 32.2 mg) and Lot4c4 (2% EtOAc in cHex, 4.0 mg) were separately submitted to normal-phase HPLC, using cHex/EtOAc (99:1) and subsequently *n*-hexane/EtOAc (99:1) as the eluents, to yield **8** (2.5 mg), **9** (24.6 mg) and **13** (0.9 mg). Fractions Lot4d (3% EtOAc in cHex, 14.2 mg) and Lot4e (3% EtOAc in cHex, 13.0 mg) were separately purified by normal-phase HPLC, eluting with cHex/EtOAc (98:2) and then *n*-hexane/EtOAc (98:2), and subsequently chiral HPLC, eluting with *n*-hexane/i-PrOH (99:1), to afford **13** (4.1 mg), **17** (4.7 mg), **19** (6.1 mg) and **20** (4.1 mg). In a similar manner, fraction Lot4g (10% EtOAc in cHex, 74.2 mg) was subjected to normal-phase HPLC, using cHex/EtOAc (90:10) and then *n*-hexane/EtOAc (90:10) as the eluents, and subsequently chiral HPLC, using *n*-hexane/i-PrOH (95:5) as the eluent, to yield **18** (3.7 mg), **22** (1.7 mg), **23** (3.0 mg), **24** (8.6 mg), **25** (14.5 mg) and **28** (2.4 mg). Fraction Lot5 (30% EtOAc in cHex, 127.8 mg) was fractionated by gravity column chromatography on silica gel, using mixtures of cHex and EtOAc of increasing polarity as the mobile phase to afford 6 fractions (Lot5a–Lot5f). Fractions Lot5b (7–10% EtOAc in cHex, 25.2 mg) and Lot5c (10% EtOAc in cHex, 24.1 mg) were separately purified by normal-phase HPLC, eluting with cHex/EtOAc (90:10) and subsequently cHex/Me_2_CO (93:7), to yield **7** (0.9 mg), **14** (17.2 mg) and **15** (8.7 mg). Fraction Lot5d (10–20% EtOAc in cHex, 17.2 mg) was submitted to normal-phase HPLC, using cHex/EtOAc (80:20) as the eluent, to afford **10** (4.2 mg). Fraction Lot6 (30% EtOAc in cHex, 120.4 mg) was fractionated by gravity column chromatography on silica gel, using cHex with increasing amounts of EtOAc as the mobile phase to yield 12 fractions (Lot6a–Lot6l). Fraction Lot6h (17–30% EtOAc in cHex, 7.4 mg) was purified by normal-phase HPLC, using cHex/EtOAc (80:20) as eluent, to afford **16** (2.6 mg). Fraction Lot12 (0–25% MeOH in EtOAc, 49.3 mg) was subjected to vacuum liquid chromatography on C-18 silica gel eluting with mixtures of MeOH and H_2_O of decreasing polarity to yield **21** (25.6 mg).

10-Acetyl-sagonenyne (**2**): colorless oil; $[\alpha {]}_{D}^{20}$ −6.0 (*c* 0.03, CHCl_3_); UV (CH_2_Cl_2_) *λ*_max_ (log *ε*) 232 (4.74) nm; IR (thin film) *ν*_max_ 3290, 2966, 2932, 2876, 2855, 1742, 1371, 1227, 1030 cm^−1^; ^13^C NMR data, see Table 1; ^1^H NMR data, see Table 2; HR-APCIMS *m*/*z* 493.0220, 495.0198, 497.0177 [M + H]^+^ (49:100:51) (calcd. for C_19_H_27_^79^Br_2_O_5_, 493.0220, C_19_H_27_^79^Br^81^BrO_5_, 495.0199, C_19_H_27_^81^Br_2_O_5_, 497.0179).

*cis*-Sagonenyne (**3**): colorless oil; $[\alpha {]}_{D}^{20}$ −29.9 (*c* 0.07, CHCl_3_); UV (CH_2_Cl_2_) *λ*_max_ (log *ε*) 232 (4.71) nm; IR (thin film) *ν*_max_ 3448, 3294, 2970, 2927, 2880, 2859, 1742, 1371, 1231, 1082, 1047, 1022 cm^−1^; ^13^C NMR data, see Table 1; ^1^H NMR data, see Table 2; HR-APCIMS *m*/*z* 451.0111, 453.0092, 455.0070 [M + H]^+^ (52:100:48) (calcd. for C_17_H_25_^79^Br_2_O_4_, 451.0114, C_17_H_25_^79^Br^81^BrO_4_, 453.0094, C_17_H_25_^81^Br_2_O_4_, 455.0073).

*trans*-Thuwalenyne C (**4**): colorless oil; $[\alpha {]}_{D}^{20}$ −13.7 (*c* 0.21, CHCl_3_); UV (CHCl_3_) *λ*_max_ (log *ε*) 240 (3.48) nm; IR (thin film) *ν*_max_ 3286, 2925, 1719, 1078 cm^−1^; ^13^C NMR data, see Table 1; ^1^H NMR data, see Table 2; HR-APCIMS *m*/*z* 311.0632, 313.0610 [M − H]^−^ (100:98) (calcd. for C_15_H_20_^79^BrO_2_, 311.0652, C_15_H_20_^81^BrO_2_, 313.0632).

Tinosallene A (**11**): yellow oil; $[\alpha {]}_{D}^{20}$ +15.1 (*c* 0.53, CHCl_3_); UV (CH_2_Cl_2_) *λ*_max_ (log *ε*) 230 (3.43) nm; IR (thin film) *ν*_max_ 3030, 2966, 2936, 2881, 2855, 1725, 1056, 745, 660 cm^−1^; ^13^C NMR data, see Table 1; ^1^H NMR data, see Table 2; HR-APCIMS *m*/*z* 390.9898, 392.9879, 394.9858 [M + H]^+^ (51:100:49) (calcd. for C_15_H_21_^79^Br_2_O_2_, 390.9903, C_15_H_21_^79^Br^81^BrO_2_, 392.9882, C_15_H_21_^81^Br_2_O_2_, 394.9862).

Tinosallene B (**12**): yellow oil; $[\alpha {]}_{D}^{20}$ +14.7 (*c* 0.46, CHCl_3_); UV (CH_2_Cl_2_) *λ*_max_ (log *ε*) 229 (3.35) nm; IR (thin film) *ν*_max_ 3029, 2967, 2936, 2879, 2854, 1727, 1054, 745, 661 cm^−1^; ^13^C NMR data, see Table 1; ^1^H NMR data, see Table 2; HR-APCIMS *m*/*z* 390.9899, 392.9880, 394.9859 [M + H]^+^ (51:100:49) (calcd. for C_15_H_21_^79^Br_2_O_2_, 390.9903, C_15_H_21_^79^Br^81^BrO_2_, 392.9882, C_15_H_21_^81^Br_2_O_2_, 394.9862).

Obtusallene XI (**17**): white solid; $[\alpha {]}_{D}^{20}$ −74.0 (*c* 0.35, CHCl_3_); UV (CHCl_3_) *λ*_max_ (log *ε*) 232 (3.18) nm; IR (thin film) *ν*_max_ 3060, 2927, 2855, 1077, 1030, 967, 860 cm^−1^; ^13^C NMR data, see Table 1; ^1^H NMR data in CDCl_3_, see Table 2; ^1^H NMR (C_6_D_6_, 400 MHz, *δ* in ppm, *J* in Hz): 5.74 (dd, *J* = 14.7, 2.0, H-5), 5.71 (dd, *J* = 5.7, 2.0, H-1), 5.28 (t, *J* = 5.7, H-3), 5.17 (dd, *J* = 14.7, 3.5, H-6), 4.12 (m, H-7), 4.08 (ddd, *J* = 14.7, 5.7, 2.0, H-4), 3.90 (q, *J* = 6.5, H-14), 3.66 (m, H-13), 3.64 (m, H-10 and H-12), 3.13 (ddd, *J* = 7.7, 2.4, 0.8, H-9), 2.19 (ddd, *J* = 15.4, 7.4, 7.4, H-11a), 1.90 (m, H-11b), 1.82 (m, H-8*α*), 1.71 (ddd, *J* = 15.4, 7.7, 4.0, H-8*β*), 1.13 (d, *J* = 6.5, H_3_-15), HR-APCIMS *m*/*z* 502.8615, 504.8593, 506.8570, 508.8546, 510.8518 [M + H]^+^ (34:95:100:42:12) (calcd. for C_15_H_19_^79^Br_3_^35^ClO_2_, 502.8618, C_15_H_19_^79^Br_2_^81^Br^35^ClO_2_, 504.8598, C_15_H_19_^79^Br^81^Br_2_^35^ClO_2_, 506.8577, C_15_H_19_^81^Br_3_^35^ClO_2_, 508.8557, C_15_H_19_^81^Br_3_^37^ClO_2_, 510.8527).

### 3.4. Evaluation of Antibacterial Activity

The bacterial strains *S. aureus* ATCC-25923 and *E. coli* NCTC-10418 were generously provided by Prof. S. Gibbons (University of East Anglia). The antibacterial activity evaluation was performed as previously described [44]. Briefly, both strains were cultured on nutrient agar and incubated at 37 °C for 24 h prior to the determination of MIC values. Compounds **1**–**3**, **5**–**11**, **13** and **15**–**32** were dissolved in DMSO and subsequently diluted in Mueller−Hinton broth (MHB) to give an initial concentration of 512 μg/mL. For each strain, bacterial inocula equivalent to the 0.5 McFarland turbidity standard were prepared in normal saline and diluted to a final inoculum density of 5 × 10^5^ cfu/mL. MHB supplemented with 10 mg/L Mg^2+^ and 20 mg/L Ca^2+^ (125 μL/well) was dispensed into wells 1−11 of each row of 96-well microtiter plates. The compound solution (125 μL) was added to the first well of each row and serially diluted across the row, leaving well 11 empty for growth control. The final volume was dispensed into well 12, which being free of MHB or inoculum served as the sterility control. The inoculum (125 μL/well) was added to wells 1−11 of each row, and the microtiter plates were incubated at 37 °C for 18 h. The lowest concentration at which no bacterial growth was observed was recorded as the MIC. The observation was confirmed by the addition of a 5 mg/mL methanolic solution of 3-(4,5-dimethylthiazol-2-yl)-2,5-diphenyltetrazolium bromide (MTT, 20 μL/well) and further incubation at 37 °C for 20 min. Bacterial growth was indicated by a color alteration from yellow to dark blue. Vancomycin and chloramphenicol were used as positive controls for *S. aureus* and *E. coli* strains, respectively. The highest concentration of DMSO remaining after dilution (3.125% *v*/*v*) caused no inhibition of bacterial growth. All samples were tested in triplicate.

## 4. Conclusions

The chemical investigations of the organic extracts of *L. microcladia* and *L. obtusa* collected off the coasts of Tinos Island in the Aegean Sea led to the isolation and structure elucidation of six new (**2**–**4**, **11**, **12** and **17**) and 26 known (**1**, **5**–**10**, **13**–**16** and **18**–**32**) secondary metabolites. Among the new compounds, **2**, **3** and **4** possessing a 9,13-epoxy ring belong to the small group of tetrahydropyran C_15_ acetogenins, which includes only seven more representatives to date. Tinosallenes A (**11**) and B (**12**) are stereoisomers of thuwalallene E, with these three being the only C_15_ acetogenins bearing a 4,10:9,12-bisepoxy system. Obtusallene XI (**17**) belongs to the obtusallene family, a rare class of twelve-membered ring C_15_ acetogenins, consisting of approximately 25 representatives characterized by a 4,14-epoxy core and a bromoallene terminal functionality, while incorporating one or two more ether rings into their structures. *Iso*-laurenisol (**24**) and bromolaurenisol (**25**) were active against *S. aureus*, while all metabolites tested were proven inactive against *E. coli*.

## Figures and Tables

**Table 1:** ^13^C NMR (*δ* in ppm) data of compounds **2**, **3**, **4**, **11**, **12** and **17** recorded at 50.3 MHz in CDCl_3_.

Position	2	3	4	11	12	17
1	77.3	82.8	77.2	74.5	74.4	74.2
2	81.5	79.7	81.5 ^1^	202.9	202.8 ^1^	202.4 ^1^
3	112.2	111.3	109.0	100.2	100.4	100.9
4	141.2	140.7	144.1	75.4	75.1	78.8
5	38.8	35.6	30.7	31.6	31.5	128.7
6	55.3	55.3	127.8	128.0	127.4	134.9
7	71.2	71.7	126.9	129.9	130.0	57.6
8	35.1	35.0	29.3	28.7	29.3	39.2
9	74.8	77.2	79.7	85.4	85.5	75.0
10	71.3	70.0	68.9	75.4	74.2	53.7
11	40.4	43.4	43.4	38.7	39.3	43.0
12	47.4	47.5	47.8	80.8	80.9	77.3
13	83.6	84.0	83.9	63.2	61.9	63.8
14	26.3	26.4	26.3	28.5	28.2	75.2
15	9.7	9.6	9.5	12.1	11.6	19.7
16	170.1	170.1				
17	20.9	20.9				
18	170.4					
19	21.1					

^1^ Chemical shifts were determined through HMBC correlations.

**Table 2:** ^1^H NMR (*δ* in ppm, *J* in Hz) data of compounds **2**, **3**, **4**, **11**, **12** and **17** recorded at 400 MHz in CDCl_3_.

Position	2	3	4	11	12	17
1	2.83 d (1.6)	3.12 d (1.7)	2.79 d (2.0)	6.09 dd (5.6, 3.2)	6.08 dd (5.4, 3.1)	6.05 dd (5.8, 1.8)
3	5.53 dd (15.8, 1.6)	5.59 d (10.9, 1.7)	5.50 m	5.39 ddd (5.6, 4.3, 0.8)	5.38 brt (5.4)	5.52 dd (5.8, 5.8)
4	6.19 ddd (15.8, 7.3, 6.5)	6.06 ddd (10.9, 7.0, 7.0)	6.21 ddd (16.0, 6.4, 6.4)	4.85 m	4.78 m	4.52 ddd (8.6, 5.8, 1.8)
5	2.66 ddd (15.7, 6.5, 5.5), 2.52 dd (15.7, 7.3)	2.93 ddd (15.1, 7.0, 5.3), 2.79 ddd (15.1, 9.0, 7.0)	2.88 t (6.4)	2.71 dt (14.9, 4.7), 2.26 m	2.63 dt (15.1, 4.5), 2.27 m	5.88 m
6	4.01 ddd (8.8, 5.5, 2.5)	4.12 ddd (9.0, 5.3, 3.1)	5.50 m	5.85 m	5.79 m	5.91 m
7	5.21 ddd (8.8, 2.5, 2.7)	5.21 ddd (9.0, 3.1, 3.1)	5.50 m	5.82 m	5.79 m	4.86 m
8	1.88 ddd (13.6, 10.5, 2.7), 1.72 m	2.10 m, 1.77 ddd (14.7, 9.0, 2.0)	2.29 t (6.8)	2.55 m, 2.28 m	2.57 td (12.0, 6.9), 2.25 m	2.26 dd (15.5, 2.0), 2.17 ddd (15.5, 7.5, 3.9)
9	3.50 d (10.5)	3.45 dd (10.5, 2.0)	3.44 ddd (6.8, 6.8, 0.8)	4.07 ddd (11.5, 4.7, 2.8)	3.90 dt (12.0, 3.6)	3.64 dd (7.5, 2.0)
10	4.80 brs	3.63 brs	3.67 brs	4.24 dd (5.8, 2.8)	4.19 dt (6.7, 3.6)	4.44 m
11	β 2.56 m, α 2.15 m	β 2.57 ddd (13.7, 4.6, 3.4), α 2.10 m	β 2.57 ddd (13.7, 4.7, 3.4), α 2.06 m	2.01 m	2.35 dt (14.2, 6.7), 1.95 ddd (14.2, 4.7, 3.6)	2.64 m
12	3.88 ddd (12.7, 10.0, 4.4)	3.96 ddd (12.3, 10.2, 4.6)	3.98 ddd (12.5, 10.3, 4.7)	4.31 m	3.98 m	4.09 m
13	3.27 ddd (10.0, 8.7, 1.6)	3.28 ddd (10.2, 9.2, 2.2)	3.33 ddd (10.3, 9.0, 2.3)	3.98 ddd (9.6, 6.6, 3.6)	3.98 m	4.03 m
14	2.07 m, 1.49 ddq (15.5, 8.7, 7.4)	2.05 m, 1.46 m	2.02 m, 1.48 m	1.96 m, 1.68 ddq (14.6, 9.6, 7.3)	2.13 dqd (14.5, 7.2, 1.9), 1.74 ddq (14.5, 7.2, 7.2)	4.27 q (6.5)
15	0.99 t (7.4)	0.98 t (7.4)	0.96 t (7.4)	1.04 t (7.3)	1.05 t (7.2)	1.26 d (6.5)
17	2.12 s	2.11 s	-	-	-	-
19	2.11 s	-	-	-	-	-
OH	-	1.88 brs	1.79 brs	-	-	-

## Data Availability

The data presented in this study are available in the present article and the supplementary material.

## Supplementary Materials

The following are available online at https://www.mdpi.com/article/10.3390/molecules27061866/s1. Table S1: Antibacterial activities of compounds **1**–**3**, **5**–**11**, **13** and **15**–**32**. Figures S1–S70: 1D and 2D NMR and MS spectra of compounds **1**–**32**.
