# Supplementary material for: New C15 Acetogenins from Two Species of Laurencia from the Aegean Sea
Source: Molecules. 2022 Mar 13;27(6):1866. doi: 10.3390/molecules27061866 (PMC8953645; doi:10.3390/molecules27061866)
Supplement: Supplementary file 1 [file molecules-27-01866-s001.zip › molecules-1597847-supplementary.pdf]

Supplementary Materials for

New C_{15} Acetogenins from Two Species of *Laurencia* from the Aegean Sea

Maria Harizani [1], Dafni-Ioanna Diakaki [1], Stamatios Perdikaris [1], Vassilios Roussis [1] and Efstathia Ioannou [1,*]

[1] Section of Pharmacognosy and Chemistry of Natural Products, Department of Pharmacy, School of Health Sciences, National and Kapodistrian University of Athens, Panepistimiopolis Zografou, Athens 15771, Greece; mariachariz@pharm.uoa.gr (M.H.); dafnid@pharm.uoa.gr (D.-I.D.); takiperd@hotmail.com (S.P.); roussis@pharm.uoa.gr (V.R.)

* Correspondence: eioannou@pharm.uoa.gr; Tel.: +30-210-727-4913

Table of Contents

Table S1. In vitro antibacterial activity of compounds **1–3**, **5–11**, **13** and **15–32**. 4

Figure S1. ^{1}H NMR spectrum ($CDCl_3$, 400 MHz) of compound **1**. 5

Figure S2. ^{1}H NMR spectrum ($CDCl_3$, 400 MHz) of compound **2**. 5

Figure S3. ^{13}C NMR spectrum ($CDCl_3$, 50.3 MHz) of compound **2**. 6

Figure S4. COSY spectrum ($CDCl_3$, 400 MHz) of compound **2**. 6

Figure S5. HSQC spectrum ($CDCl_3$, 400 MHz) of compound **2**. 7

Figure S6. HMBC spectrum ($CDCl_3$, 400 MHz) of compound **2**. 7

Figure S7. NOESY spectrum ($CDCl_3$, 400 MHz) of compound **2**. 8

Figure S8. HR-APCIMS of compound **2**. 8

Figure S9. ^{1}H NMR spectrum ($CDCl_3$, 400 MHz) of compound **3**. 9

Figure S10. ^{13}C NMR spectrum ($CDCl_3$, 50.3 MHz) of compound **3**. 9

Figure S11. COSY spectrum ($CDCl_3$, 400 MHz) of compound **3**. 10

Figure S12. HSQC spectrum ($CDCl_3$, 400 MHz) of compound **3**. 10

Figure S13. HMBC spectrum ($CDCl_3$, 400 MHz) of compound **3**. 11

Figure S14. 1D-NOE spectra ($CDCl_3$, 400 MHz) of compound **3**. 11

Figure S15. HR-APCIMS of compound **3**. 12

Figure S16. ^{1}H NMR spectrum ($CDCl_3$, 400 MHz) of compound **4**. 12

Figure S17. ^{13}C NMR spectrum ($CDCl_3$, 50.3 MHz) of compound **4**. 13

Figure S18. COSY spectrum ($CDCl_3$, 400 MHz) of compound **4**. 13

Figure S19. HSQC spectrum ($CDCl_3$, 400 MHz) of compound **4**. 14

Figure S20. HMBC spectrum ($CDCl_3$, 400 MHz) of compound **4**. 14

Figure S21. HR-APCIMS of compound **4**. 15

Figure S22. ^{1}H NMR spectrum ($CDCl_3$, 400 MHz) of compound **5**. 15

Figure S23. ^{1}H NMR spectrum ($CDCl_3$, 400 MHz) of compound **6**. 16

Figure S24. ^{1}H NMR spectrum ($CDCl_3$, 400 MHz) of compound **7**. 16

Figure S25. ^{1}H NMR spectrum ($CDCl_3$, 400 MHz) of compound **8**. 17

Figure S26. ^{1}H NMR spectrum ($CDCl_3$, 400 MHz) of compound **9**. 17
Figure S27. ^{1}H NMR spectrum ($CDCl_3$, 400 MHz) of compound **10**........ 18
Figure S28. ^{1}H NMR spectrum ($CDCl_3$, 400 MHz) of compound **11**........ 18
Figure S29. ^{13}C NMR spectrum ($CDCl_3$, 50.3 MHz) of compound **11**. 19
Figure S30. COSY spectrum ($CDCl_3$,400 MHz) of compound **11**........ 19
Figure S31. HSQC spectrum ($CDCl_3$, 400 MHz) of compound **11**. 20
Figure S32. HMBC spectrum ($CDCl_3$, 600 MHz) of compound **11**. 20
Figure S33. NOESY spectrum ($CDCl_3$, 600 MHz) of compound **11**. 21
Figure S34. HR-APCIMS of compound **11**. 21
Figure S35. ^{1}H NMR spectrum ($CDCl_3$, 400 MHz) of compound **12**........ 22
Figure S36. ^{13}C NMR spectrum ($CDCl_3$, 50.3 MHz) of compound **12**. 22
Figure S37. COSY spectrum ($CDCl_3$, 400 MHz) of compound **12**. 23
Figure S38. HSQC spectrum ($CDCl_3$, 400 MHz) of compound **12**. 23
Figure S39. HMBC spectrum ($CDCl_3$, 700 MHz) of compound **12** (partial decomposition was already observed)........ 24
Figure S40. NOESY spectrum ($CDCl_3$, 700 MHz) of compound **12** (partial decomposition was already observed)........ 24
Figure S41. HR-APCIMS of compound **12**. 25
Figure S42. ^{1}H NMR spectrum ($CDCl_3$, 400 MHz) of compound **13**........ 25
Figure S43. ^{1}H NMR spectrum ($CDCl_3$, 400 MHz) of compound **14**........ 26
Figure S44. ^{1}H NMR spectrum ($CDCl_3$, 400 MHz) of compound **15**........ 26
Figure S45. ^{1}H NMR spectrum ($CDCl_3$, 400 MHz) of compound **16**........ 27
Figure S46. ^{1}H NMR spectrum ($CDCl_3$, 400 MHz) of compound **17**........ 27
Figure S47. ^{13}C NMR spectrum ($CDCl_3$, 50.3 MHz) of compound **17**. 28
Figure S48. COSY spectrum ($CDCl_3$, 400 MHz) of compound **17**. 28
Figure S49. HSQC spectrum ($CDCl_3$, 400 MHz) of compound **17**. 29
Figure S50. HMBC spectrum ($CDCl_3$, 400 MHz) of compound **17**. 29
Figure S51. NOESY spectrum ($CDCl_3$, 400 MHz) of compound **17**. 30
Figure S52. HR-APCIMS of compound **17**. 30
Figure S53. ^{1}H NMR spectrum (C_6D_6, 400 MHz) of compound **17**. 31
Figure S54. COSY spectrum (C_6D_6, 400 MHz) of compound **17**. 31
Figure S55. 1D-NOE spectra (C_6D_6, 400 MHz) of compound **17**. 32
Figure S56. ^{1}H NMR spectrum ($CDCl_3$, 400 MHz) of compound **18**........ 32
Figure S57. ^{1}H NMR spectrum ($CDCl_3$, 400 MHz) of compound **19**........ 33
Figure S58. ^{1}H NMR spectrum ($CDCl_3$, 400 MHz) of compound **20**........ 33
Figure S59. ^{1}H NMR spectrum ($CDCl_3$, 400 MHz) of compound **21**........ 34
Figure S60. ^{1}H NMR spectrum ($CDCl_3$, 400 MHz) of compound **22**........ 34
Figure S61. ^{1}H NMR spectrum ($CDCl_3$, 400 MHz) of compound **23**........ 35
Figure S62. ^{1}H NMR spectrum ($CDCl_3$, 400 MHz) of compound **24**........ 35
Figure S63. ^{1}H NMR spectrum ($CDCl_3$, 400 MHz) of compound **25**........ 36
Figure S64. ^{1}H NMR spectrum ($CDCl_3$, 400 MHz) of compound **26**........ 36

Figure S65. ^{1}H NMR spectrum ($CDCl_3$, 400 MHz) of compound **27**.. 37
Figure S66. ^{1}H NMR spectrum ($CDCl_3$, 400 MHz) of compound **28**.. 37
Figure S67. ^{1}H NMR spectrum ($CDCl_3$, 400 MHz) of compound **29**.. 38
Figure S68. ^{1}H NMR spectrum ($CDCl_3$, 400 MHz) of compound **30**.. 38
Figure S69. ^{1}H NMR spectrum ($CDCl_3$, 400 MHz) of compound **31**.. 39
Figure S70. ^{1}H NMR spectrum ($CDCl_3$, 400 MHz) of compound **32**.. 39

Table S1. In vitro antibacterial activity of compounds **1–3**, **5–11**, **13** and **15–32**.

Compound	MIC (μg/mL)	
	S. aureus (ATCC-25923)	*E. coli* (NCTC-10418)
sagonenyne (**1**)	not active[1]	not active[1]
10-acetyl-sagonenyne (**2**)	not active[1]	not active[1]
cis-sagonenyne (**3**)	not active[1]	not active[1]
compound **5**	not active[1]	not active[1]
rogioloxepane C (**6**)	not active[1]	not active[1]
rogiolenyne B (**7**)	128	not active[1]
trans-pinnatifidenyne (**8**)	not active[1]	not active[1]
(3*E*)-laurenyne (**9**)	not active[1]	not active[1]
marilzallene B (**10**)	not active[1]	not active[1]
tinosallene A (**11**)	not active[1]	not active[1]
(3*E*,6*R*,7*R*)-obtusenyne (**13**)	not active[1]	not active[1]
10-bromo-obtusallene I (**15**)	not active[1]	not active[1]
obtusallene VII (**16**)	not active[1]	not active[1]
obtusallene XI (**17**)	not active[1]	not active[1]
obtusallene IV (**18**)	not active[1]	not active[1]
obtusallene VI (**19**)	not active[1]	not active[1]
obtusallene V (**20**)	not active[1]	not active[1]
chondrioallene (**21**)	not active[1]	not active[1]
compound **22**	not active[1]	not active[1]
compound **23**	not active[1]	not active[1]
iso-laurenisol (**24**)	16	not active[1]
bromolaurenisol (**25**)	4	not active[1]
α-snyderol (**26**)	not active[1]	not active[1]
obtusenol (**27**)	not active[1]	not active[1]
perforenol (**28**)	not active[1]	not active[1]
compound **29**	not active[1]	not active[1]
trans-nerolidol (**30**)	not active[1]	not active[1]
isopinnatol B (**31**)	not active[1]	not active[1]
deoxyparguerol (**32**)	not active[1]	not active[1]
vancomycin	1	not tested
chloramphenicol	not tested	4

[1] At a concentration of 128 μg/mL.

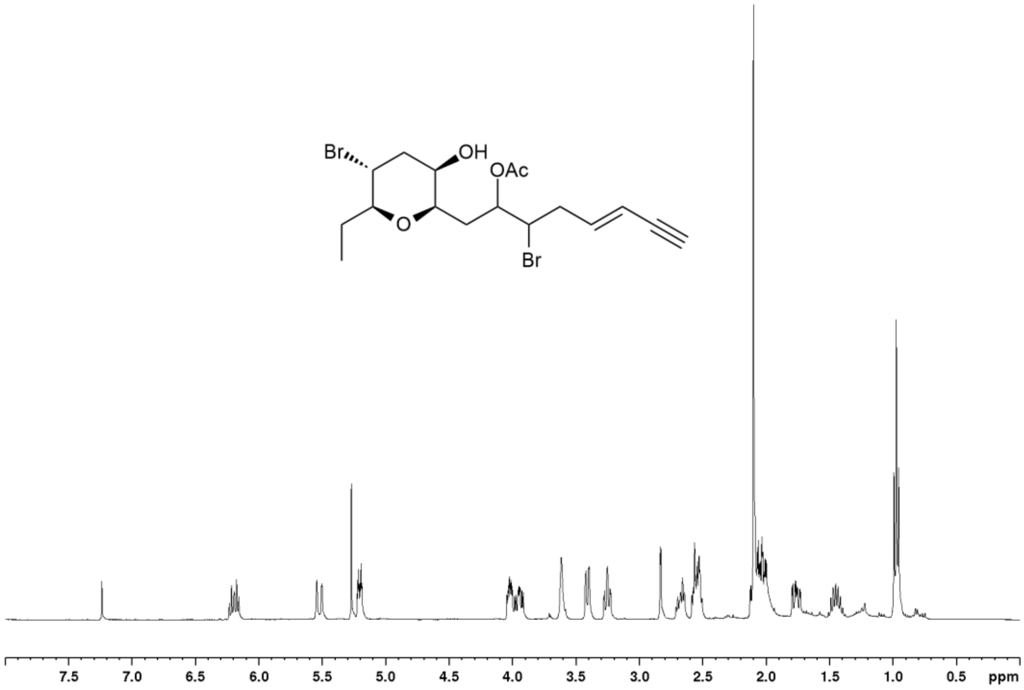

Figure S1. ^{1}H NMR spectrum ($CDCl_3$, 400 MHz) of compound **1**.

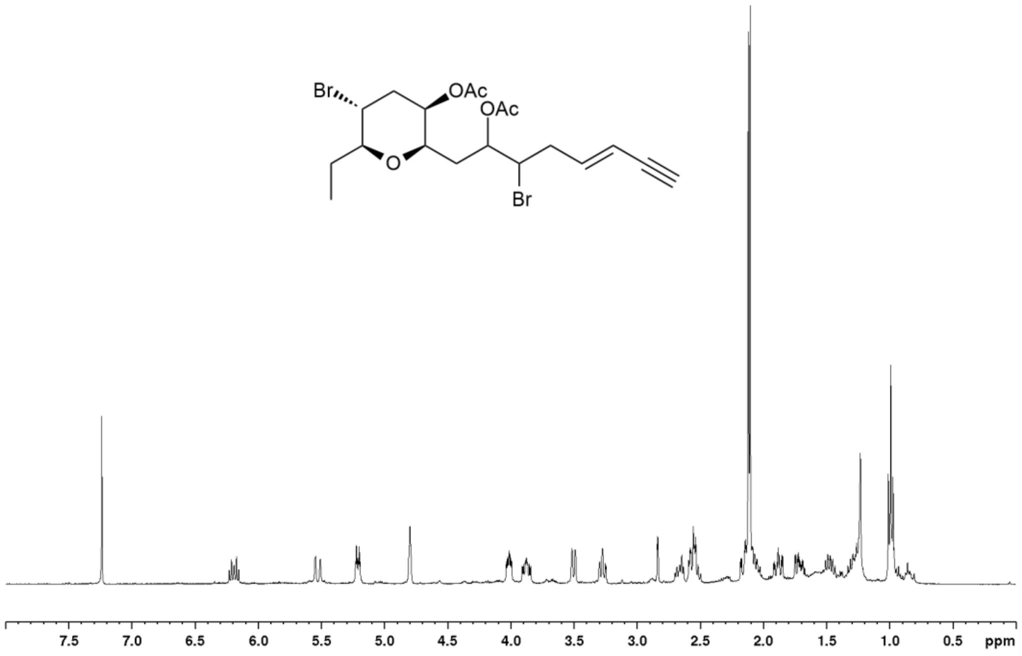

Figure S2. ^{1}H NMR spectrum ($CDCl_3$, 400 MHz) of compound **2**.

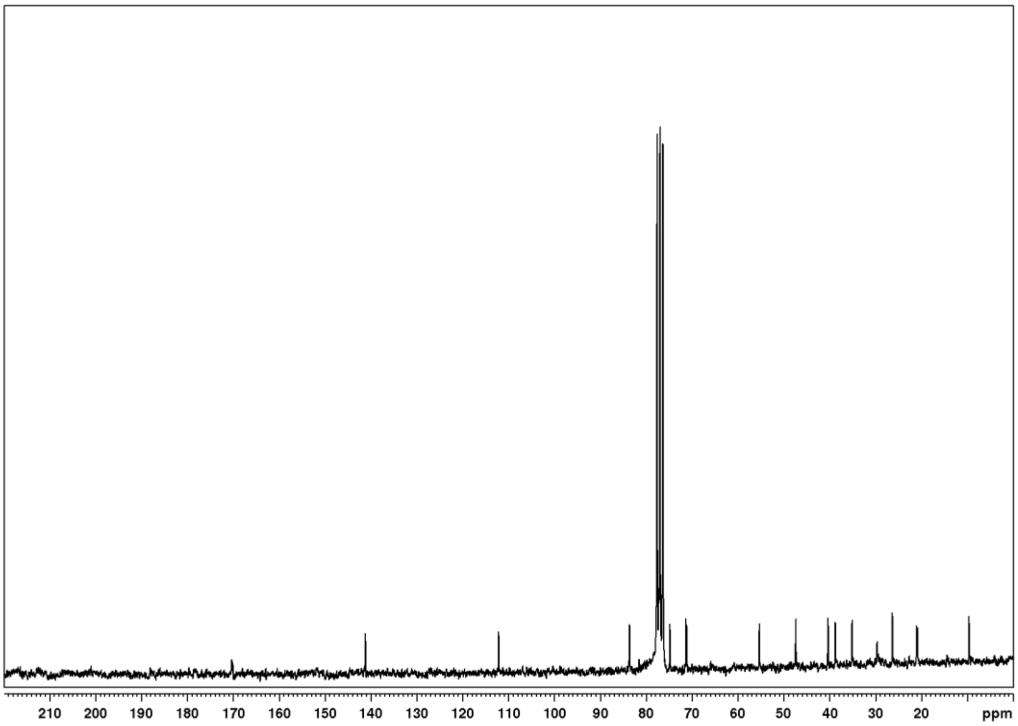

Figure S3. ^{13}C NMR spectrum ($CDCl_3$, 50.3 MHz) of compound **2**.

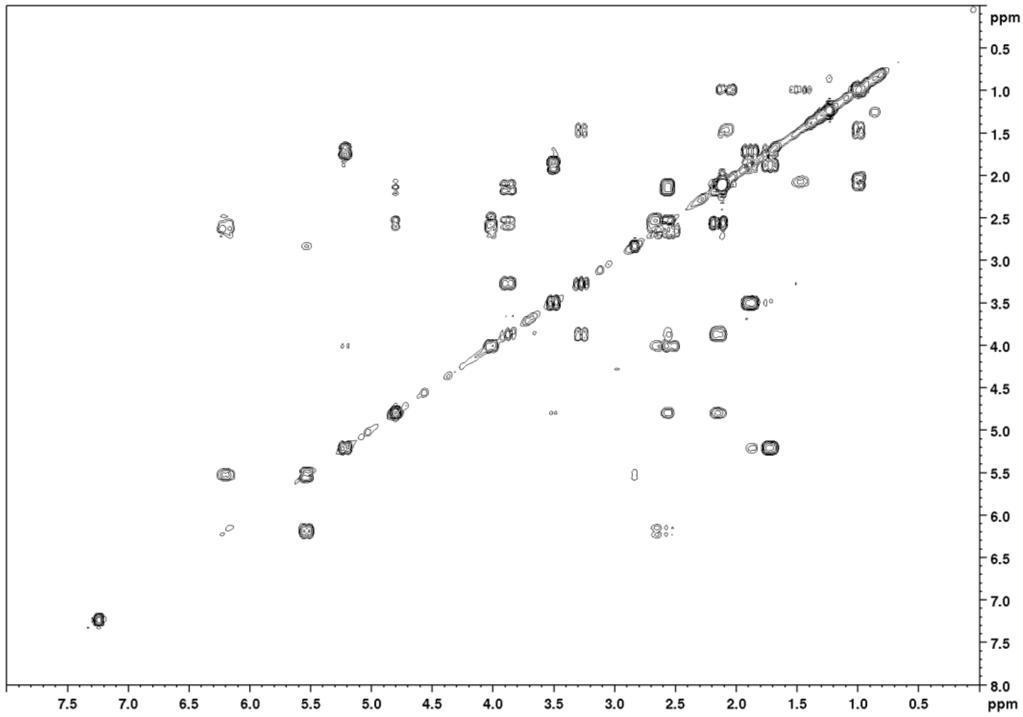

Figure S4. COSY spectrum ($CDCl_3$, 400 MHz) of compound **2**.

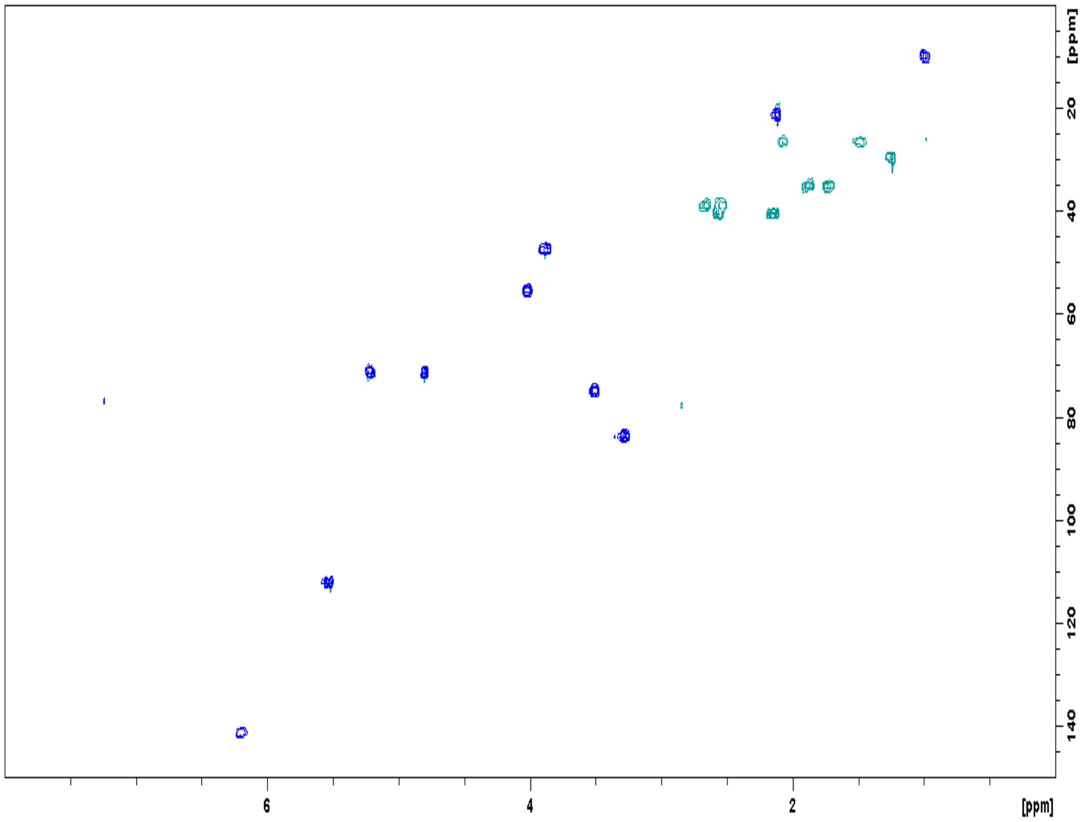

Figure S5. HSQC spectrum ($CDCl_3$, 400 MHz) of compound **2**.

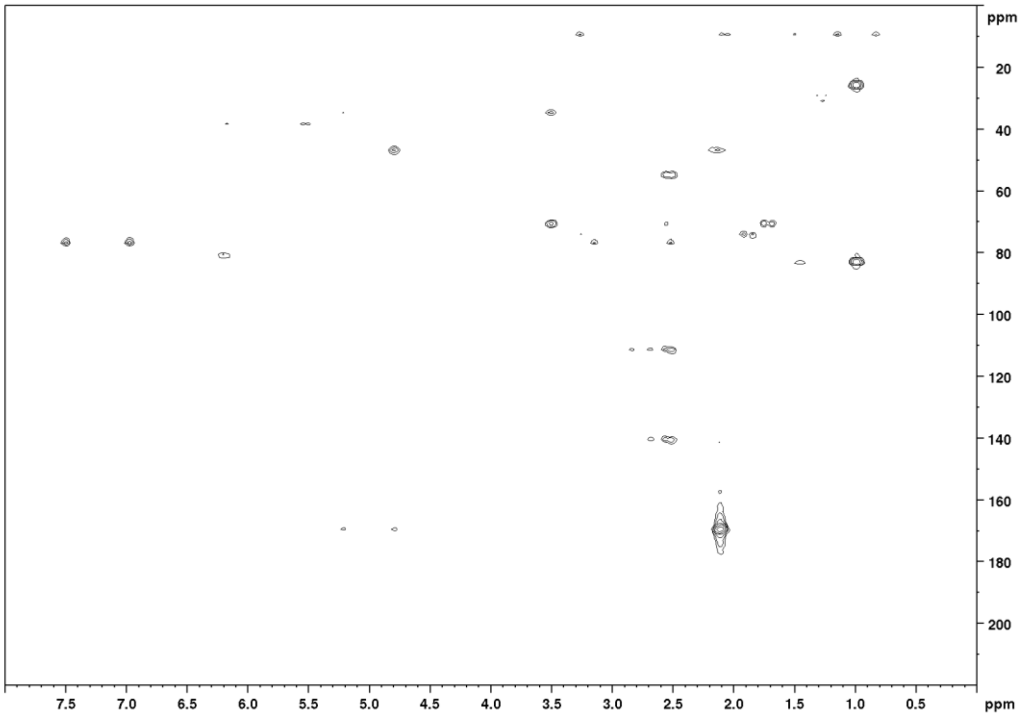

Figure S6. HMBC spectrum ($CDCl_3$, 400 MHz) of compound **2**.

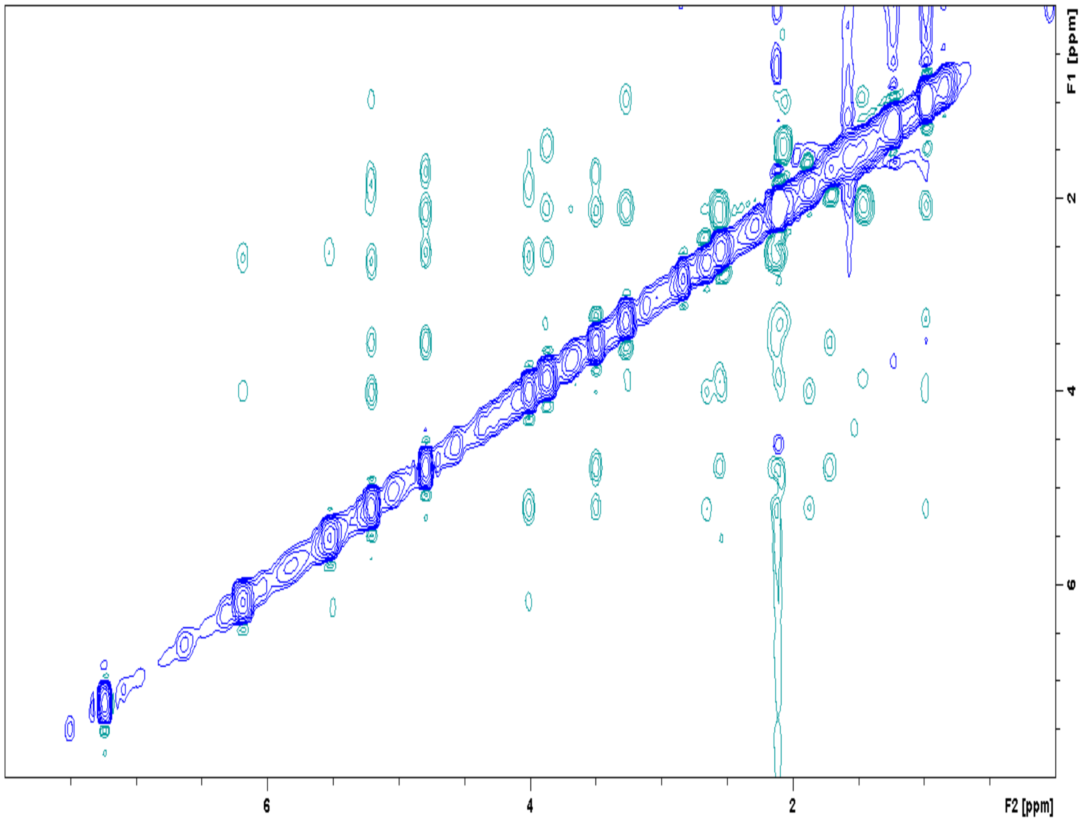

Figure S7. NOESY spectrum ($CDCl_3$, 400 MHz) of compound **2**.

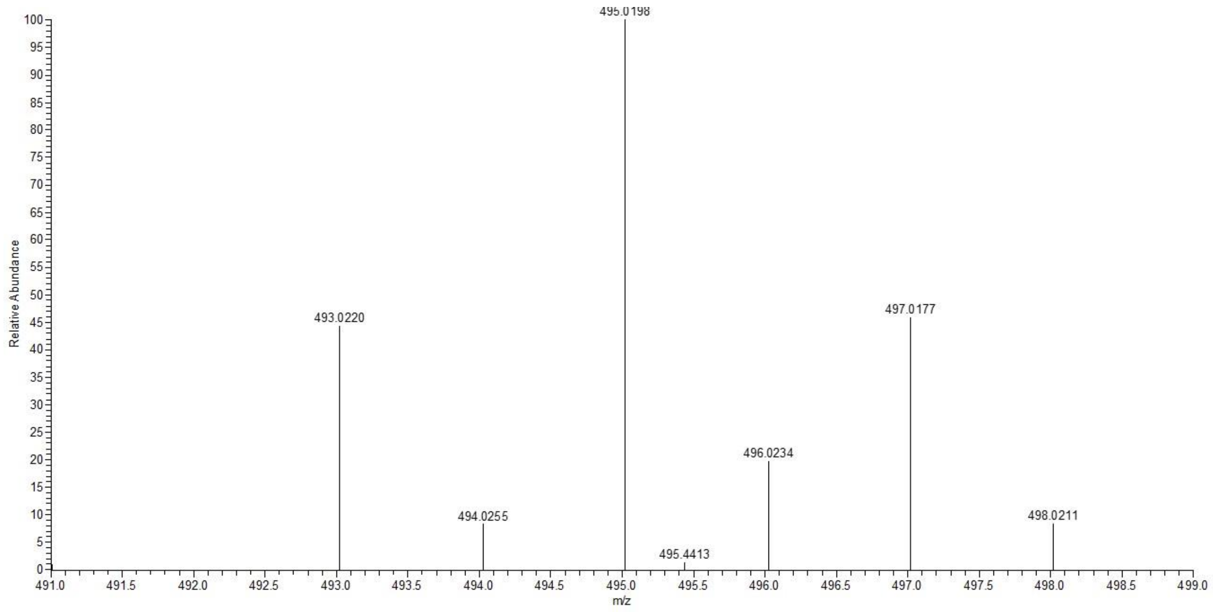

Figure S8. HR-APCIMS of compound **2**.

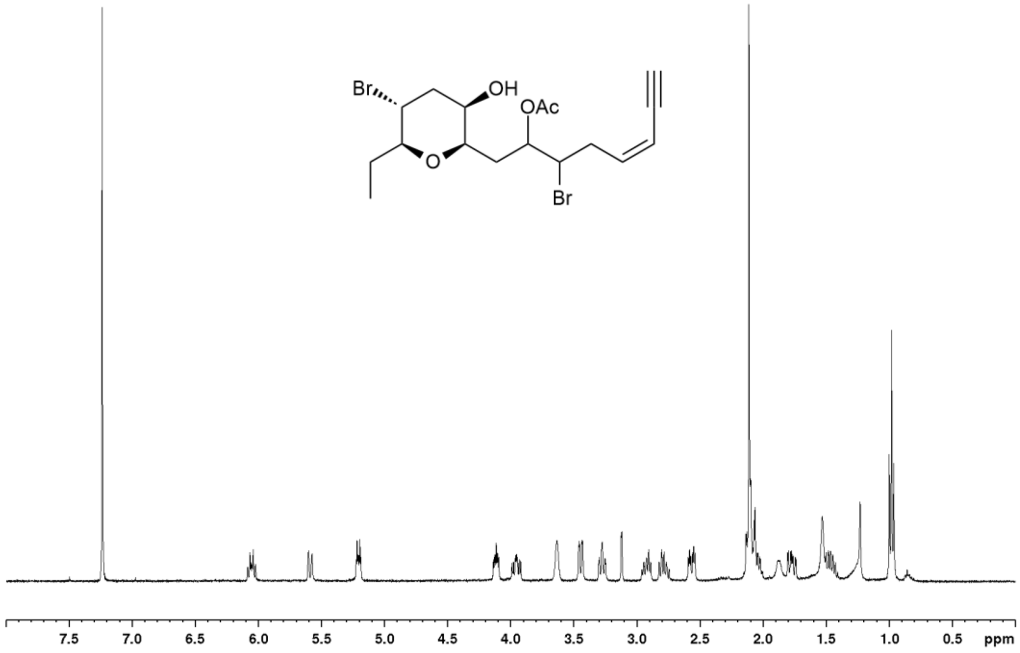

Figure S9. ^{1}H NMR spectrum ($CDCl_3$, 400 MHz) of compound **3**.

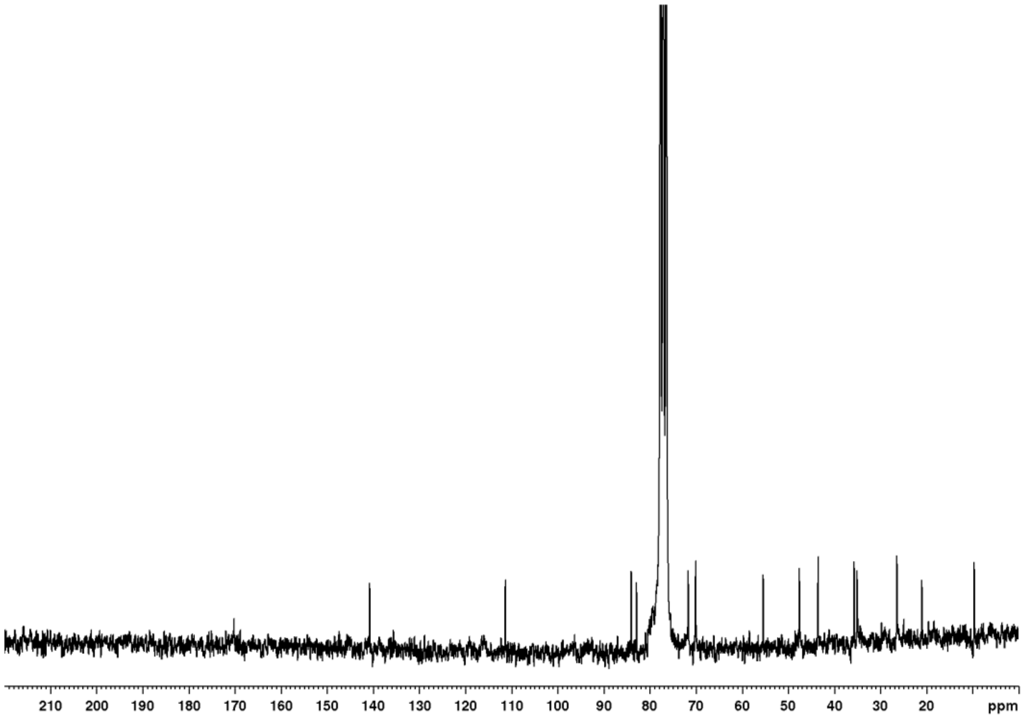

Figure S10. ^{13}C NMR spectrum ($CDCl_3$, 50.3 MHz) of compound **3**.

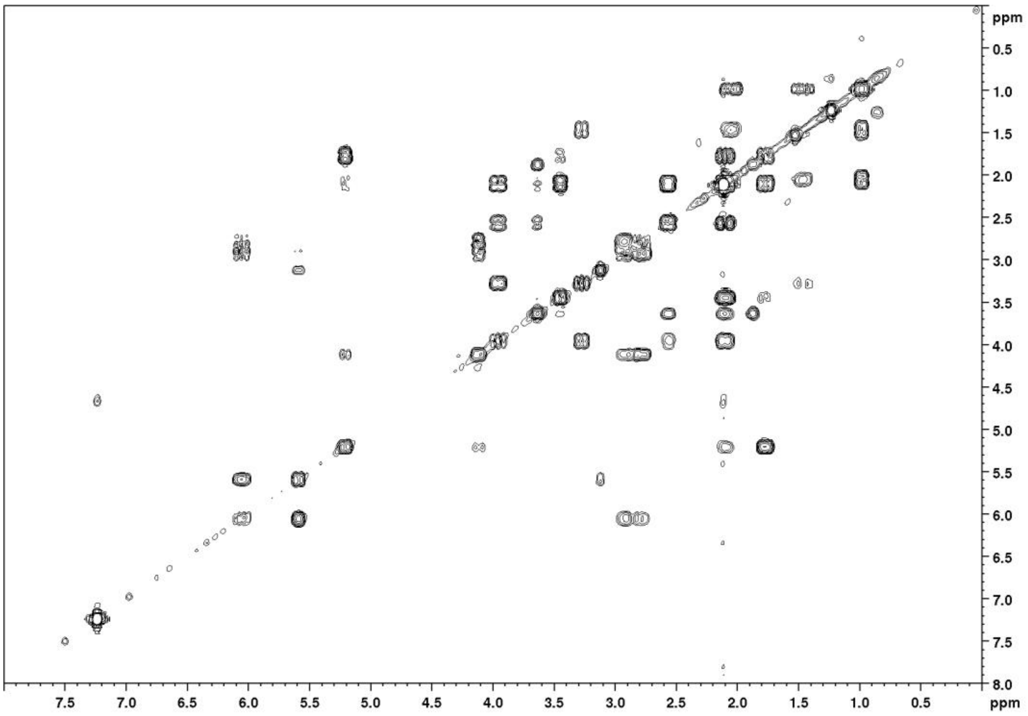

Figure S11. COSY spectrum ($CDCl_3$, 400 MHz) of compound **3**.

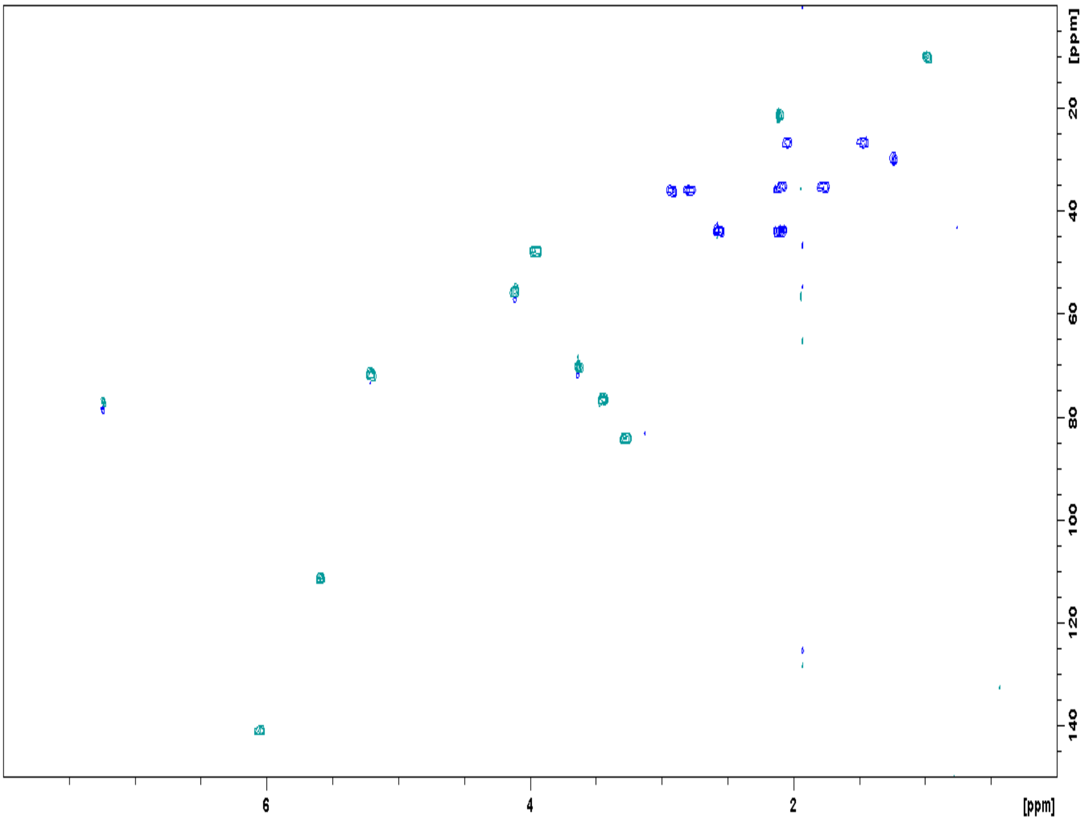

Figure S12. HSQC spectrum ($CDCl_3$, 400 MHz) of compound **3**.

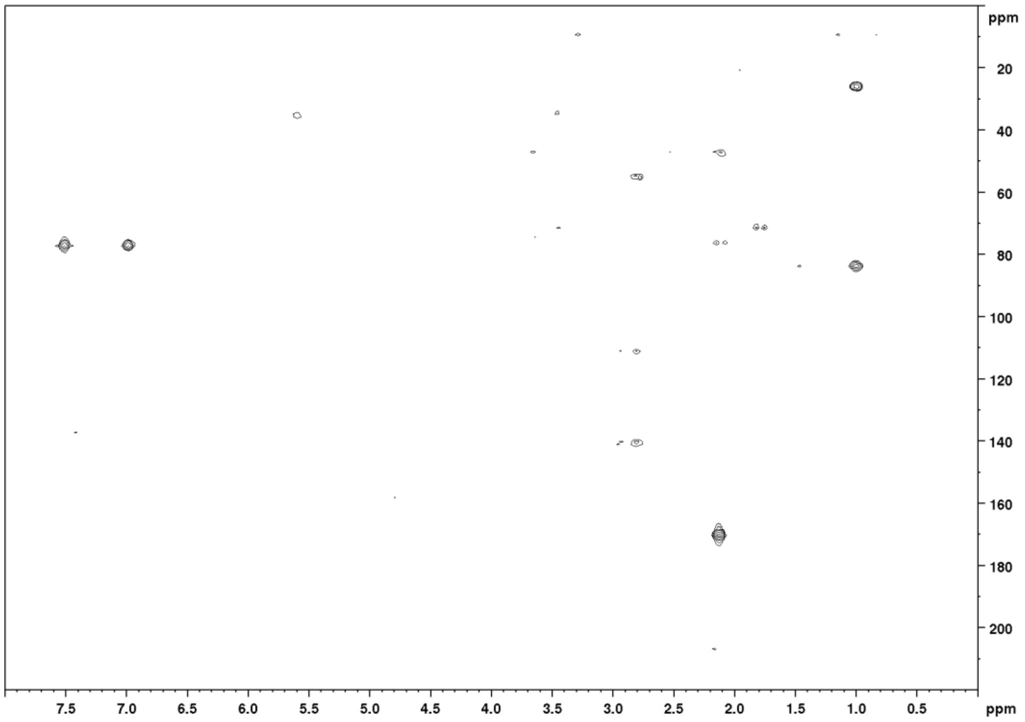

Figure S13. HMBC spectrum ($CDCl_3$, 400 MHz) of compound **3**.

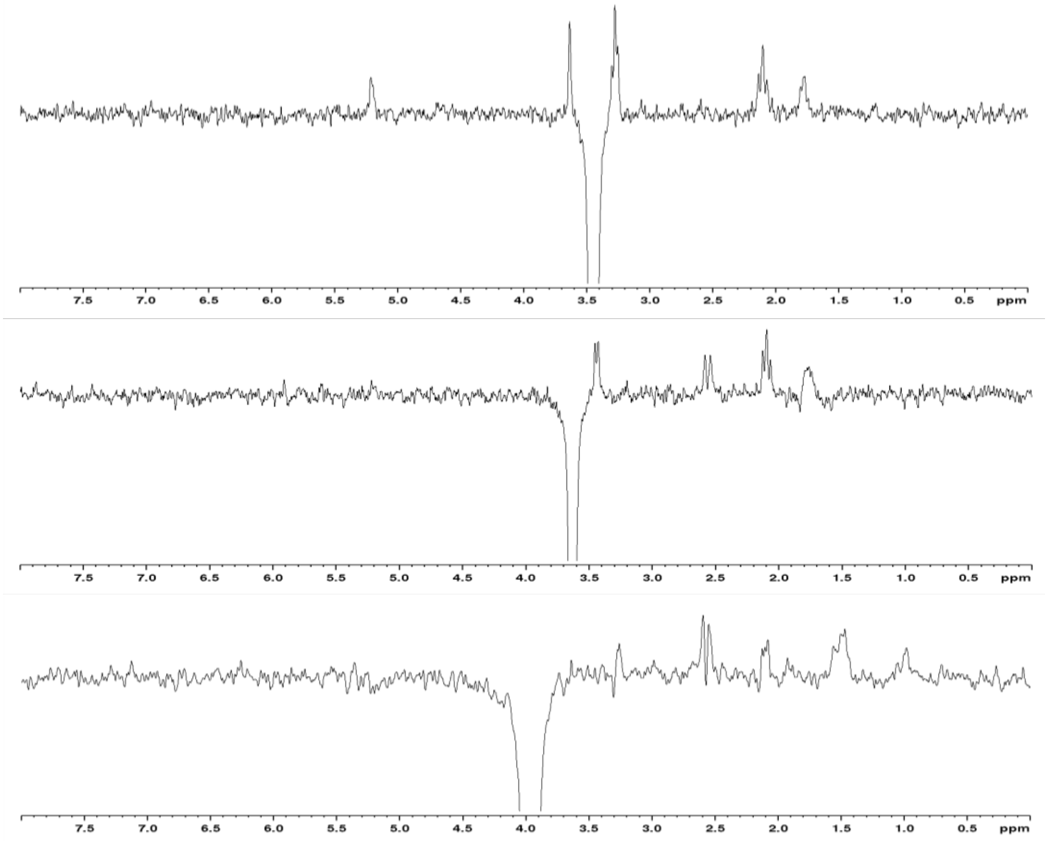

Figure S14. 1D-NOE spectra ($CDCl_3$, 400 MHz) of compound **3**.

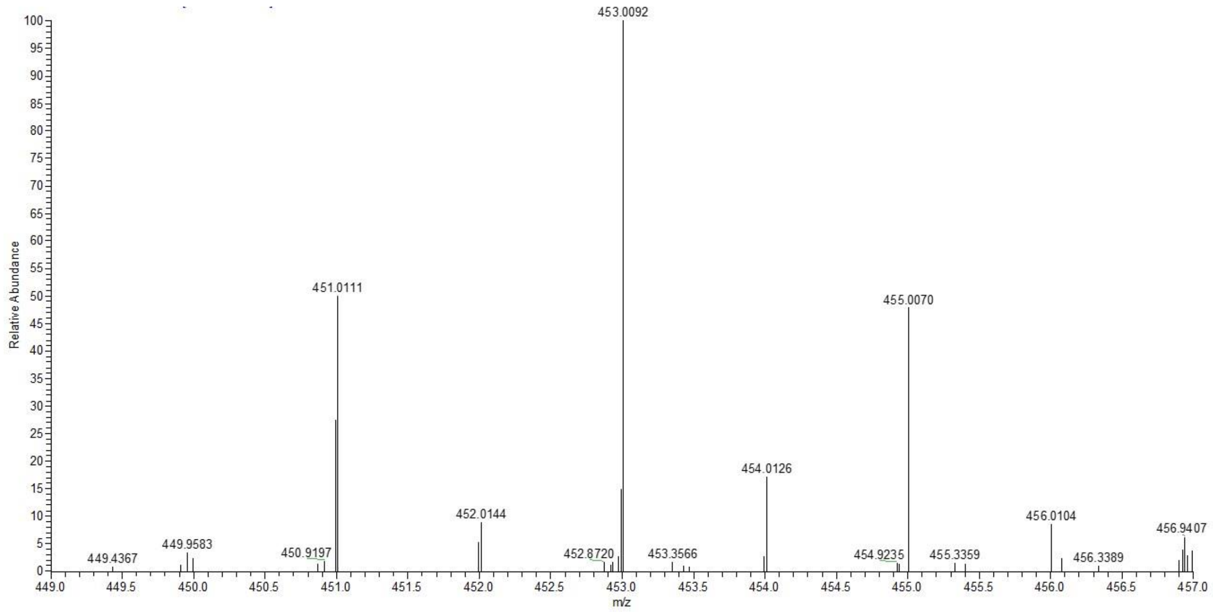

Figure S15. HR-APCIMS of compound **3**.

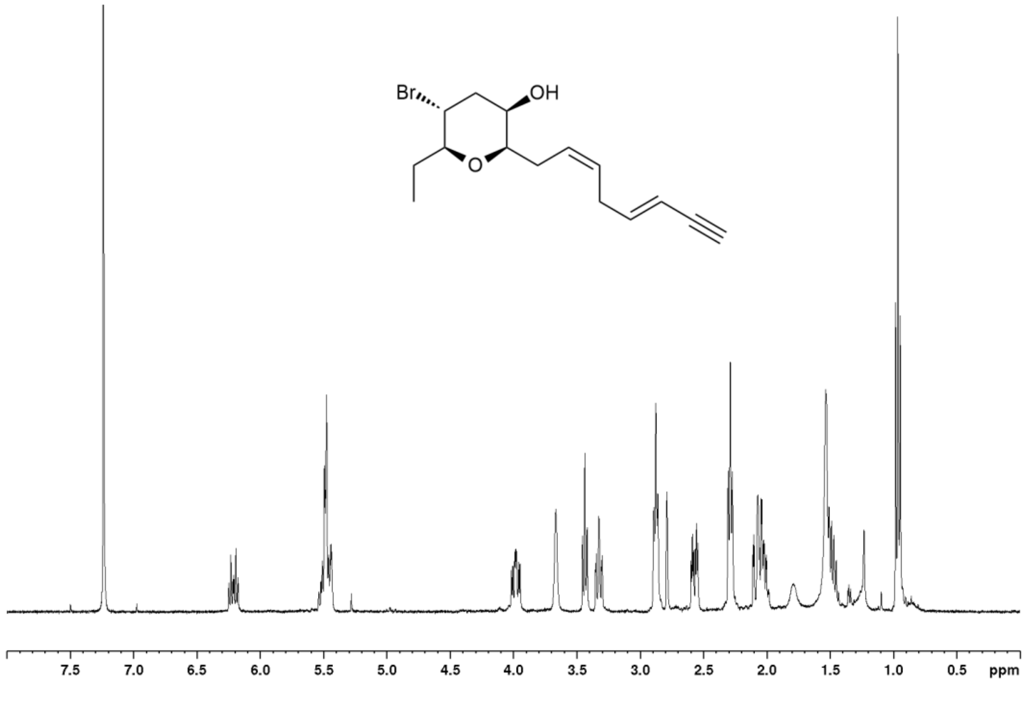

Figure S16. ^{1}H NMR spectrum ($CDCl_3$, 400 MHz) of compound **4**.

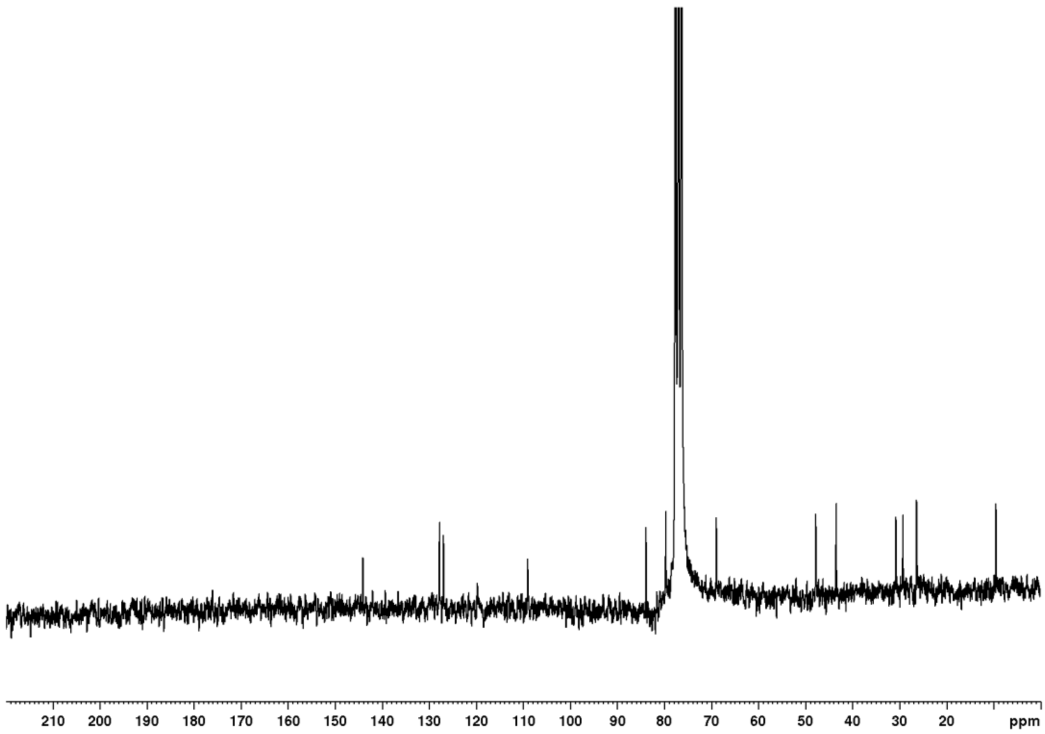

Figure S17. ^{13}C NMR spectrum ($CDCl_3$, 50.3 MHz) of compound **4**.

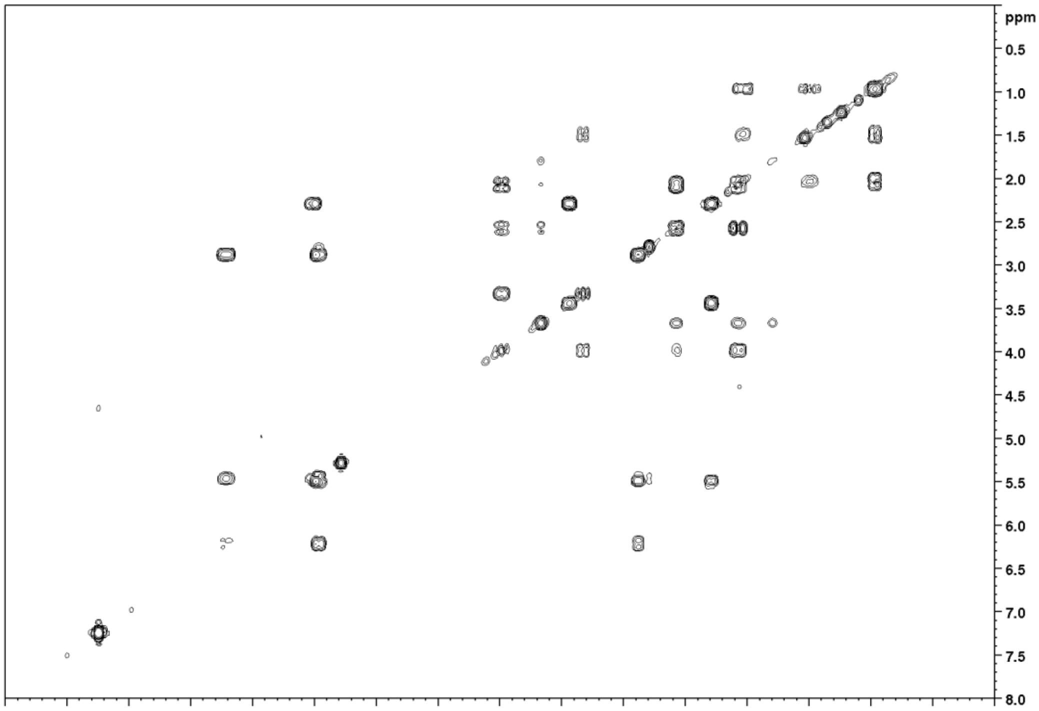

Figure S18. COSY spectrum ($CDCl_3$, 400 MHz) of compound **4**.

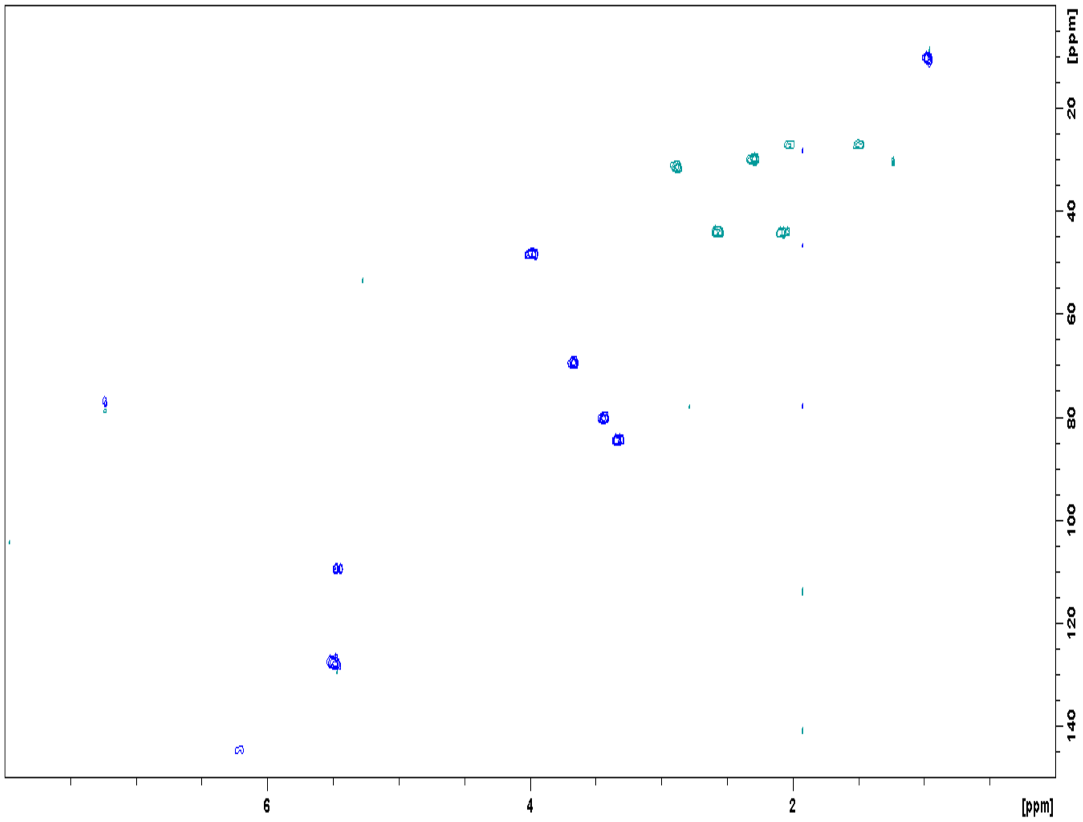

Figure S19. HSQC spectrum ($CDCl_3$, 400 MHz) of compound **4**.

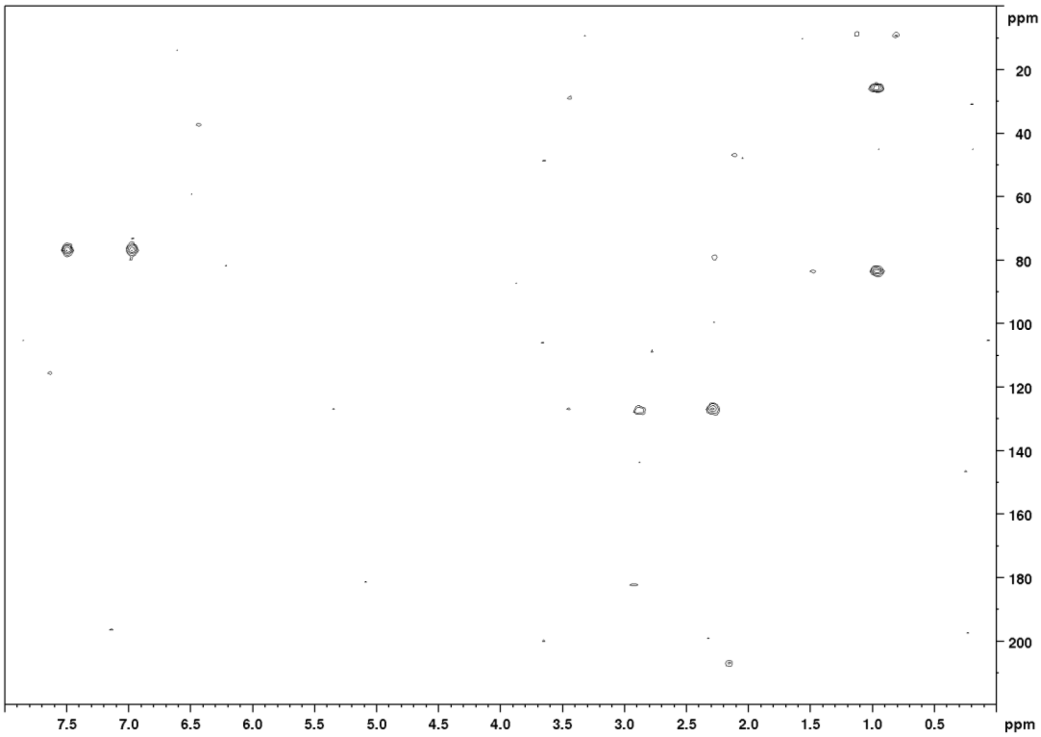

Figure S20. HMBC spectrum ($CDCl_3$, 400 MHz) of compound **4**.

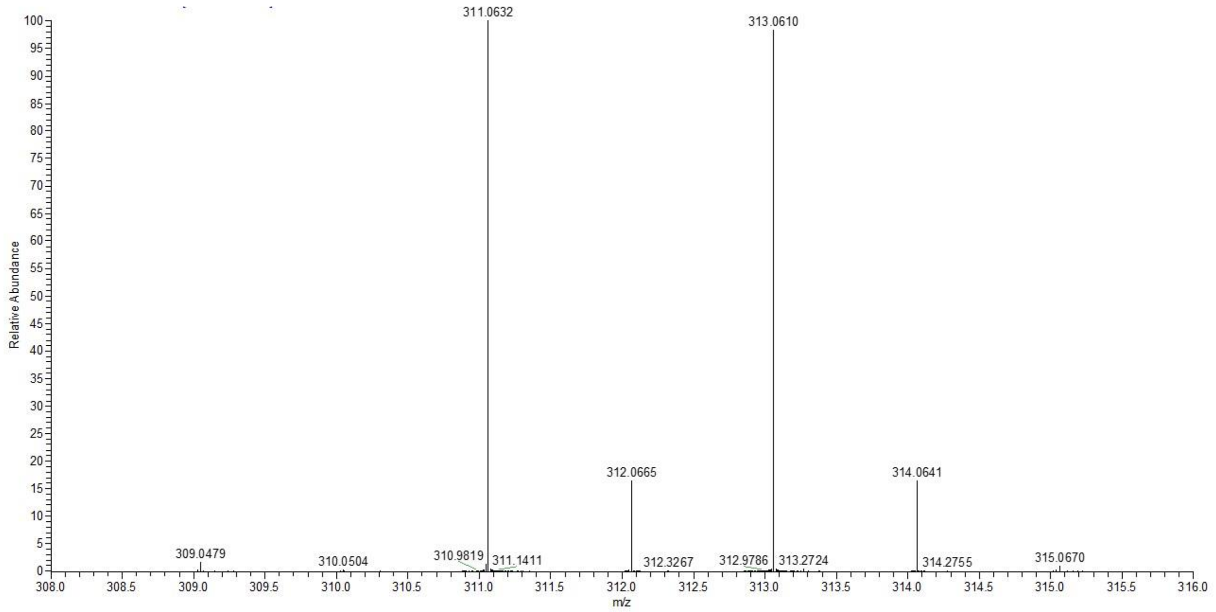

Figure S21. HR-APCIMS of compound **4**.

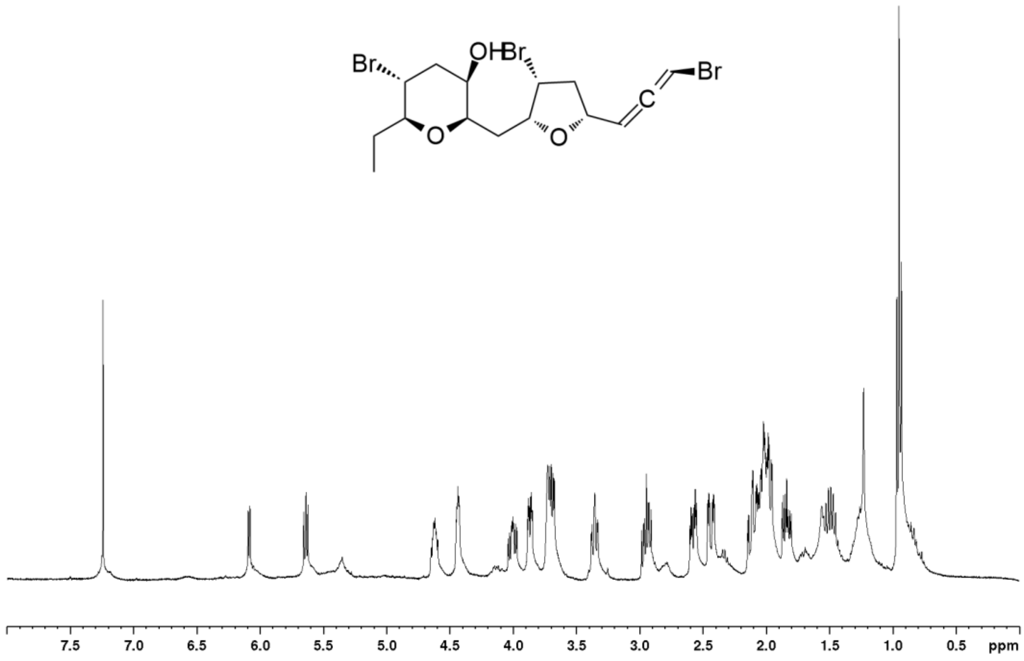

Figure S22. ^{1}H NMR spectrum ($CDCl_3$, 400 MHz) of compound **5**.

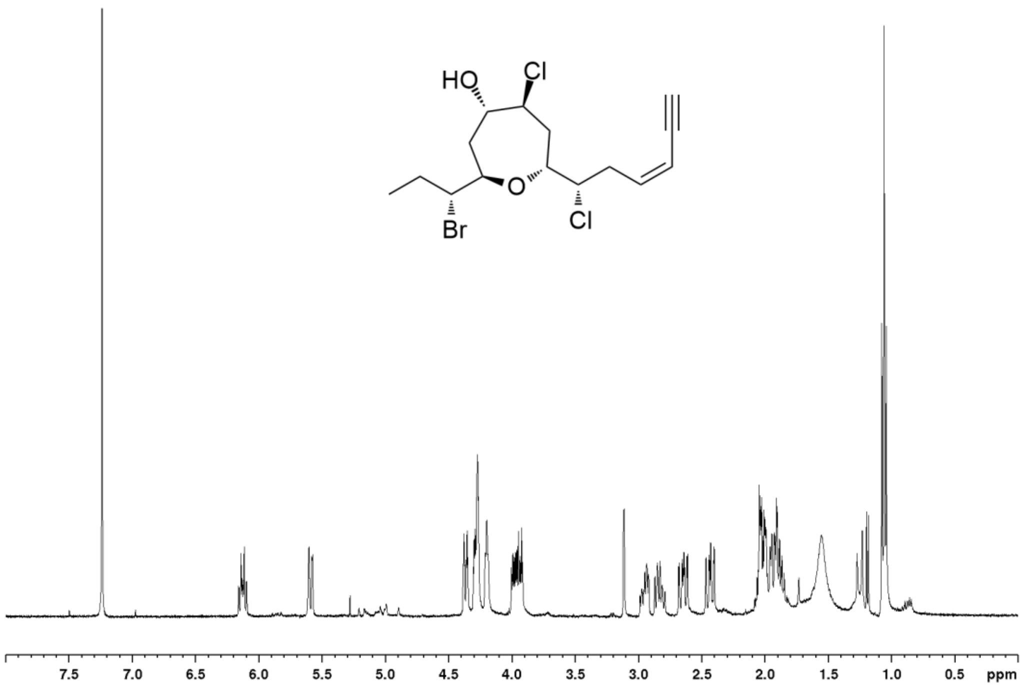

Figure S23. ^{1}H NMR spectrum ($CDCl_3$, 400 MHz) of compound **6**.

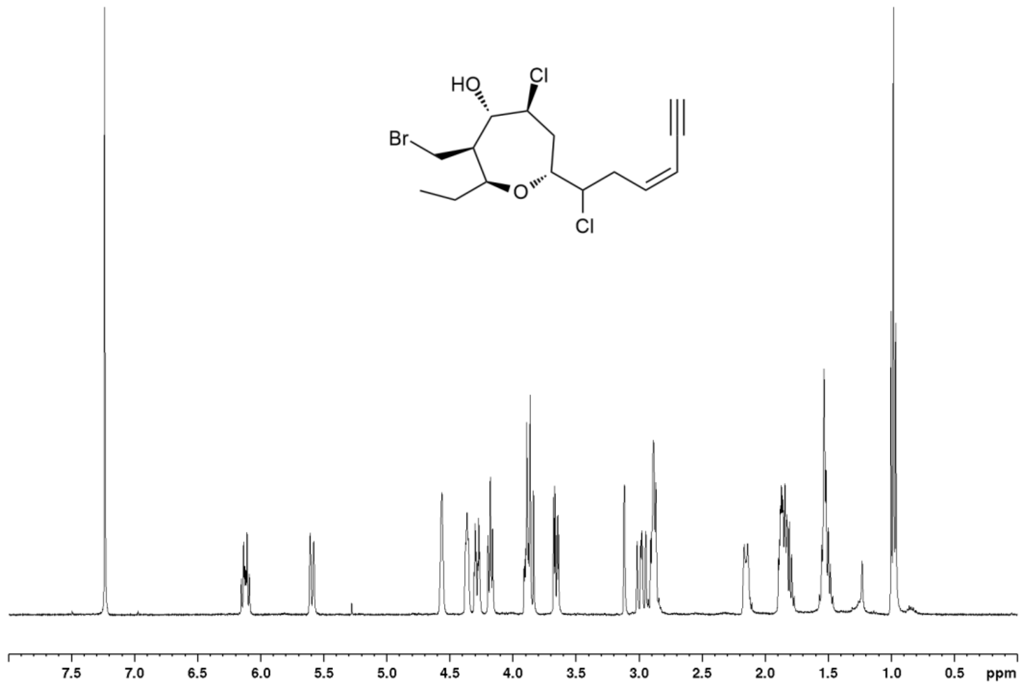

Figure S24. ^{1}H NMR spectrum ($CDCl_3$, 400 MHz) of compound **7**.

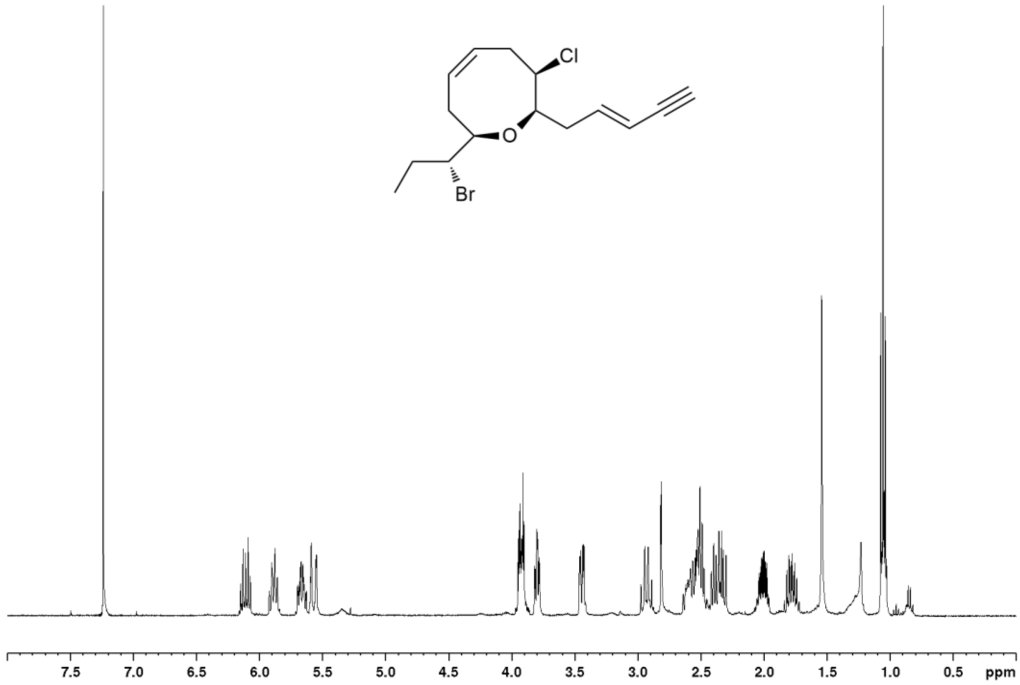

Figure S25. ^{1}H NMR spectrum ($CDCl_3$, 400 MHz) of compound **8**.

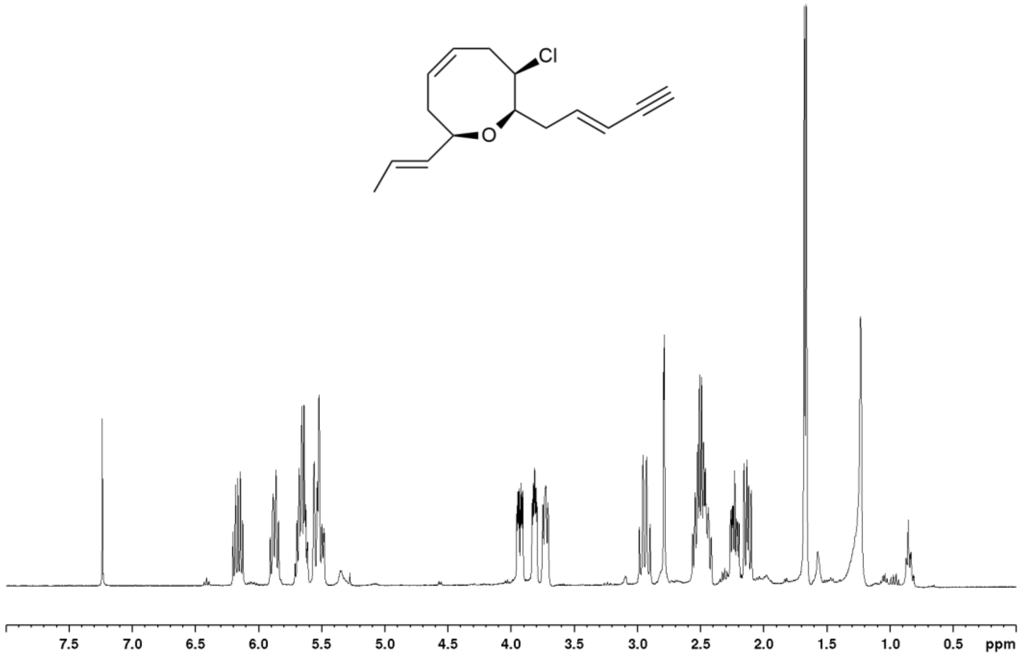

Figure S26. ^{1}H NMR spectrum ($CDCl_3$, 400 MHz) of compound **9**.

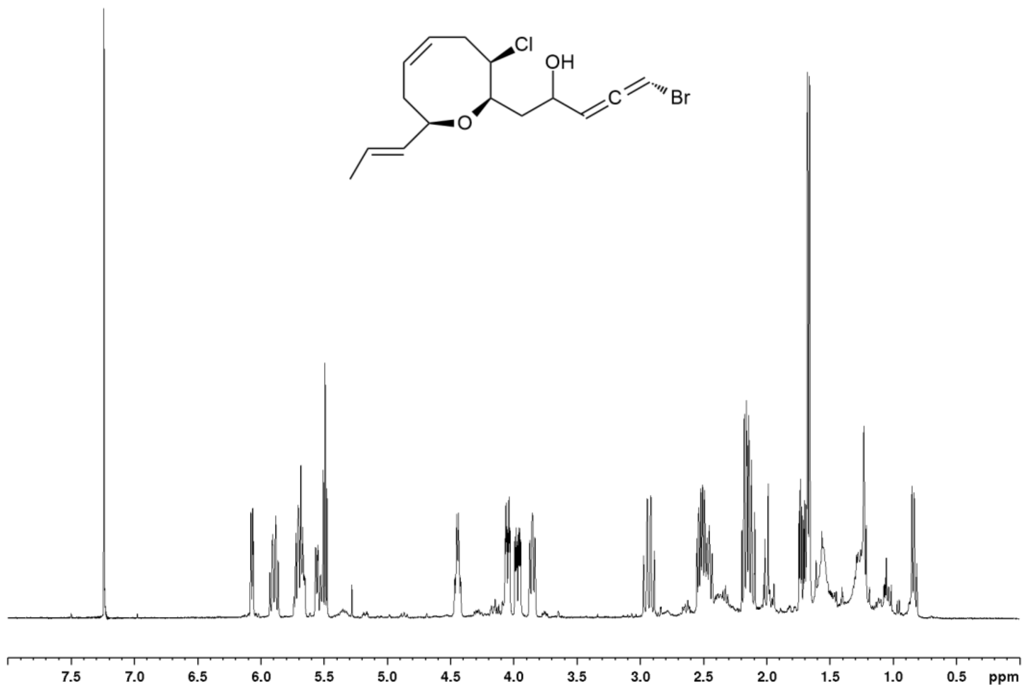

Figure S27. ¹H NMR spectrum ($CDCl_3$, 400 MHz) of compound **10**.

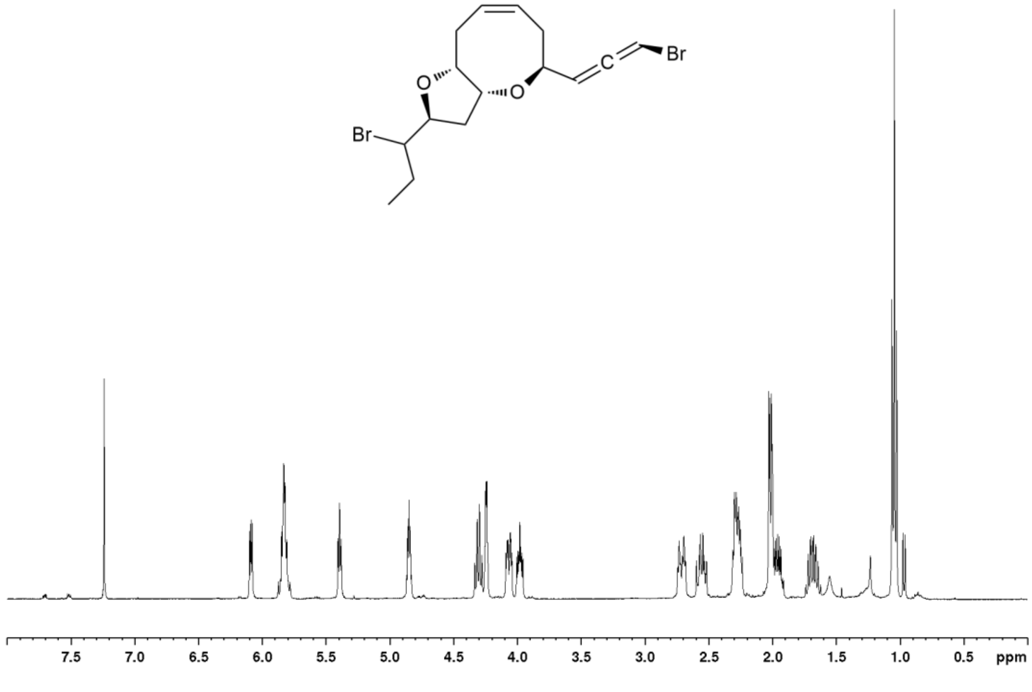

Figure S28. ¹H NMR spectrum ($CDCl_3$, 400 MHz) of compound **11**.

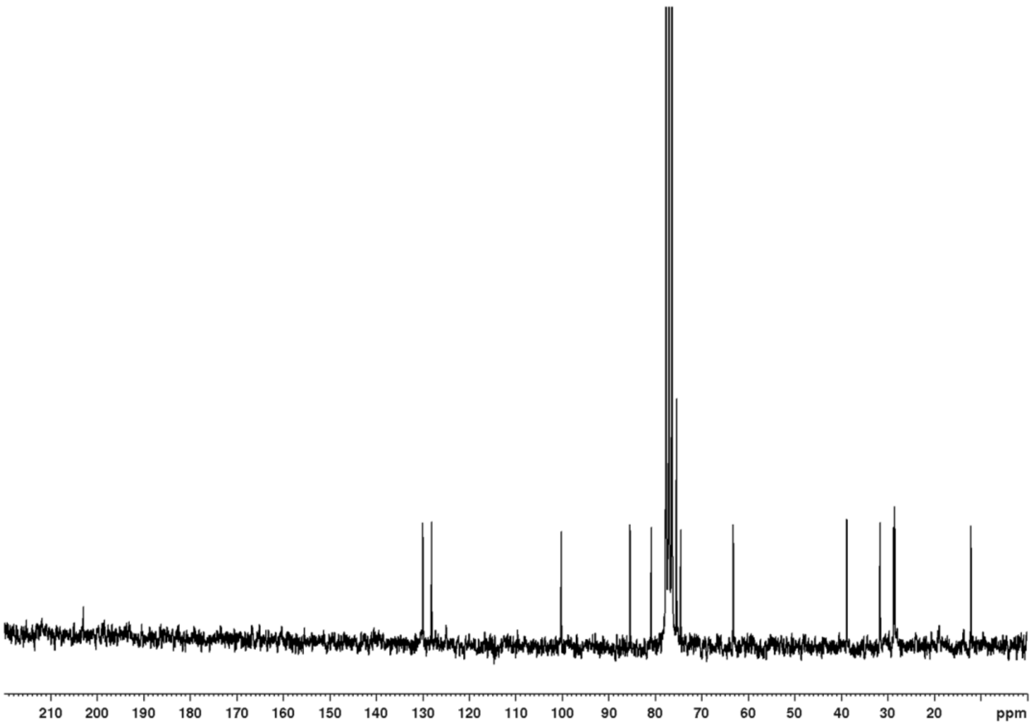

Figure S29. ^{13}C NMR spectrum ($CDCl_3$, 50.3 MHz) of compound **11**.

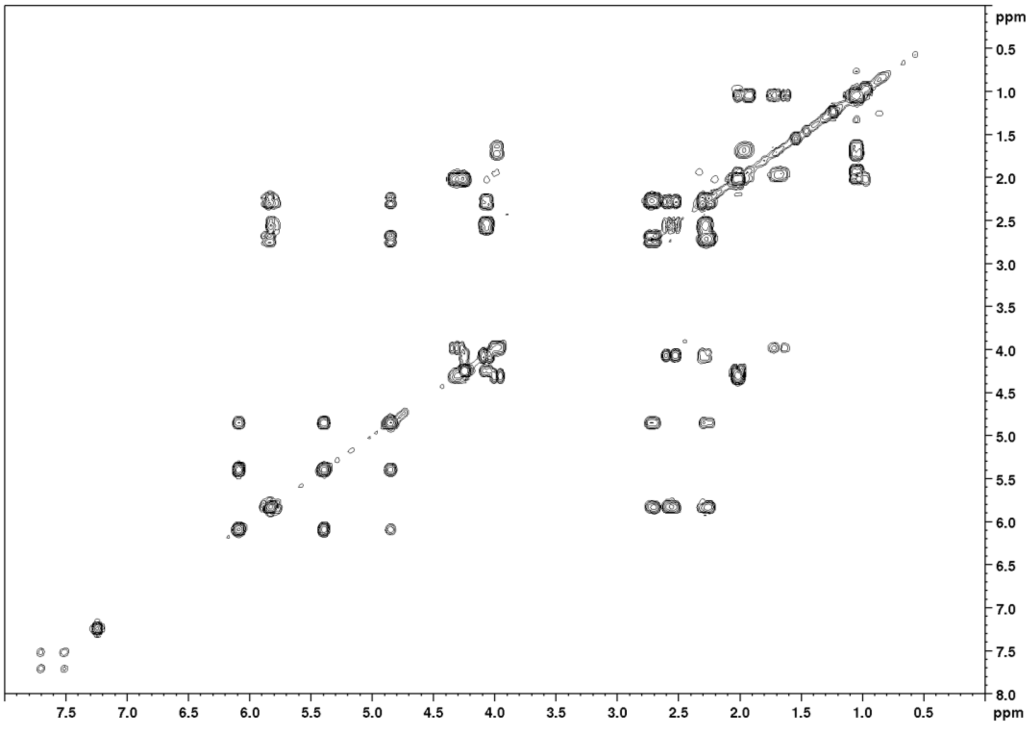

Figure S30. COSY spectrum ($CDCl_3$,400 MHz) of compound **11**.

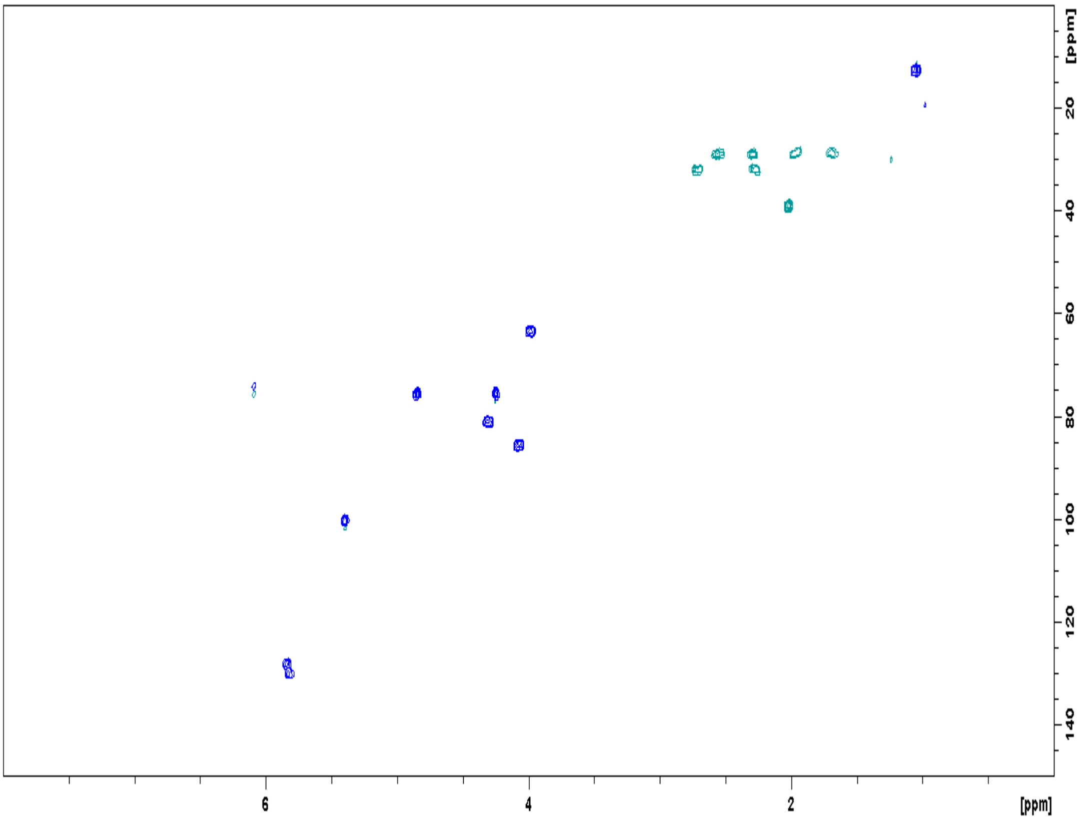

Figure S31. HSQC spectrum ($CDCl_3$, 400 MHz) of compound **11**.

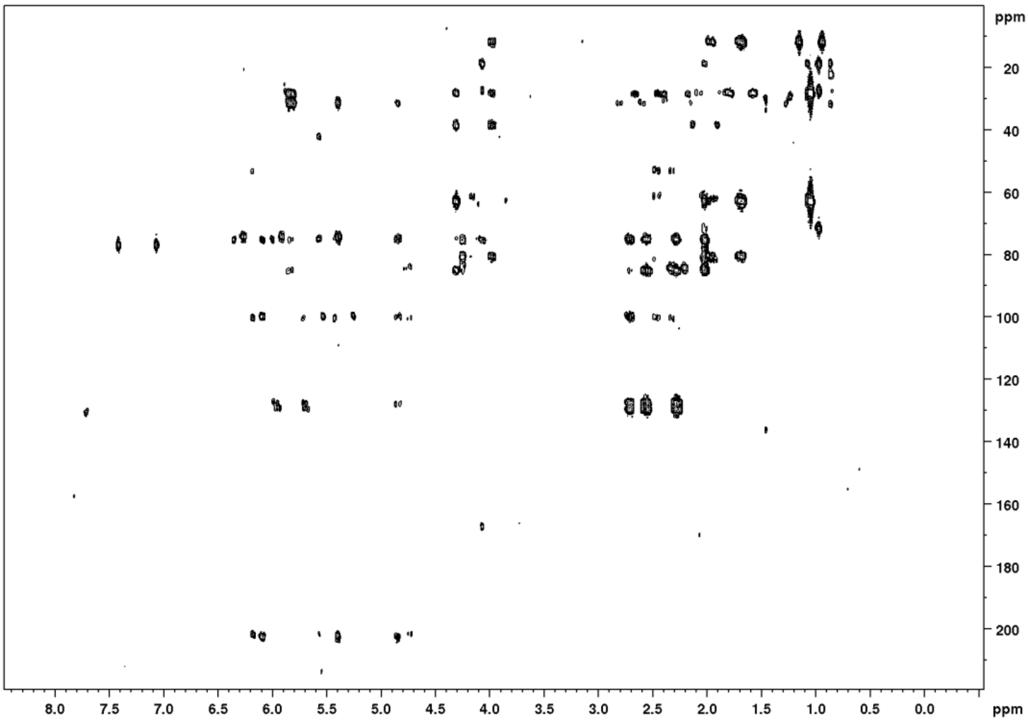

Figure S32. HMBC spectrum ($CDCl_3$, 600 MHz) of compound **11**.

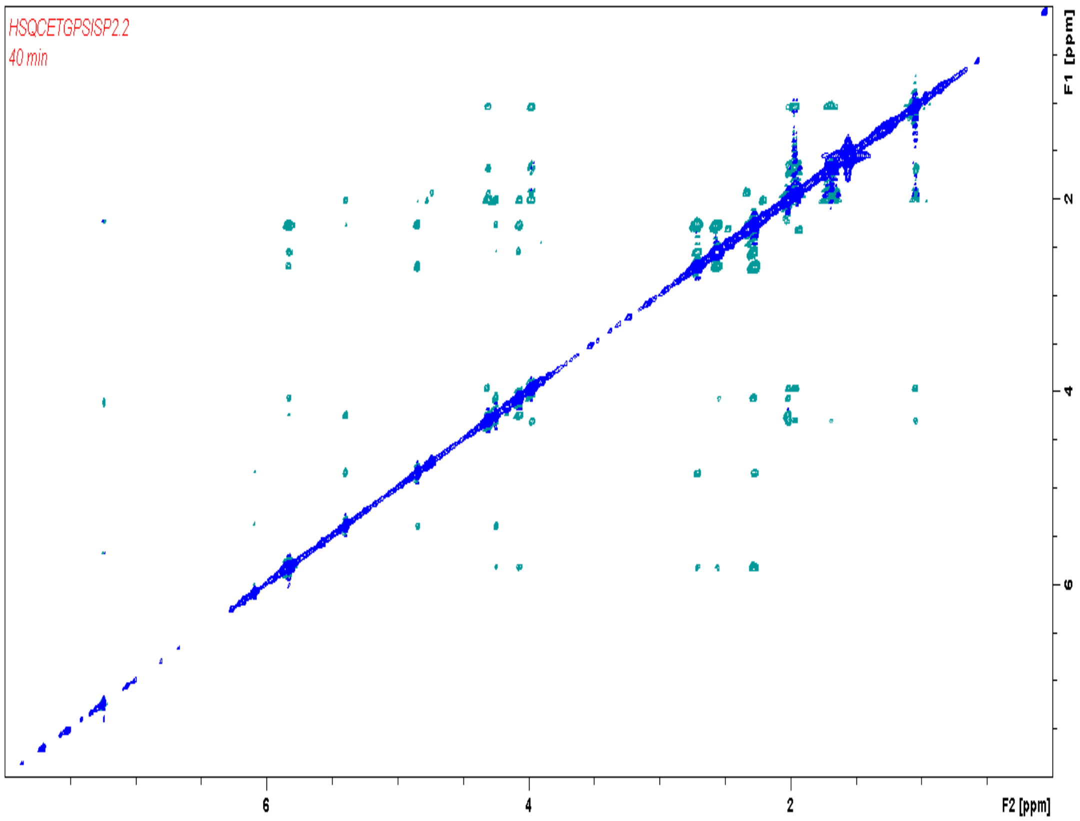

Figure S33. NOESY spectrum ($CDCl_3$, 600 MHz) of compound **11**.

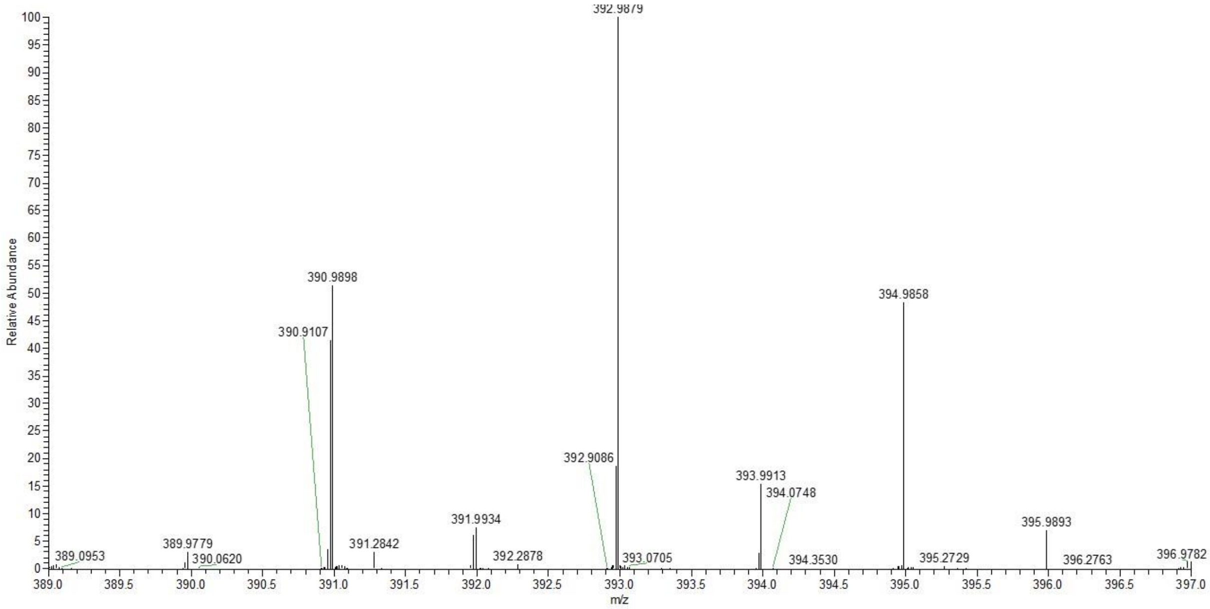

Figure S34. HR-APCIMS of compound **11**.

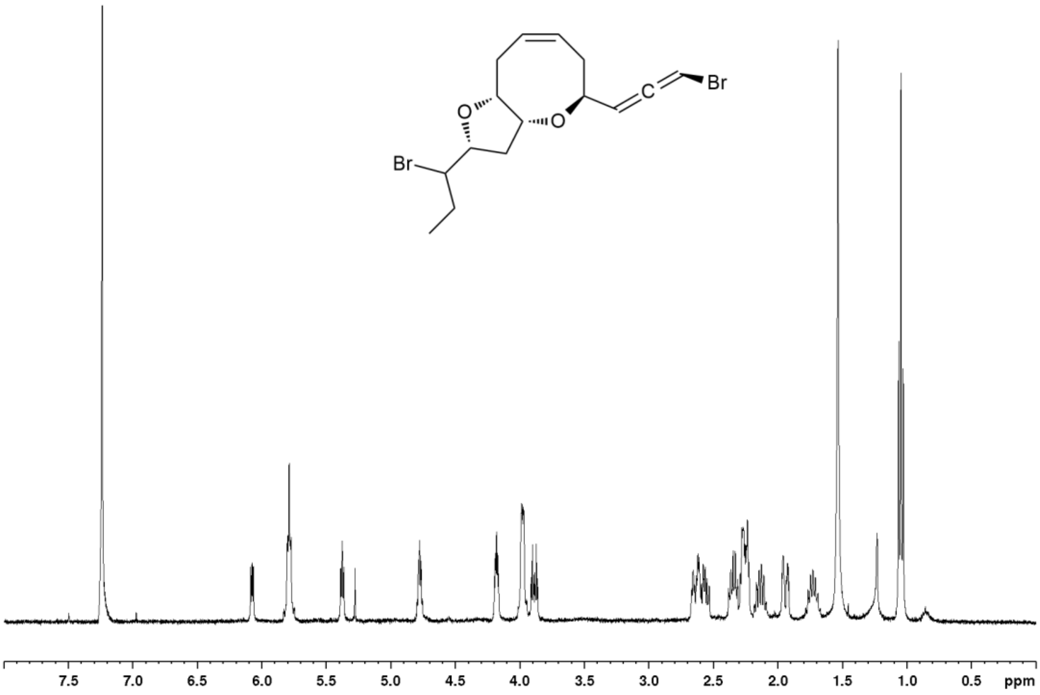

Figure S35. ^{1}H NMR spectrum ($CDCl_3$, 400 MHz) of compound **12**.

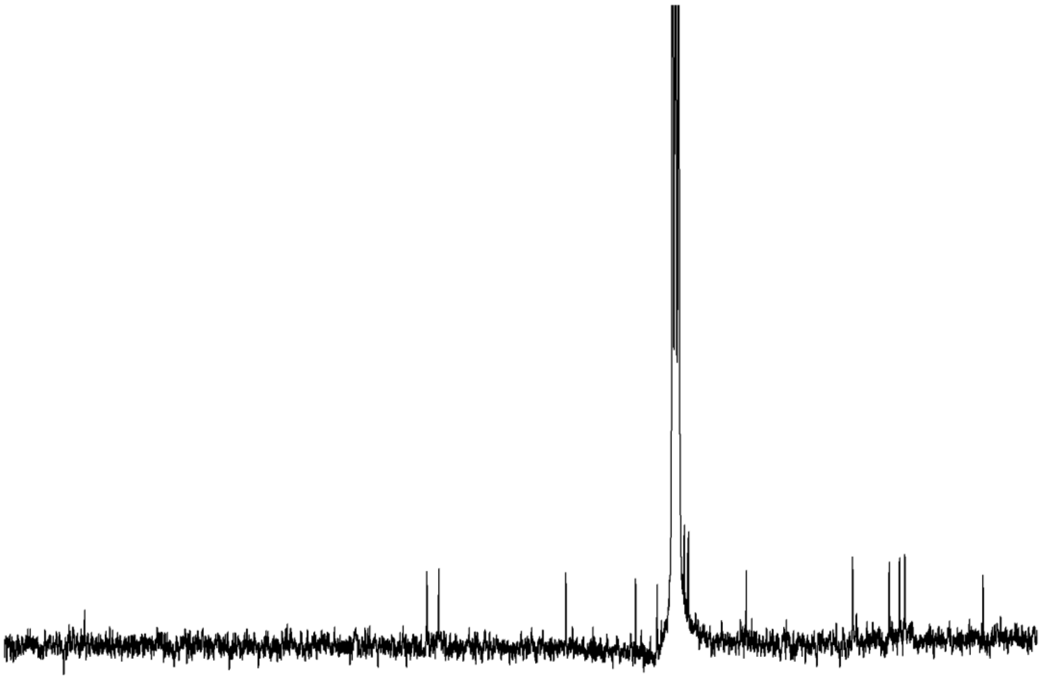

Figure S36. ^{13}C NMR spectrum ($CDCl_3$, 50.3 MHz) of compound **12**.

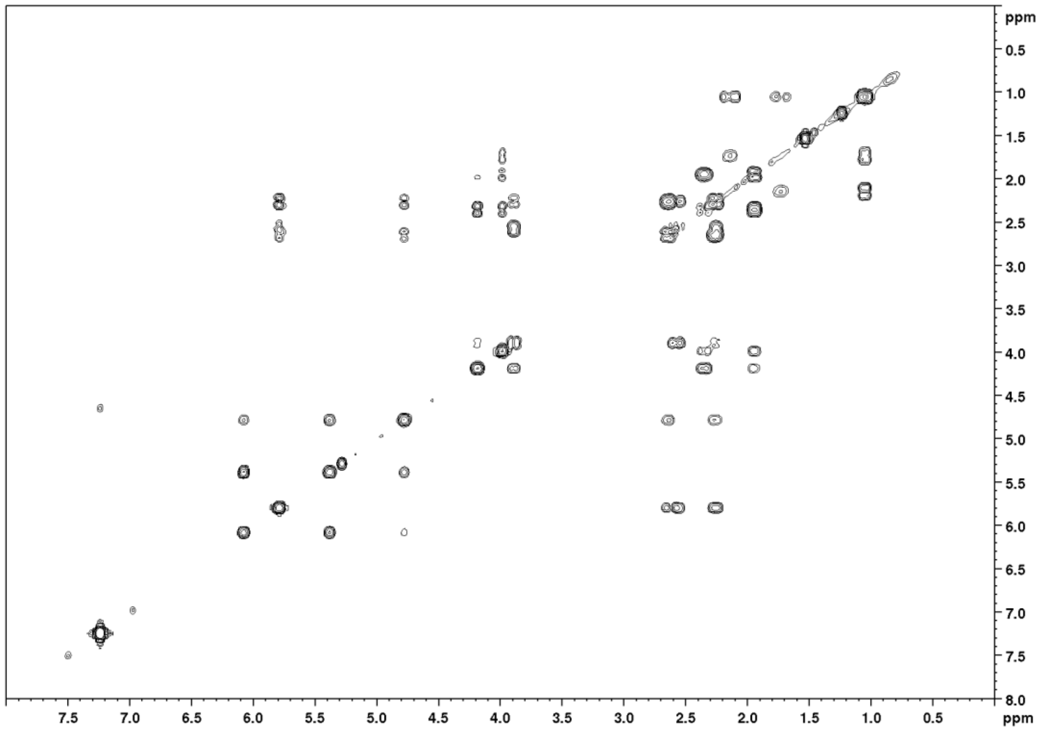

Figure S37. COSY spectrum ($CDCl_3$, 400 MHz) of compound **12**.

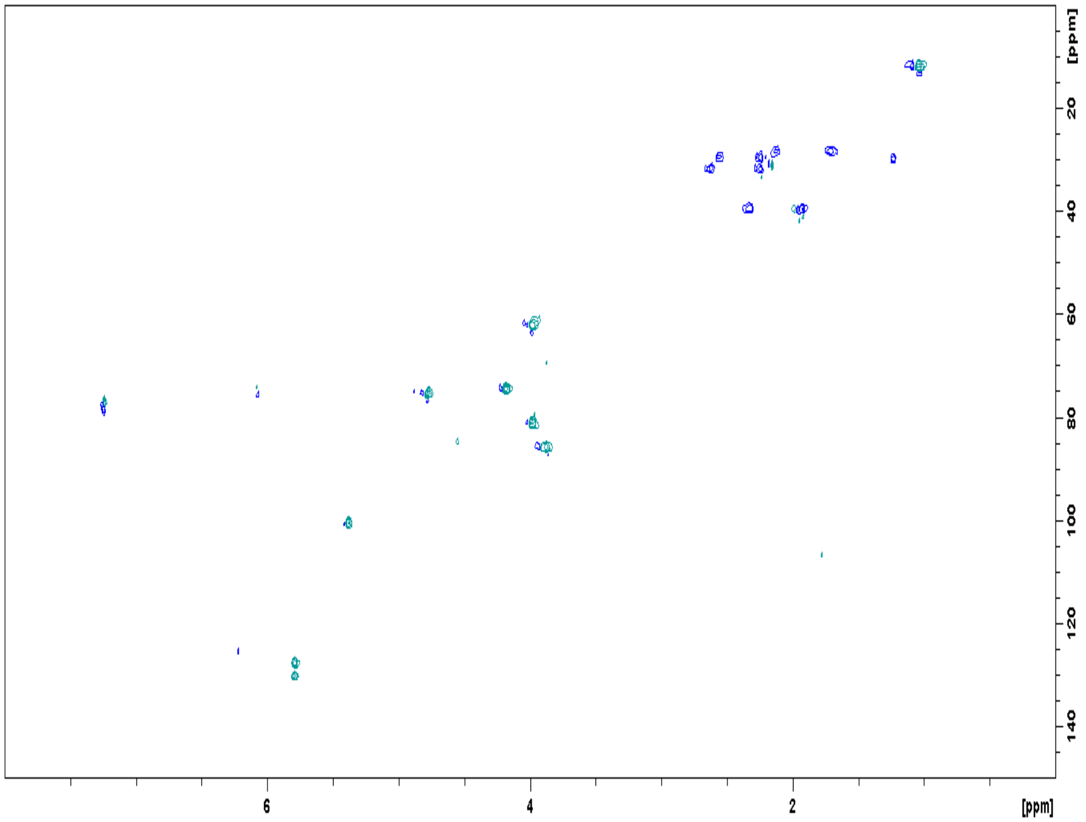

Figure S38. HSQC spectrum ($CDCl_3$, 400 MHz) of compound **12**.

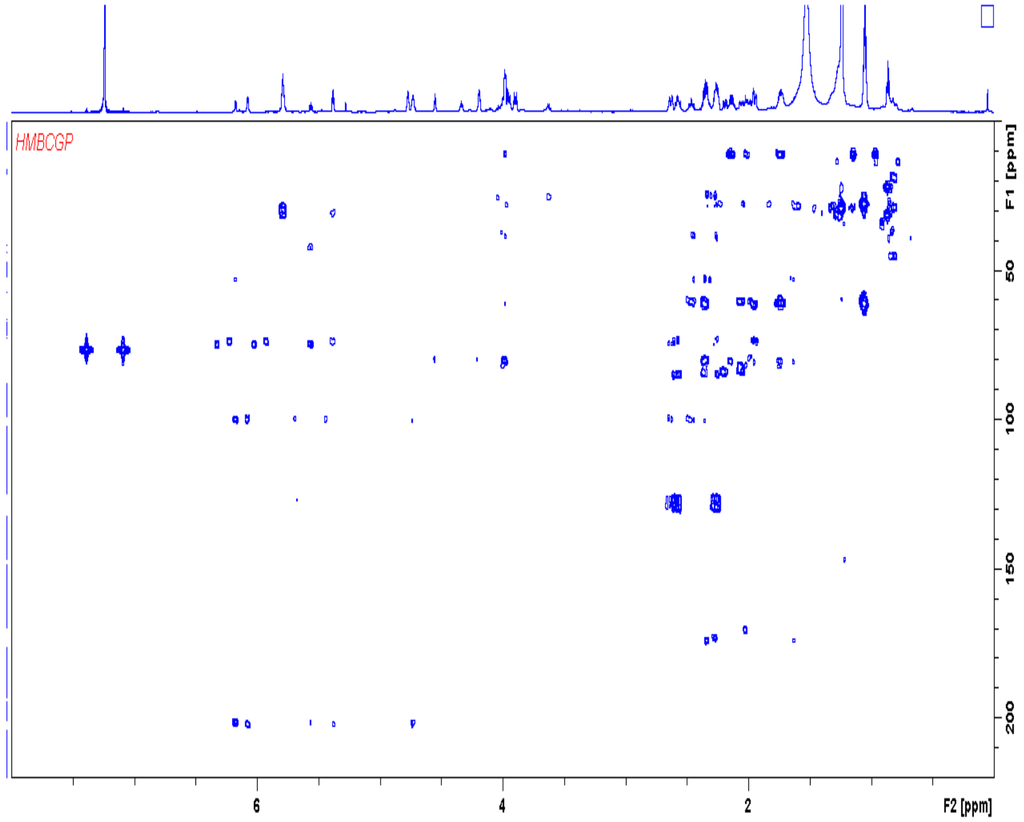

Figure S39. HMBC spectrum ($CDCl_3$, 700 MHz) of compound **12** (partial decomposition was already observed).

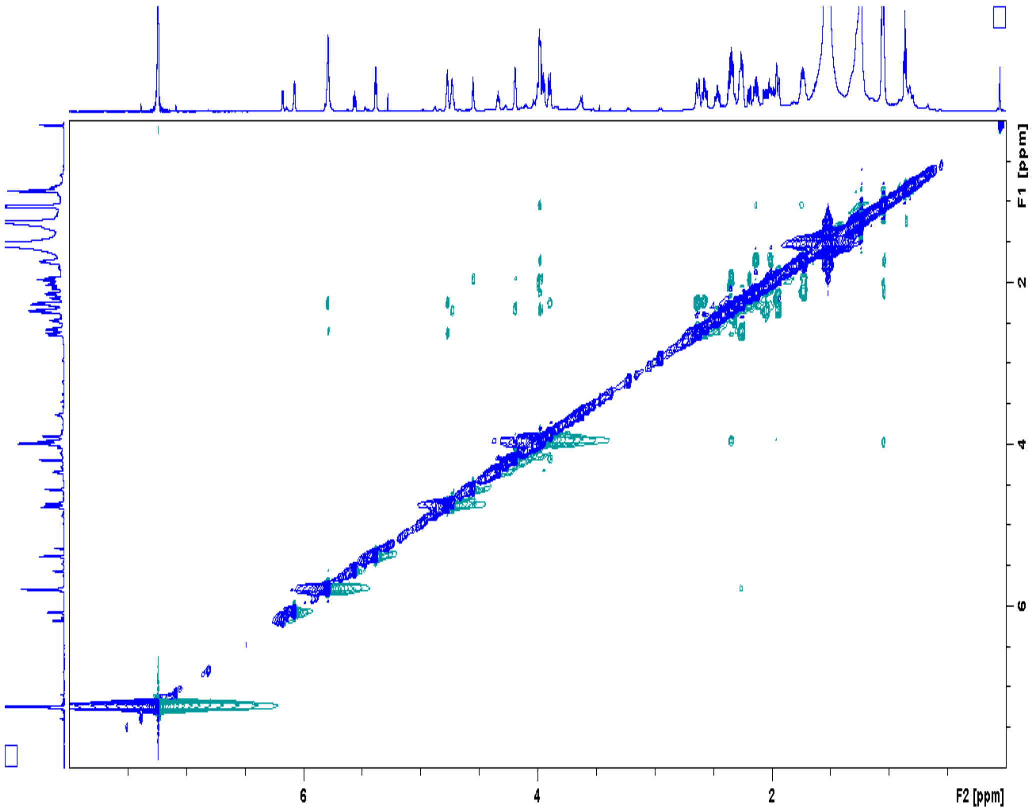

Figure S40. NOESY spectrum ($CDCl_3$, 700 MHz) of compound **12** (partial decomposition was already observed).

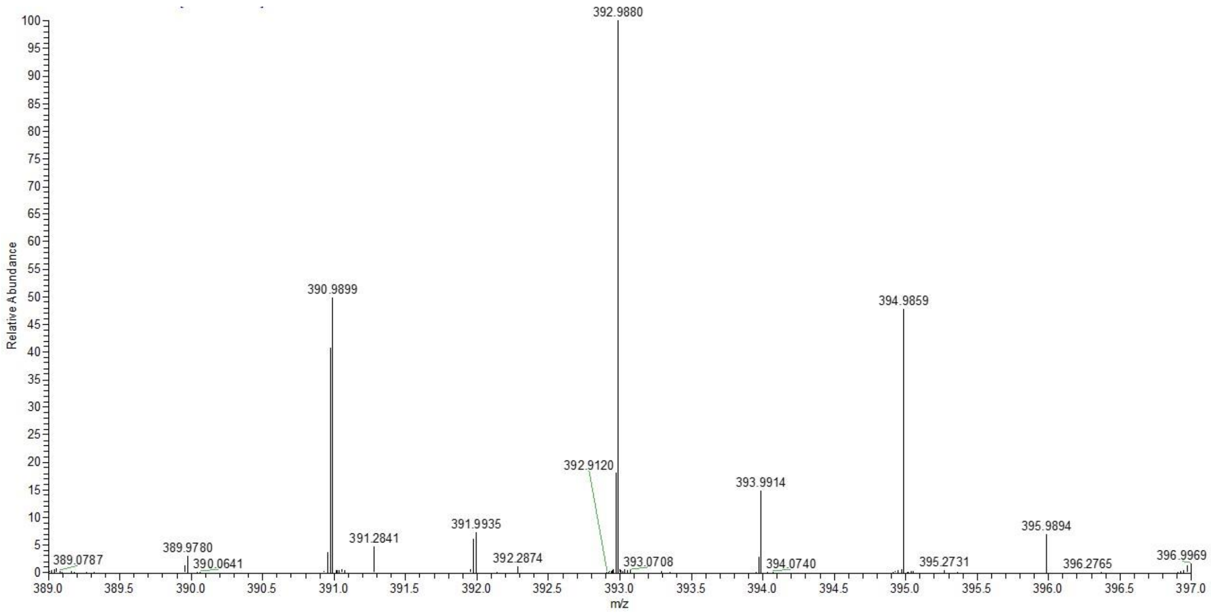

Figure S41. HR-APCIMS of compound **12**.

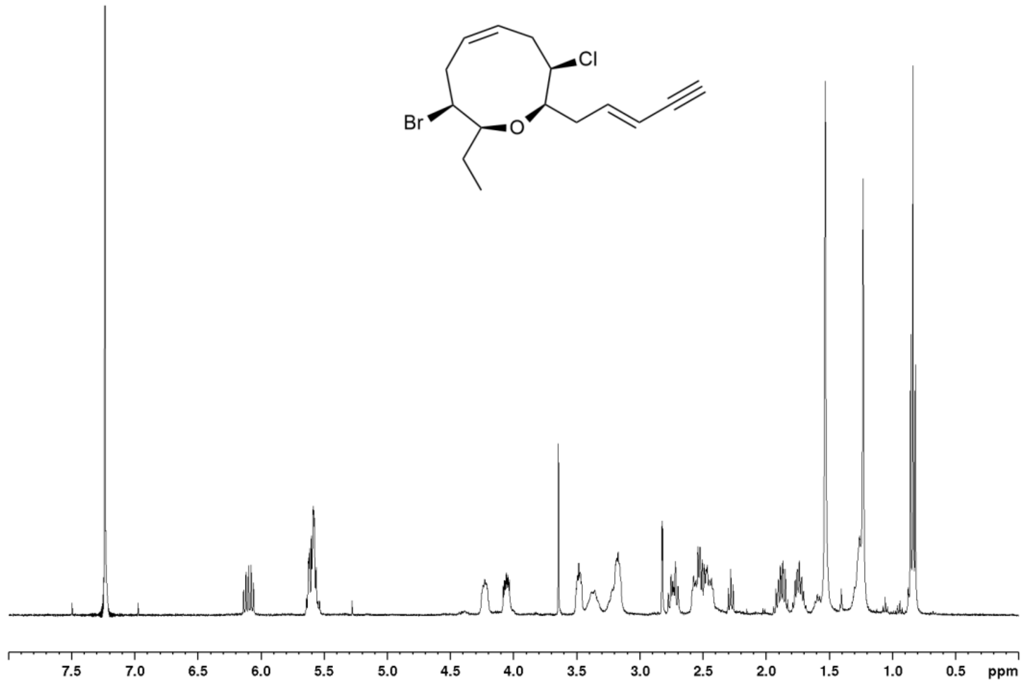

Figure S42. ^{1}H NMR spectrum ($CDCl_3$, 400 MHz) of compound **13**.

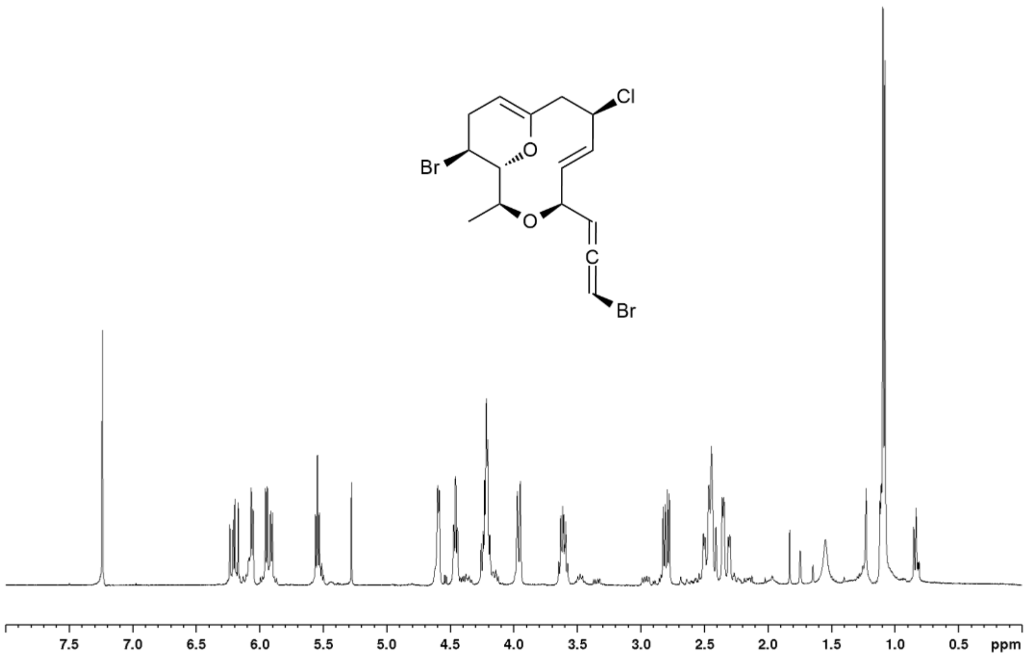

Figure S43. ^{1}H NMR spectrum ($CDCl_3$, 400 MHz) of compound **14**.

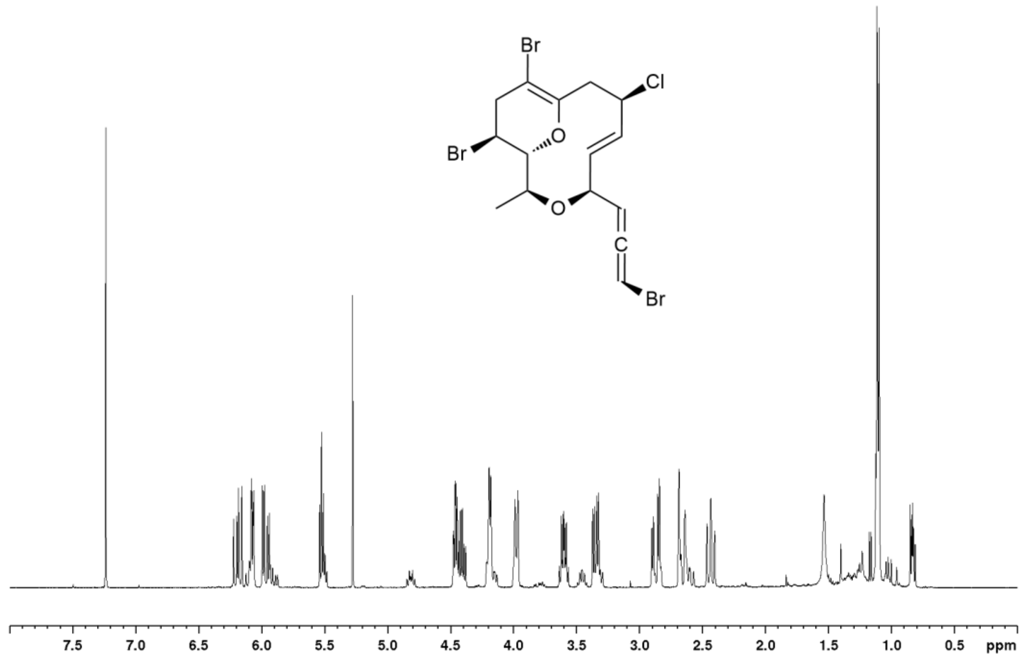

Figure S44. ^{1}H NMR spectrum ($CDCl_3$, 400 MHz) of compound **15**.

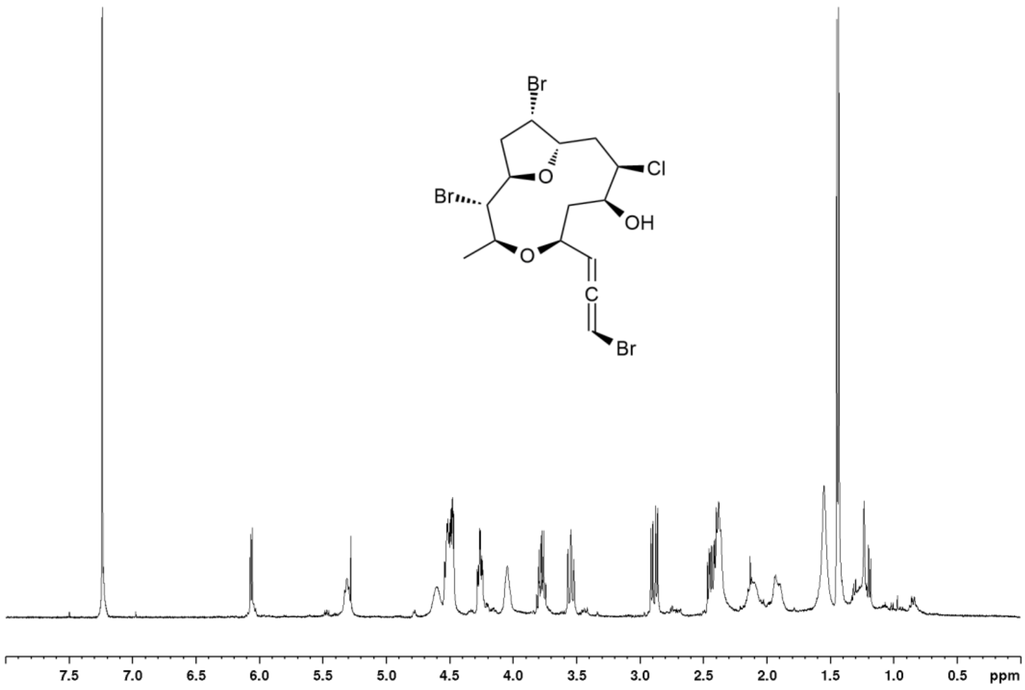

Figure S45. ^{1}H NMR spectrum ($CDCl_3$, 400 MHz) of compound **16**.

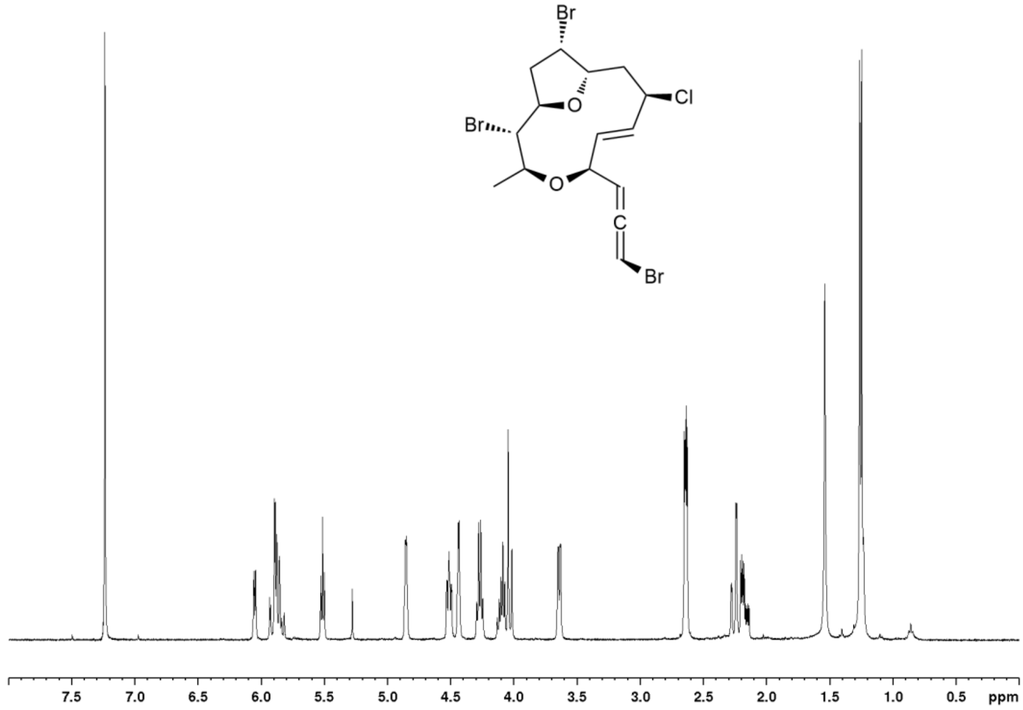

Figure S46. ^{1}H NMR spectrum ($CDCl_3$, 400 MHz) of compound **17**.

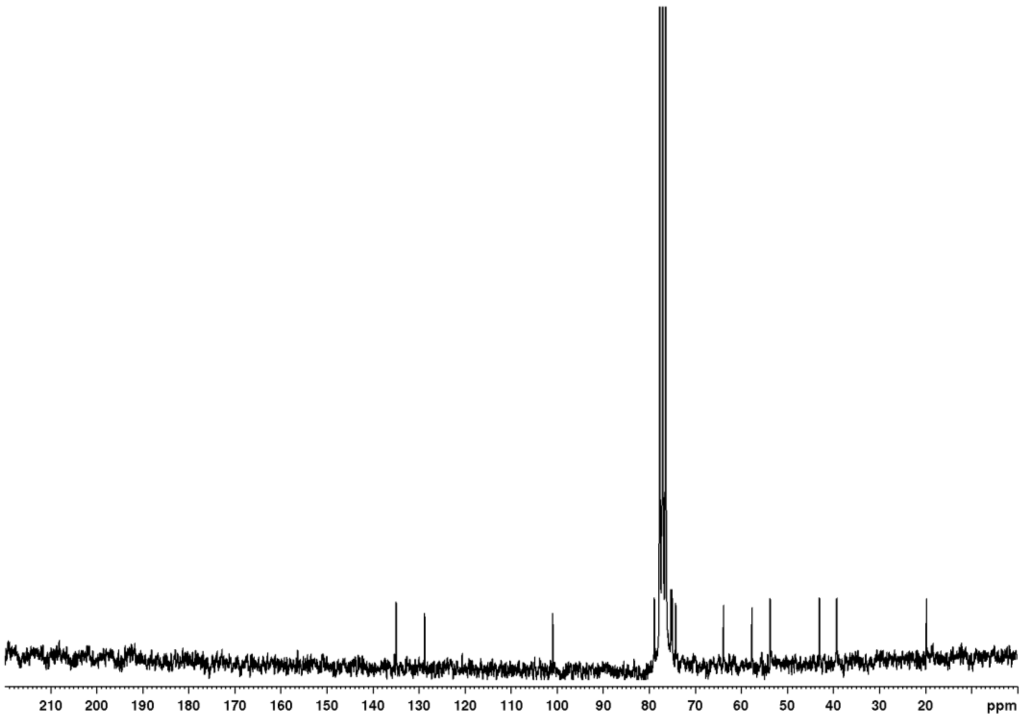

Figure S47. ^{13}C NMR spectrum ($CDCl_3$, 50.3 MHz) of compound **17**.

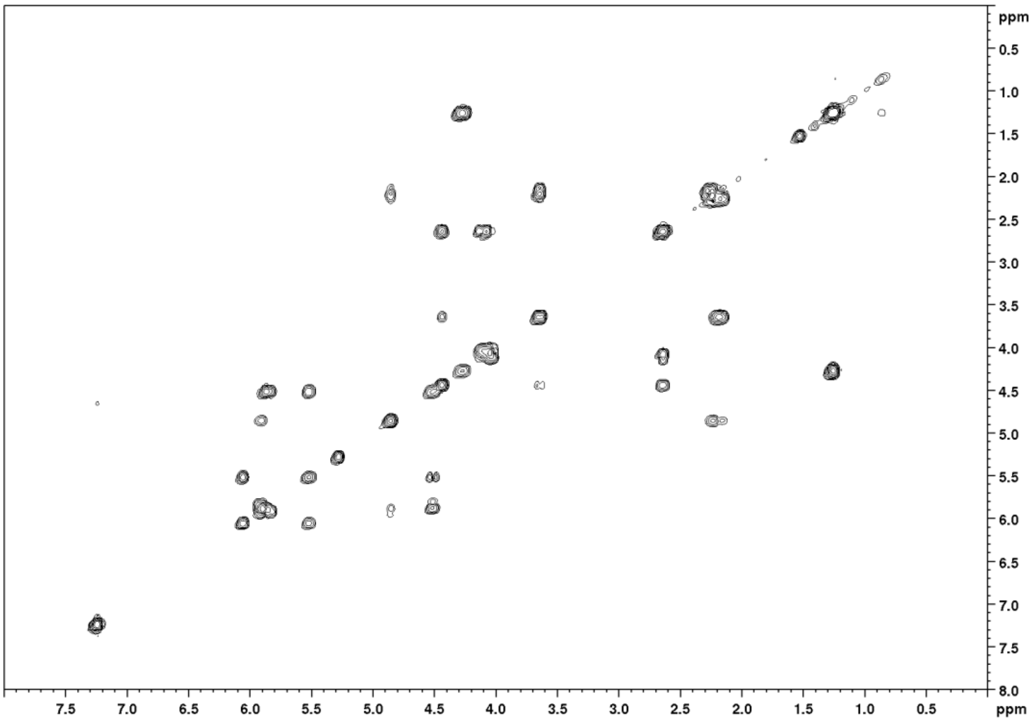

Figure S48. COSY spectrum ($CDCl_3$, 400 MHz) of compound **17**.

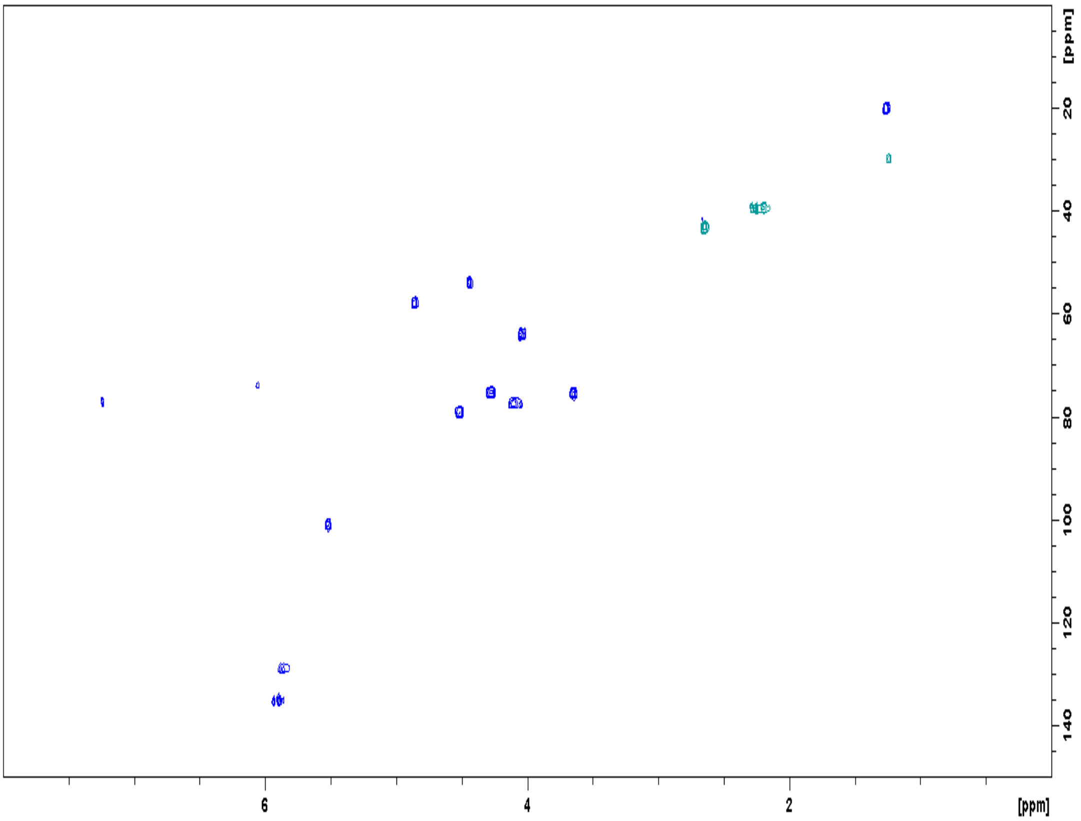

Figure S49. HSQC spectrum ($CDCl_3$, 400 MHz) of compound **17**.

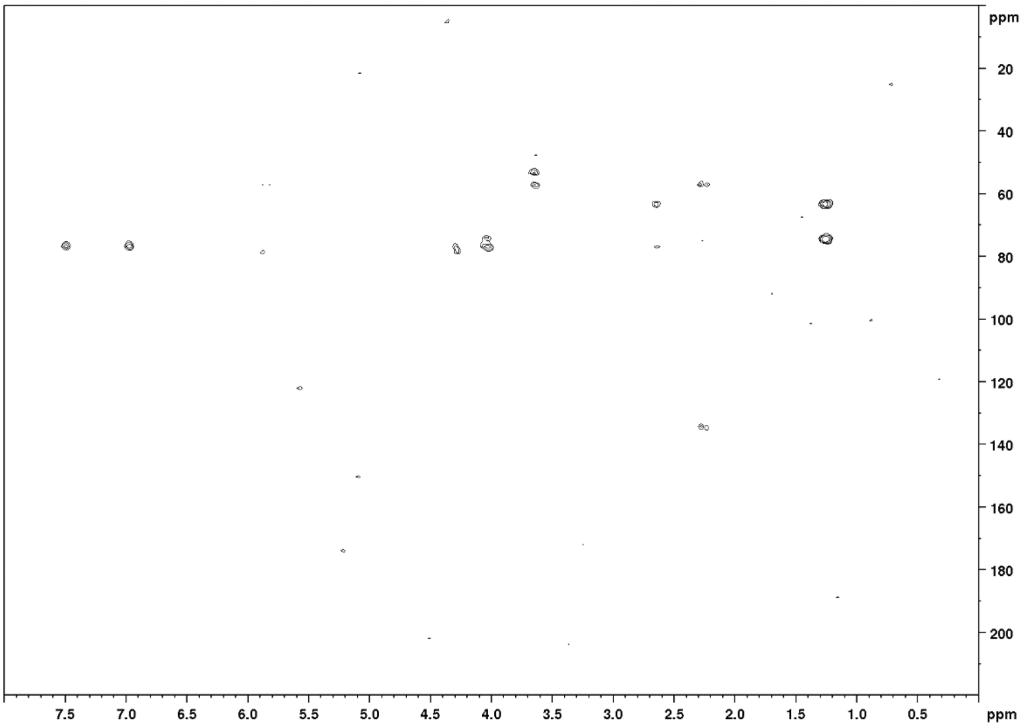

Figure S50. HMBC spectrum ($CDCl_3$, 400 MHz) of compound **17**.

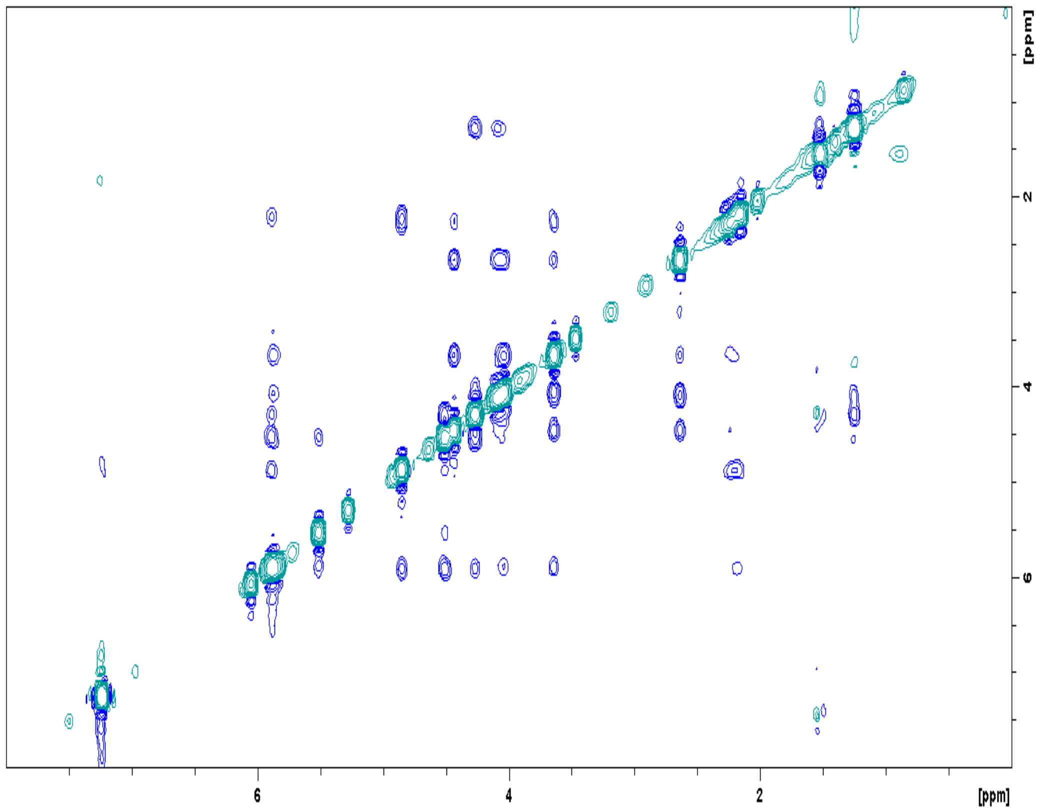

Figure S51. NOESY spectrum ($CDCl_3$, 400 MHz) of compound **17**.

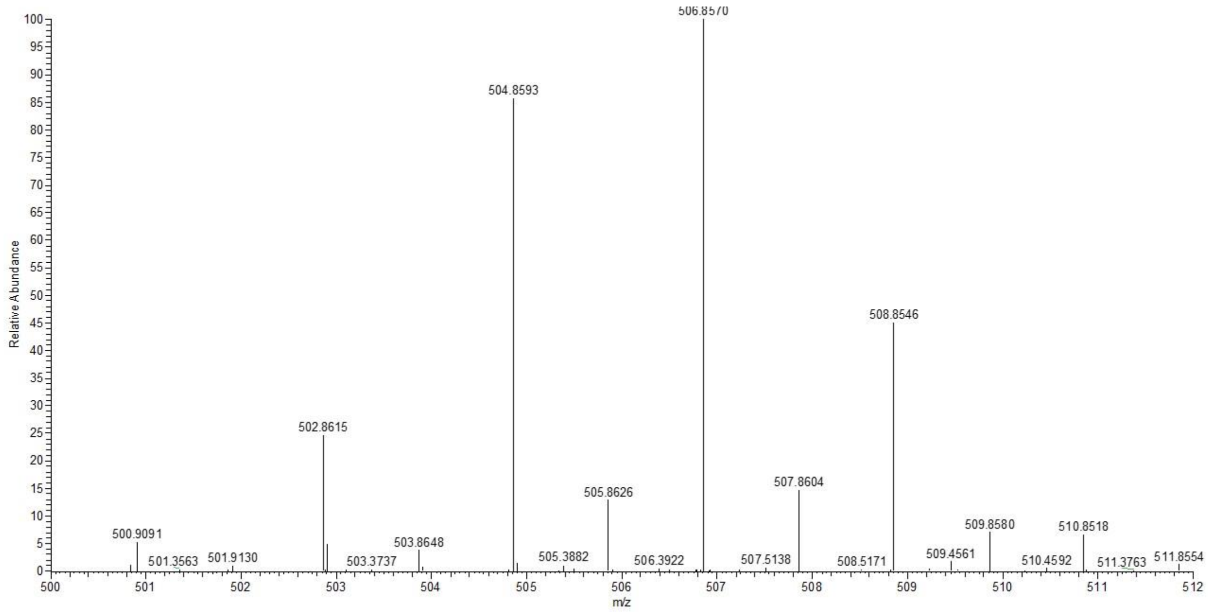

Figure S52. HR-APCIMS of compound **17**.

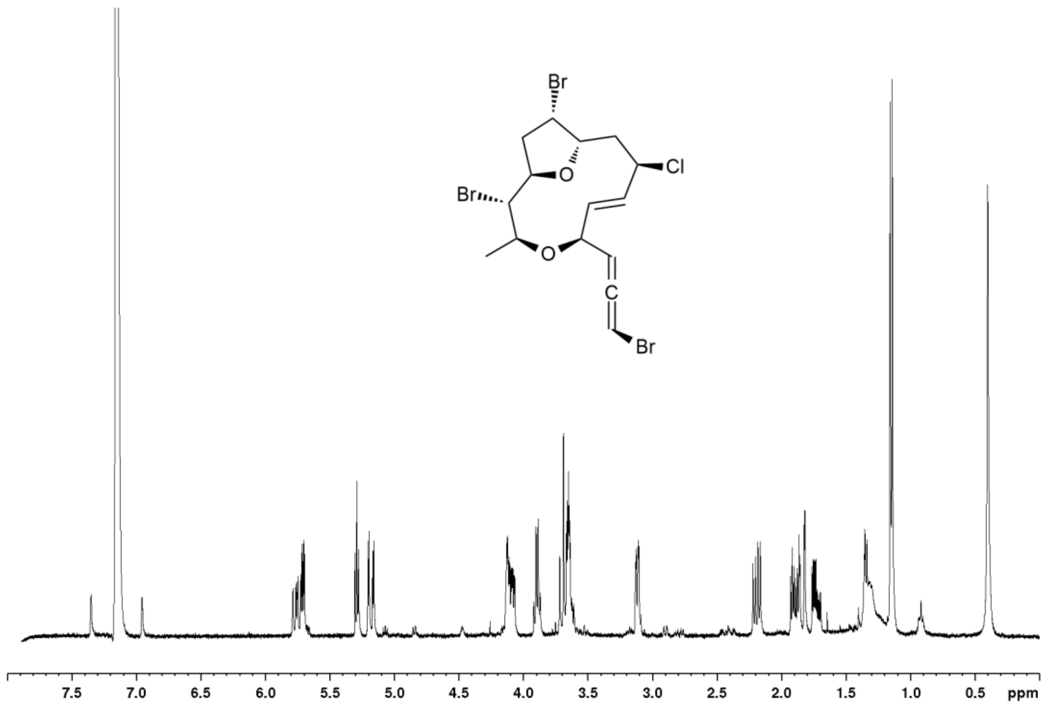

Figure S53. ^{1}H NMR spectrum (C_6D_6, 400 MHz) of compound **17**.

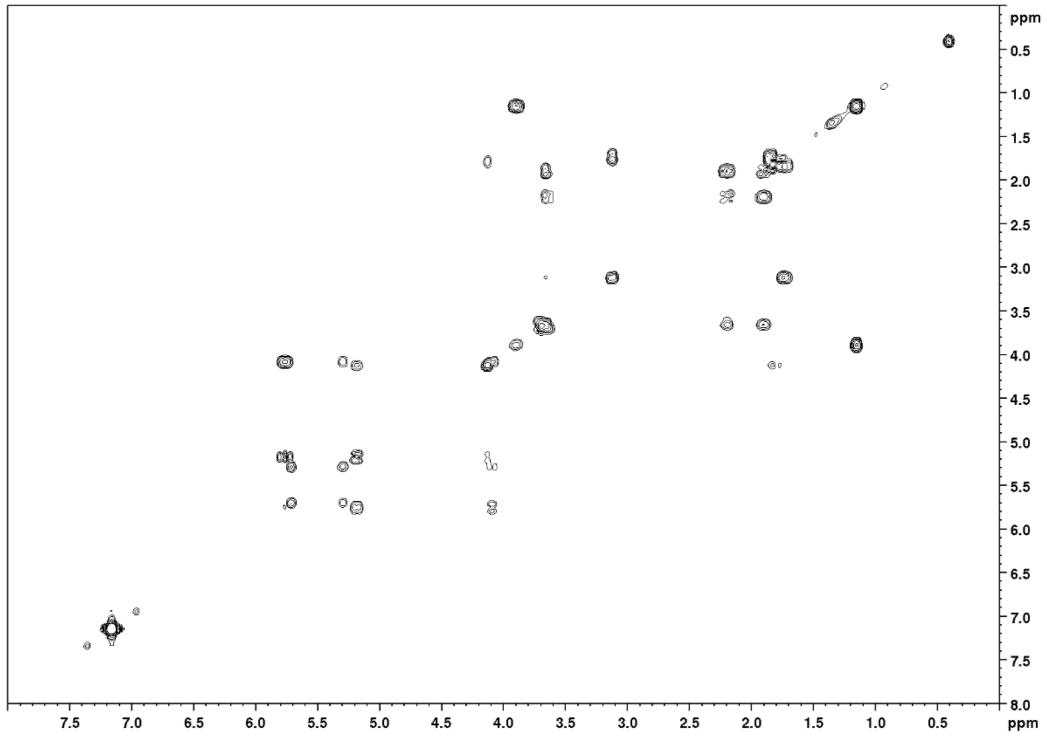

Figure S54. COSY spectrum (C_6D_6, 400 MHz) of compound **17**.

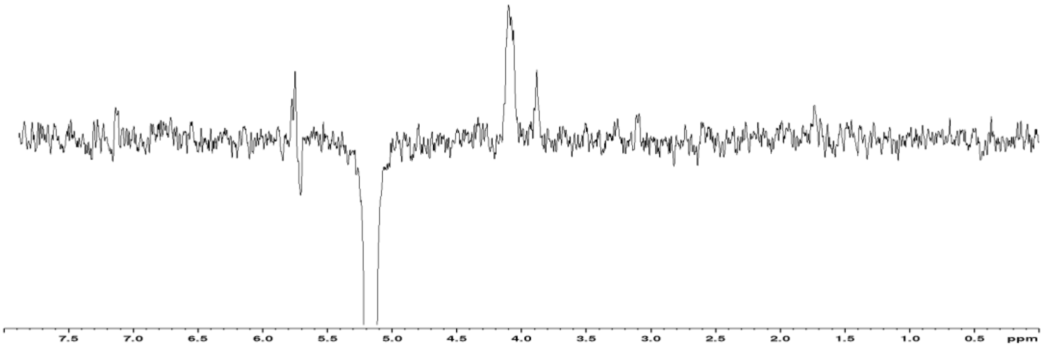

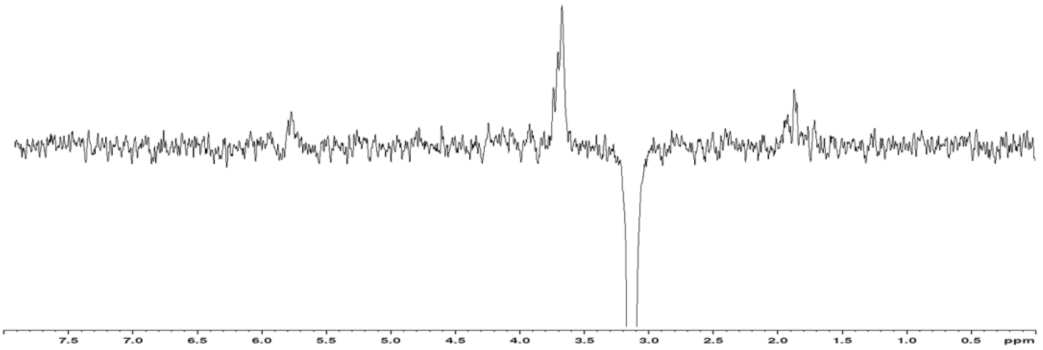

Figure S55. 1D-NOE spectra (C_6D_6, 400 MHz) of compound **17**.

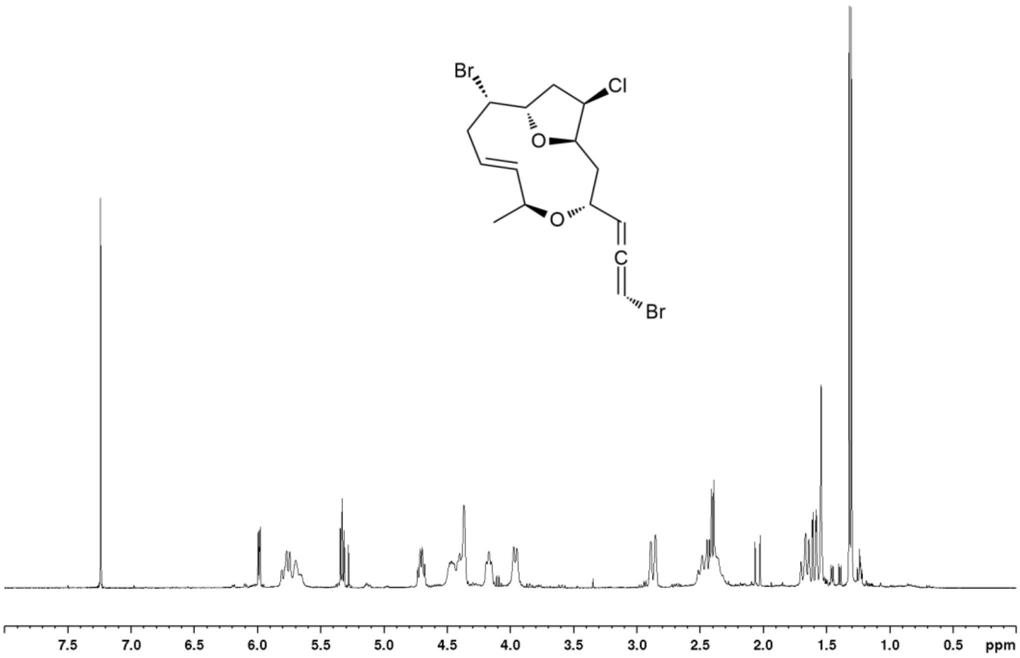

Figure S56. ^1H NMR spectrum ($CDCl_3$, 400 MHz) of compound **18**.

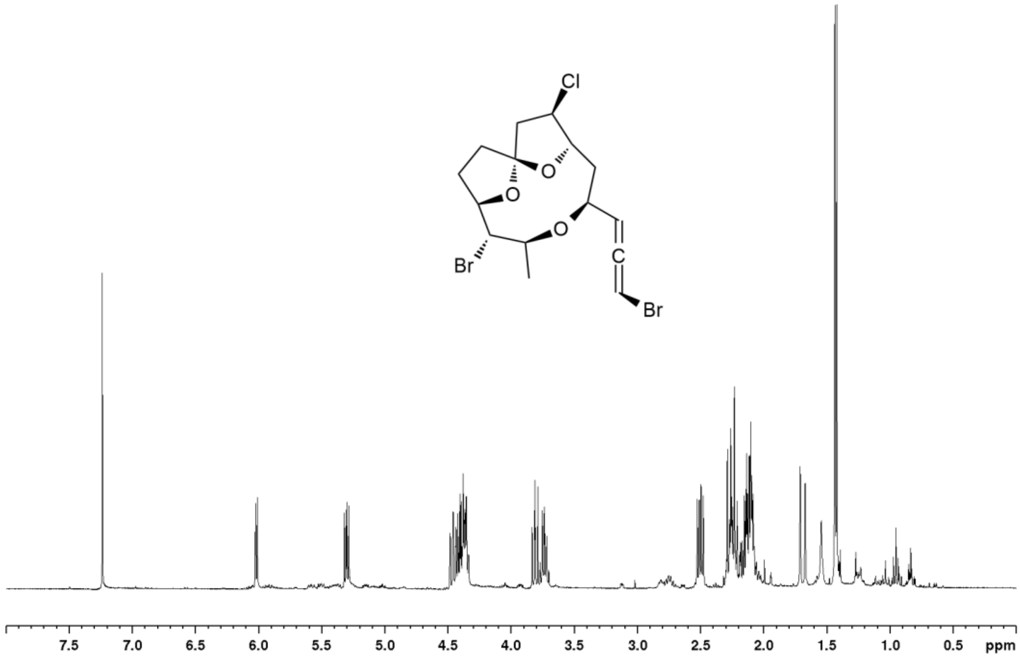

Figure S57. ^{1}H NMR spectrum ($CDCl_3$, 400 MHz) of compound **19**.

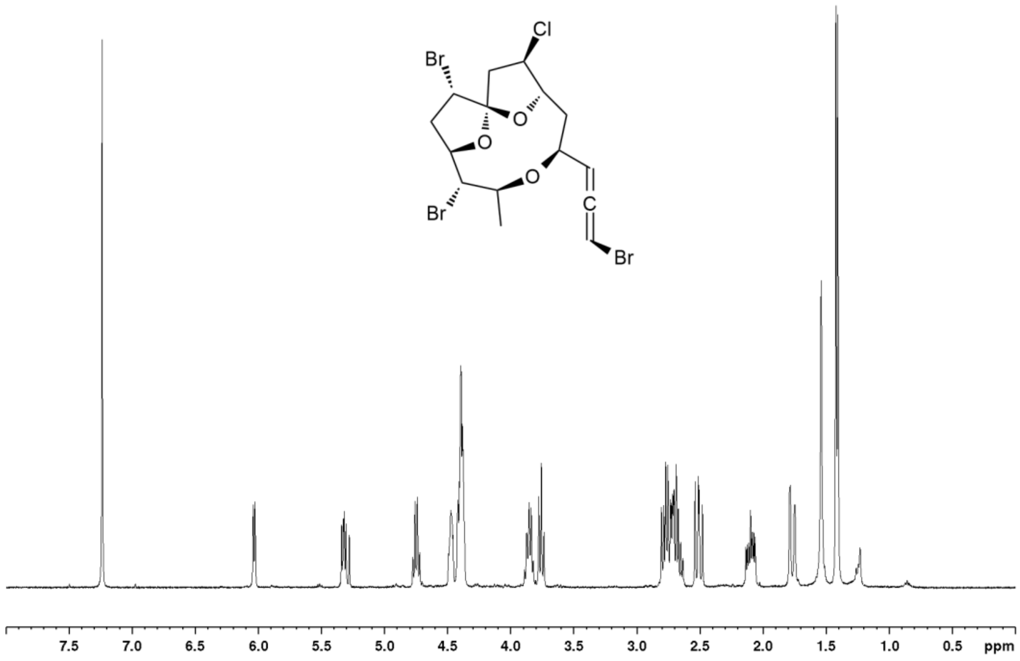

Figure S58. ^{1}H NMR spectrum ($CDCl_3$, 400 MHz) of compound **20**.

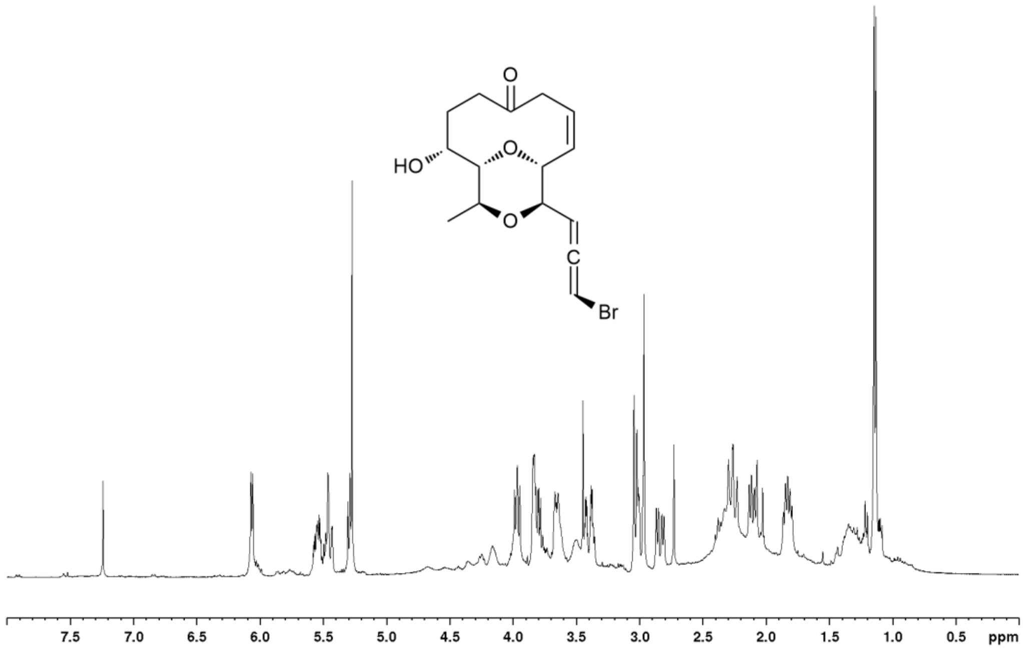

Figure S59. ^{1}H NMR spectrum ($CDCl_3$, 400 MHz) of compound **21**.

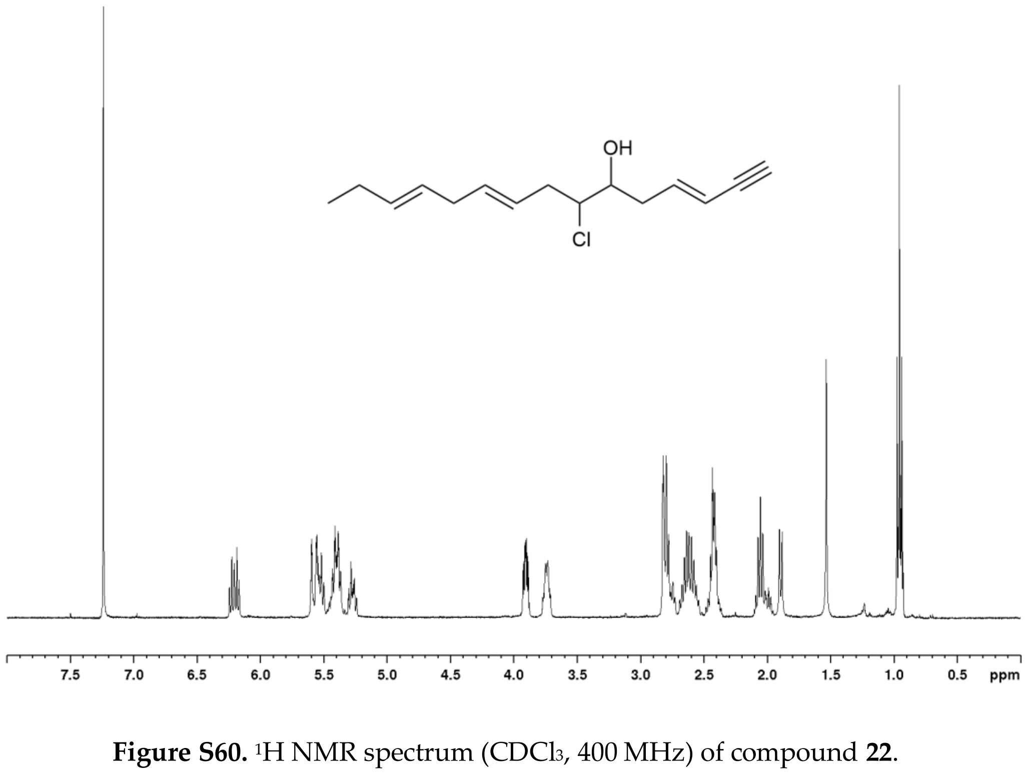

Figure S60. ^{1}H NMR spectrum ($CDCl_3$, 400 MHz) of compound **22**.

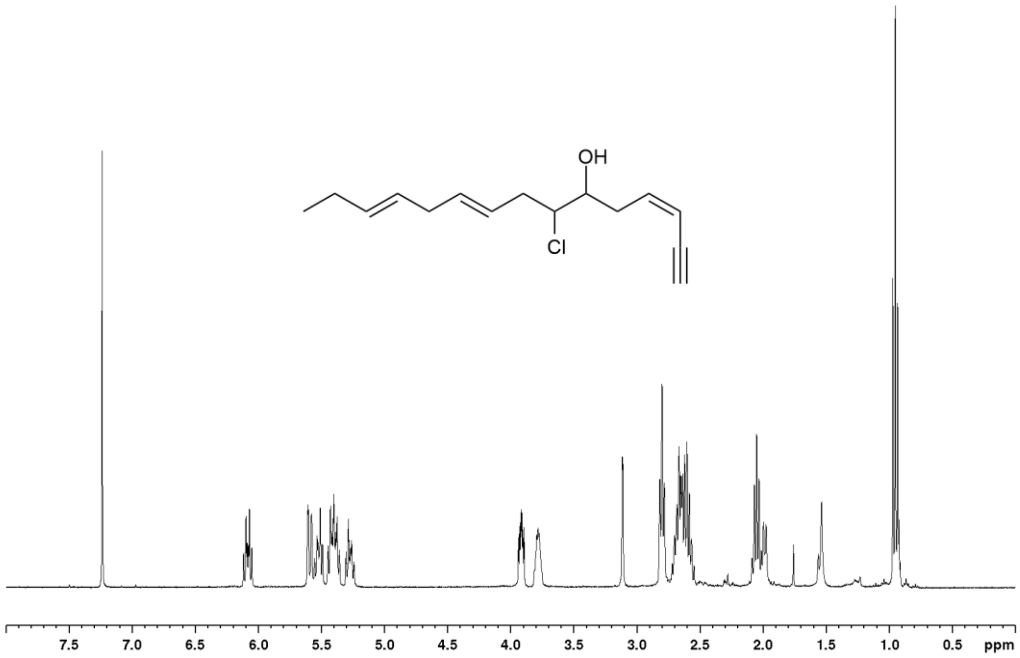

Figure S61. ^{1}H NMR spectrum ($CDCl_3$, 400 MHz) of compound **23**.

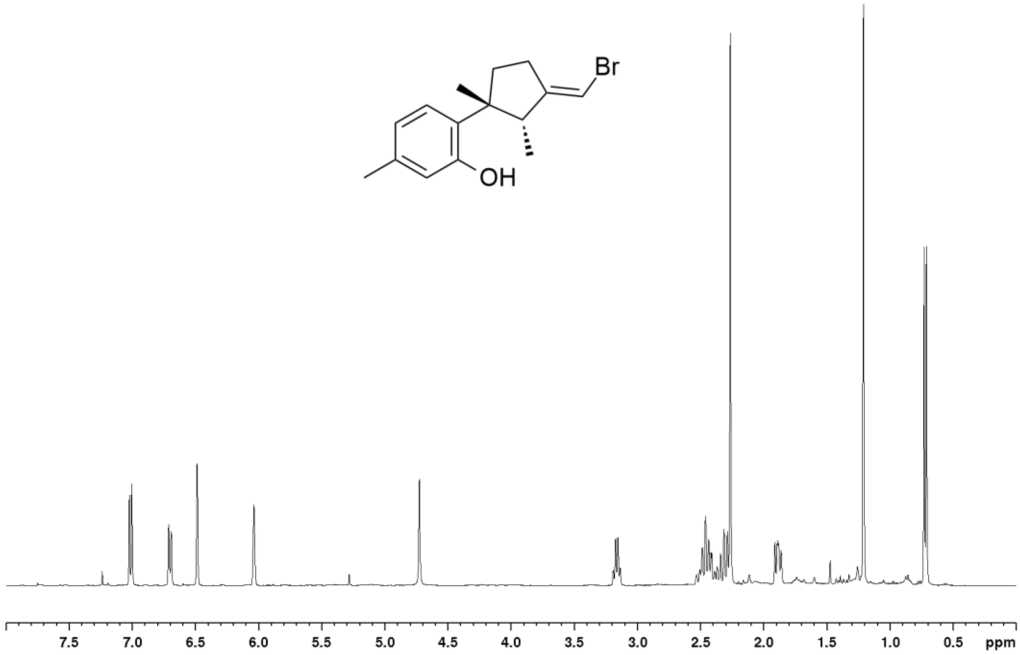

Figure S62. ^{1}H NMR spectrum ($CDCl_3$, 400 MHz) of compound **24**.

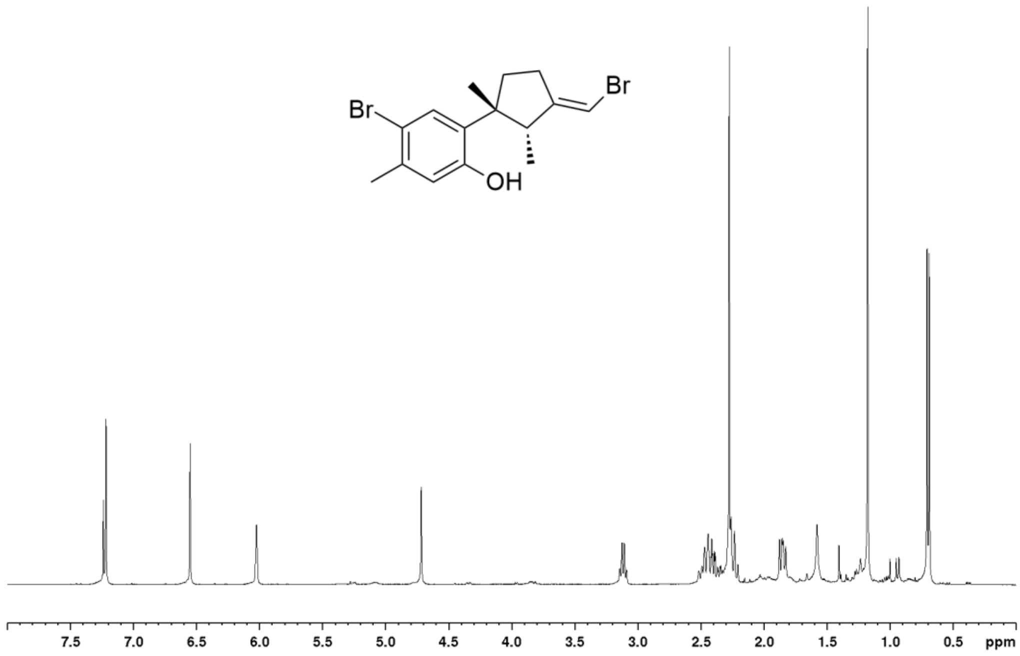

Figure S63. ^{1}H NMR spectrum ($CDCl_3$, 400 MHz) of compound **25**.

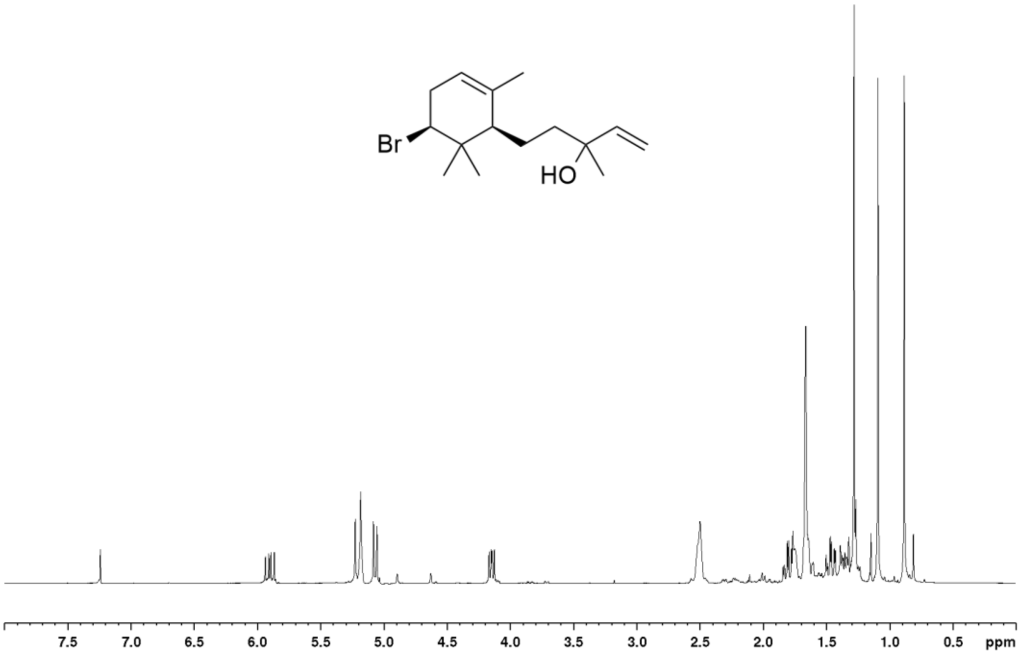

Figure S64. ^{1}H NMR spectrum ($CDCl_3$, 400 MHz) of compound **26**.

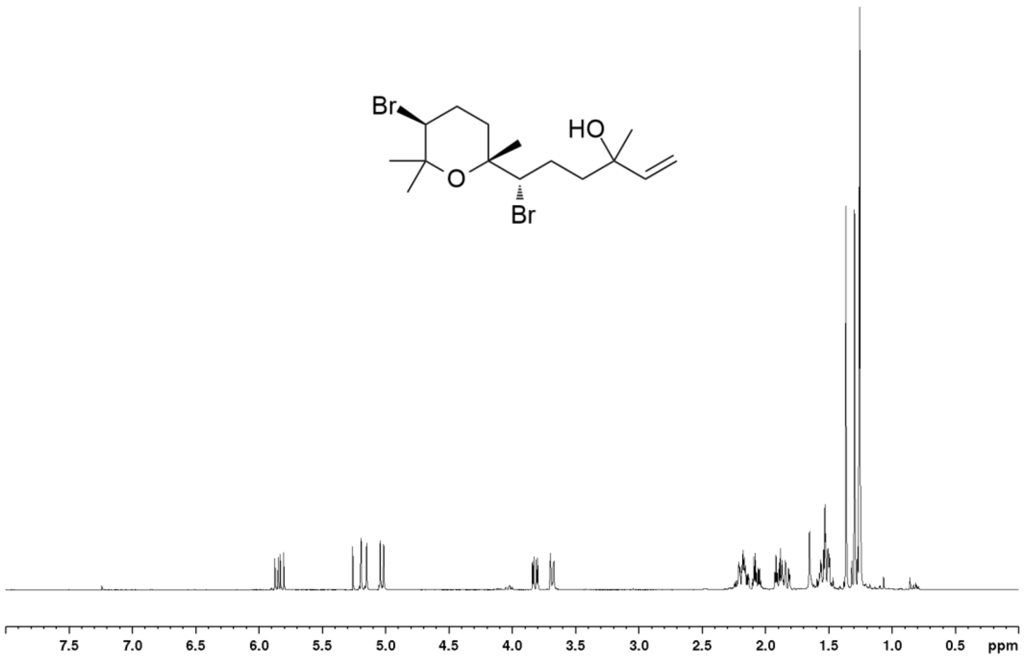

Figure S65. ^{1}H NMR spectrum ($CDCl_3$, 400 MHz) of compound **27**.

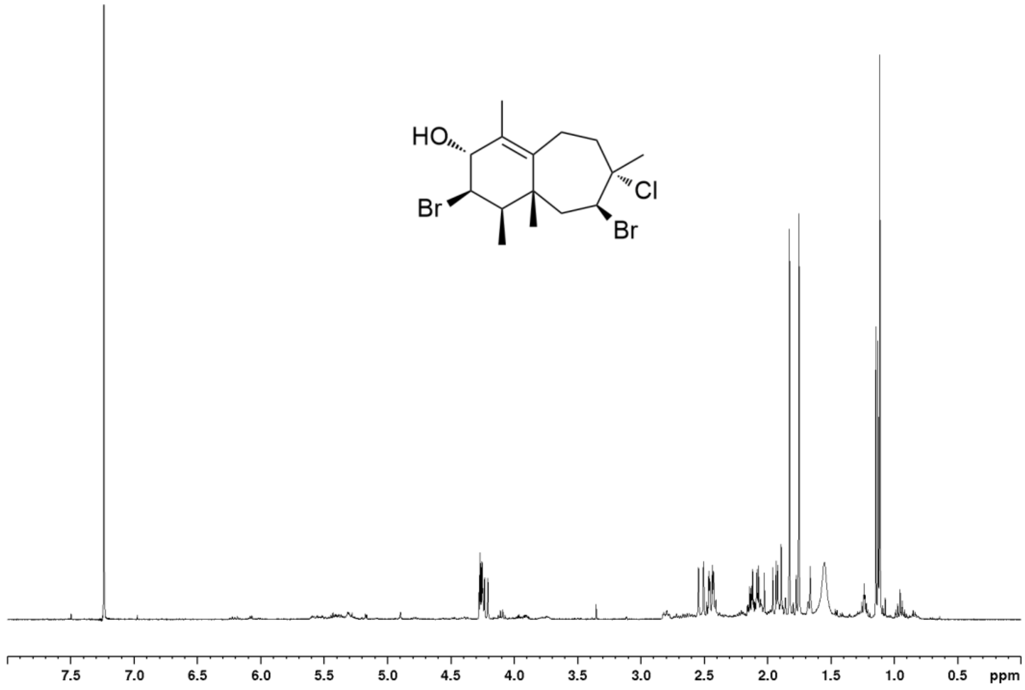

Figure S66. ^{1}H NMR spectrum ($CDCl_3$, 400 MHz) of compound **28**.

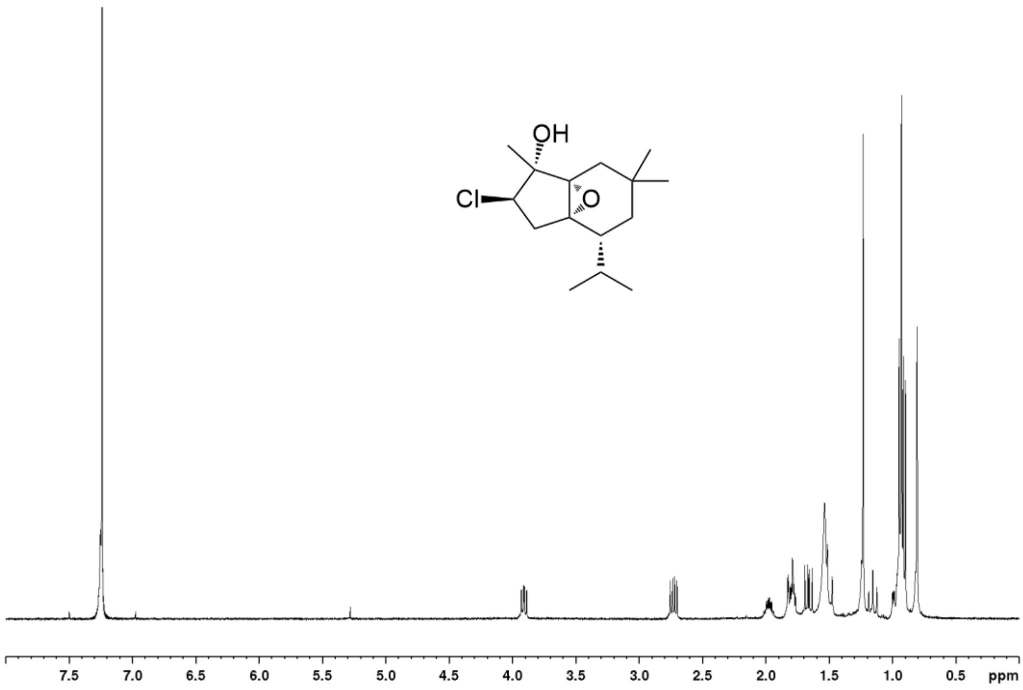

Figure S67. ^{1}H NMR spectrum ($CDCl_3$, 400 MHz) of compound **29**.

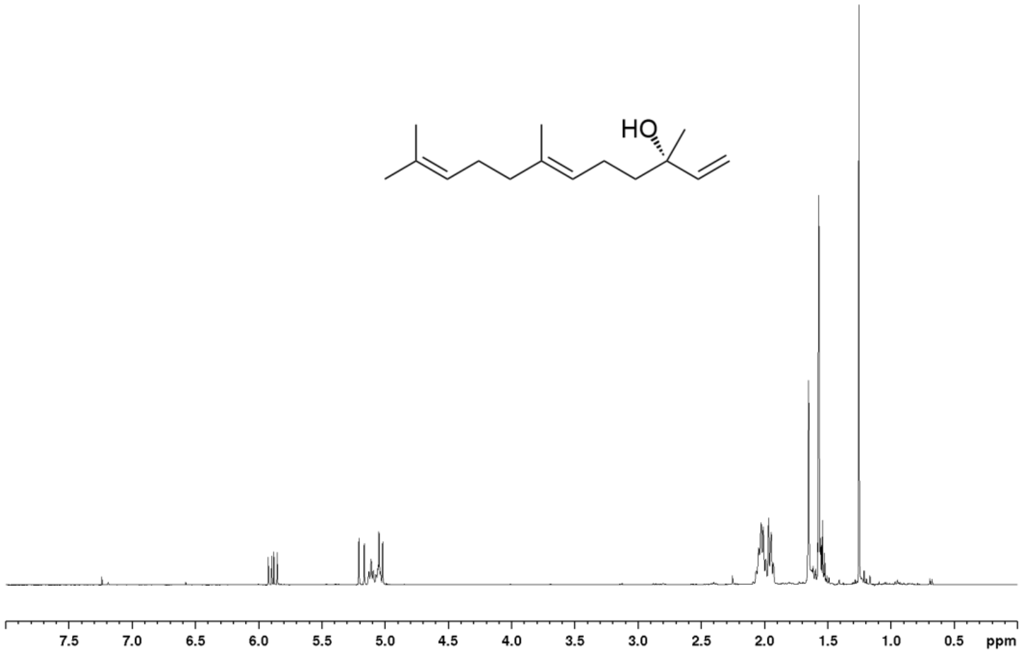

Figure S68. ^{1}H NMR spectrum ($CDCl_3$, 400 MHz) of compound **30**.

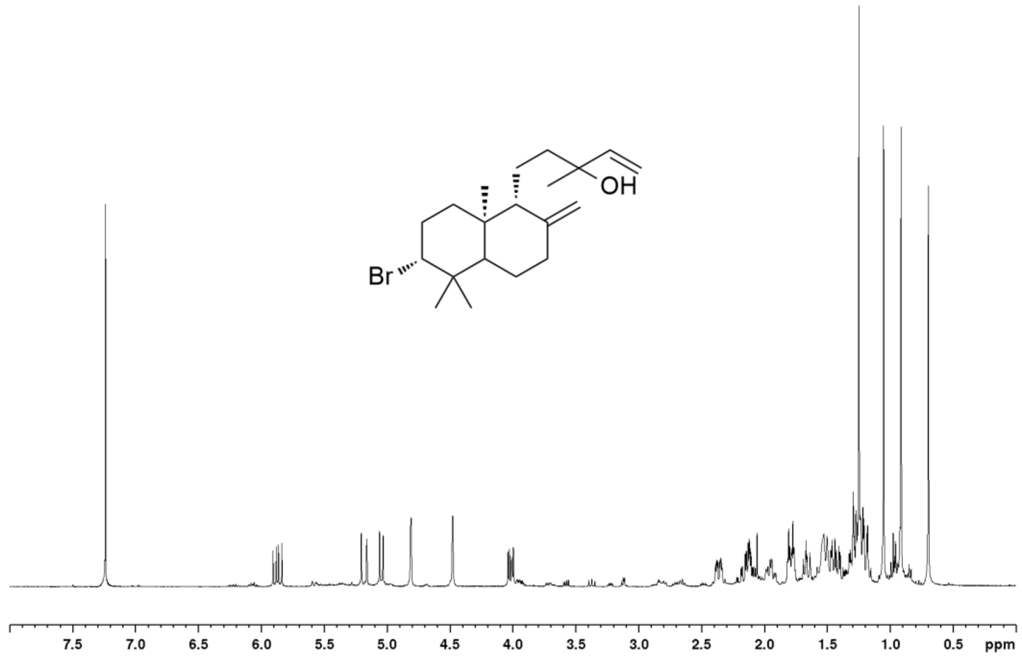

Figure S69. ^{1}H NMR spectrum ($CDCl_3$, 400 MHz) of compound **31**.

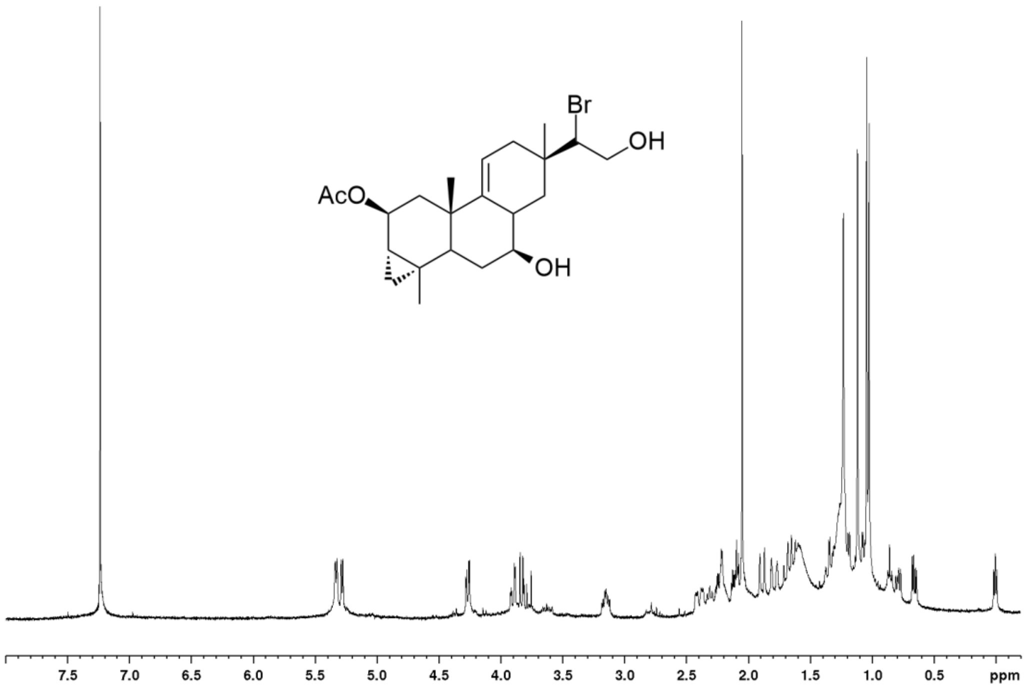

Figure S70. ^{1}H NMR spectrum ($CDCl_3$, 400 MHz) of compound **32**.
